# Erythritol alters gene transcriptome signatures, cell growth, and biofilm formation in *Staphylococcus pseudintermedius*

**DOI:** 10.1186/s12917-023-03711-3

**Published:** 2023-09-07

**Authors:** Tadashi Fujii, Takumi Tochio, Koji Nishifuji

**Affiliations:** 1 Research & Development Center, B Food Science Co., Ltd., Aichi, Japan; 2 Department of Gastroenterology and Hepatology, Fujita Health University, Aichi, Japan; 3 Division of Animal Life Science, Institute of Agriculture, Tokyo University of Agriculture and Technology, Tokyo, Japan

**Keywords:** Erythritol, *Staphylococcus pseudintermedius*, Transcriptome, Growth inhibition, Biofilm

## Abstract

**Background:**

Erythritol was found to inhibit the growth of microorganisms. The present study aimed to demonstrate the growth inhibition of *Staphylococcus pseudintermedius* by erythritol and to define the changes in gene transcription signatures induced by erythritol. Changes in the gene transcription profiles were analysed by RNA sequencing and quantitative reverse transcription PCR. Gene ontology analysis was performed to assign functional descriptions to the genes.

**Results:**

Erythritol inhibited *S. pseudintermedius* growth in a dose-dependent manner. We then performed a transcriptome analysis of *S. pseudintermedius* with and without 5% (w/w) erythritol exposure to validate the mechanism of growth inhibition. We revealed that erythritol induced up-regulation of three genes (*ptsG*, *ppdK*, and *ppdkR*) that are related to the phosphoenolpyruvate-dependent sugar phosphotransferase system (PTS). Glucose supplementation restored the up-regulation of the PTS-related genes in response to erythritol. In addition, erythritol down-regulated eleven genes that are located in a single *pur*-operon and inhibited biofilm formation of *S. pseudintermedius*.

**Conclusions:**

These findings indicated that erythritol antagonistically inhibits PTS-mediated glucose uptake, thereby exerting a growth inhibitory effect on *S. pseudintermedius*. Moreover, erythritol inhibits the ‘de novo’ IMP biosynthetic pathway that may contribute to biofilm synthesis in *S. pseudintermedius*.

**Supplementary Information:**

The online version contains supplementary material available at 10.1186/s12917-023-03711-3.

## Background

*Staphylococcus pseudintermedius* is a coagulase-positive, Gram-positive coccus that colonizes 90% of healthy dogs. It is an opportunistic pathogen and the most common cause of pyoderma and otitis externa in dogs [1]. Notably, the emergence of methicillin-resistant *S. pseudintermedius* (MRSP) has become a worldwide problem. Moreover, it has been reported that *S. pseudintermedius* is the cause of serious bacterial infections in immunosuppressed humans [2].

Erythritol is a sugar alcohol with four carbon atoms and is approximately 75% as sweet as sucrose; and the EU Scientific Committee on Food concluded in 2003 that it was safe to use in foods [3]. Erythritol was found to be more effective than xylitol, a sugar alcohol with one more carbon than erythritol, in inhibiting the growth of microorganisms, such as the caries-causing bacterium *Streptococcus mutans* [4], the indigenous oral bacterium *Streptococcus gordonii* [5], the periodontal disease bacterium *Porphyromonas gingivalis* [5], the causative bacteria of axillary and foot odor *Corynebacterium minutissimum*, *Corynebacterium striatum*, and *Staphylococcus epidermidis* [6], as well as the acne-causing bacterium *Cutibacterium acnes* [7].

The mechanisms of the biological effects of erythritol and xylitol have been studied in streptococci. A previous study proposed the following mechanisms for the growth inhibitory effects of xylitol against *S. mutans*: (*1*) direct inhibition of glycolytic enzymes by the intracellular accumulation of xylitol 5-phosphate derived from xylitol through the phosphoenolpyruvate (PEP)-dependent sugar phosphotransferase system (PTS), and (*2*) indirect inhibition by competition between glucose and xylitol for phosphate donors used in the PTS [8]. Another metabolomics study indicated that erythritol may inhibit the growth of *S. gordonii* and *P. gingivalis* by affecting various metabolic pathways, including nucleic acid synthesis and glycolytic pathways [5].

From these reports, it was speculated that the mechanism of growth inhibition by erythritol against staphylococci may also involve inhibition of the glucose metabolism pathway; however, no reports have clarified this possibility. This study was conducted to investigate the growth inhibition mechanism of erythritol against *S. pseudintermedius* using transcriptome analysis.

## Results

### Growth inhibitory effects of erythritol on *S. pseudintermedius*

**Fig. 1:**
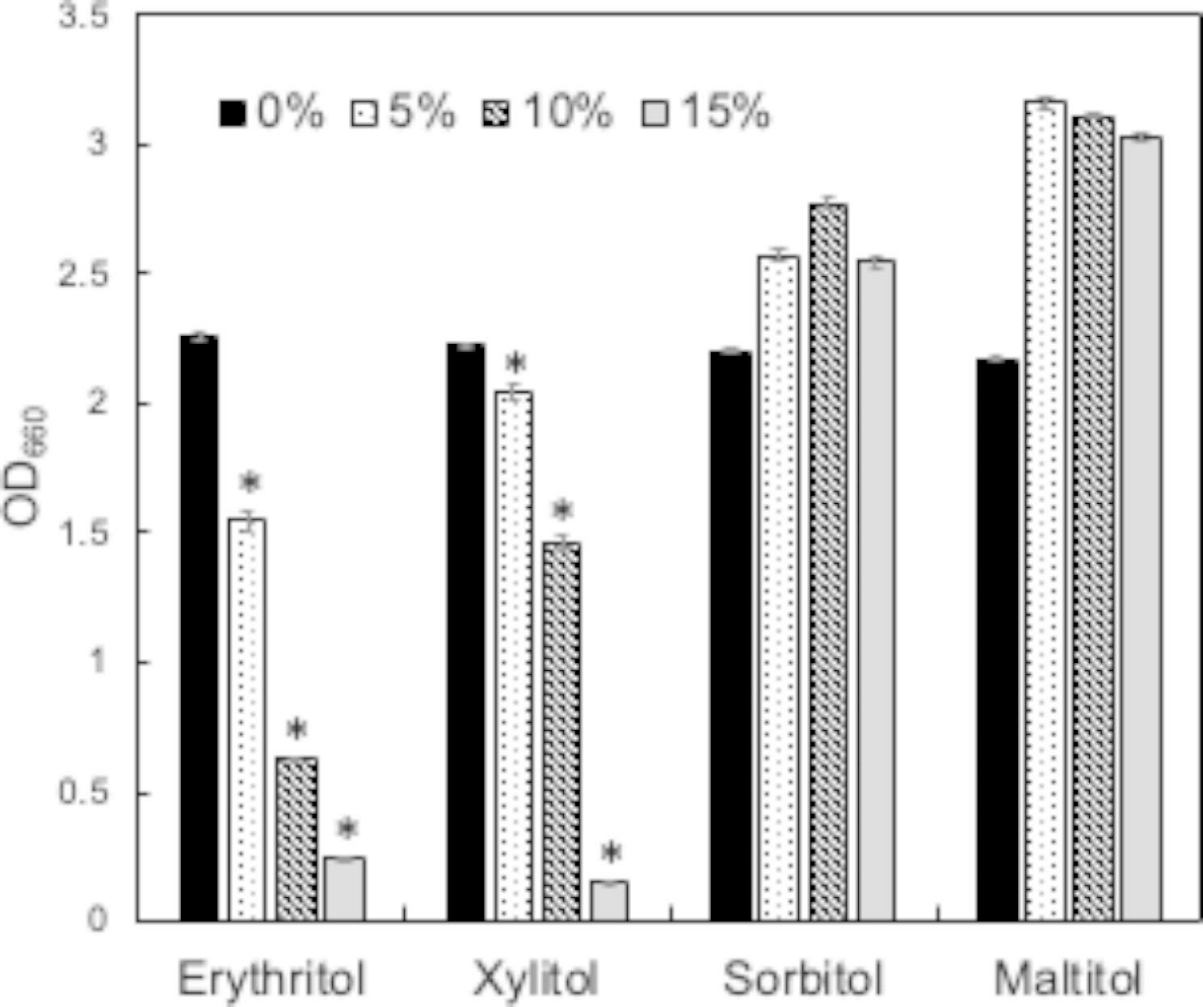
The turbidity (OD_660_) of the SP culture medium in the presence of 0, 5, 10, and 15% of each sugar (*n* = 4). The asterisk asterisk indicates *p* < 0.05 between the control (0%) group and each sugar-added group

The effects of the addition of sugar alcohols (erythritol, xylitol, sorbitol, and maltitol) on the growth of a type-strain of *S. pseudintermedius*, JCM 17,571 (SP), were investigated (Fig. 1). In the erythritol- and xylitol-added test groups, the addition of sugar alcohols (5, 10, and 15% [w/w]) significantly inhibited SP growth in a dose-dependent manner. In the sorbitol- and maltitol-added test groups, SP growth was not inhibited.

### Comprehensive gene expression analysis of erythritol-treated *S. pseudintermedius*

**Fig. 2:**
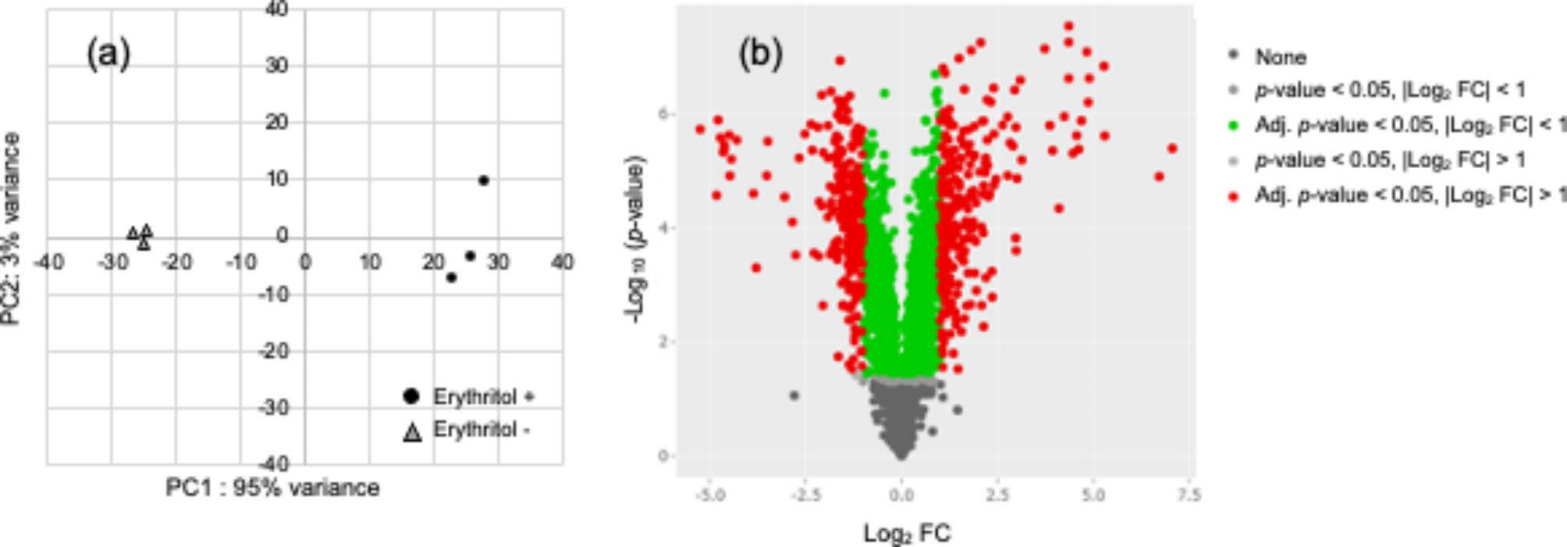
RNA-seq analysis and verification by NASQAR. (**a**) PCA shows clustering of RNA-seq samples with or without erythritol treatment. (**b**) Volcano plot showing RNA-seq samples

To examine the mechanism of the inhibitory effect of erythritol on the growth of SP, comprehensive gene expression analysis using RNA sequencing (RNA-seq) was performed for SP cultured in the presence or absence of erythritol (*n* = 3). To avoid complete suppression of cellular metabolism, a weak inhibiting concentration (5% [w/w]) was selected. After filtering the raw reads, we found that there were 19,142,672, 20,940,942, and 22,024,908 clean reads of the transcriptome in the control samples, whereas 19,068,865, 19,060,494, and 19,393,064 clean reads were obtained from the erythritol-treated samples. A complete list of all reads is shown in Additional file 1. Among these genes, a total of 625 differentially expressed genes were identified using the calculated gene expression levels (|Log_2_ FC (fold change)| > 1, *p*-value < 0.05), with 244 genes markedly up-regulated and 381 genes markedly down-regulated in SP following erythritol treatment. A Principal Component Analysis (PCA) plot showed a clear split between the control and erythritol-treated samples, with 95% of the variance explained by PC1 (Fig. 2a). A volcano plot showed that the differential expressions of the top up-regulated and down-regulated genes were statistically significant (Fig. 2b).

### The top 15 up-regulated and top 15 down-regulated genes in response to erythritol

The predicted functional descriptions and Gene Ontology (GO) biological process classes for the top 15 up-regulated and top 15 down-regulated genes in response to erythritol are shown in Table 1. Among the top 15 up-regulated genes with a predicted GO biological process class, genes related to two biological functional groups were present. Three genes (Up-Regulated Genes group 1, *URGs1*), BJK46_009280 (*ptsG*), BJK46_000300 (*ppdK*), and BJK46_000295 (*ppdkR*), were predicted to encode proteins related to the PTS. The *ptsG* gene encodes the glucose specific EIICBA component of the PTS (PtsG), and the *ppdK* and *ppdkR* genes encode pyruvate phosphate dikinase (PPDK) and PPDK regulatory protein (PPDKR), respectively, which are involved in the regeneration of PEP that is required to drive the PTS. Moreover, the other four genes (*URGs2*), BJK46_008080 (*vraT*), BJK46_008075 (*vraS*), BJK46_008070 (*vraR*), and BJK46_008015 (*sgtB*) were predicted to belong to the same group. The *vraTSR* genes belonging to a single gene cluster are predicted to comprise a three-component regulatory system necessary to promote resistance to cell wall agents, and *sgtB* is predicted to encode a glycosyltransferase associated with peptidoglycan biosynthesis. The GO classes of the biological process could not be predicted in the remaining 8 up-regulated genes.

**Table 1:** Top 15 up-regulated genes (top table) and top 15 down-regulated genes (bottom table) by erythritol

Log_2_FC	*p*-value	Gene_id	Gene product	Gene name	Log_2_FC		p-value		
					Estimated PPV (> 0.5)	Description	Estimated PPV (> 0.5)	GO-id	Description
7.01	0	BJK46_006305	hypothetical protein						
6.99	9.00E-300	BJK46_000955	hypothetical protein						
5.39	0	BJK46_002585	DUF1361 domain-containing protein		0.52	DUF1361 domain-containing protein (fragment)			
5.24	0	BJK46_008880	DUF5011 domain-containing protein		0.51	chitinase			
4.76	1.42E-302	BJK46_008085	hypothetical protein						
4.73	0	BJK46_005200	M50 family peptidase		0.67	M50 family metallopeptidase			
4.63	2.45E-302	BJK46_009280	PTS glucose transporter subunit IICBA	*ptsG*	0.72	PTS system glucose-specific EIICBA component	0.82	GO:1,904,659	glucose transmembrane transport
							0.72	GO:0009401	phosphoenolpyruvate-dependent sugar phosphotransferase system
							0.59	GO:0016310	phosphorylation
4.62	2.79E-249	BJK46_006810	LytR family transcriptional regulator						
4.5	6.13E-307	BJK46_008015	glycosyltransferase	*sgtB*	0.50	monofunctional glycosyltransferase	0.66	GO:0008360	regulation of cell shape
							0.66	GO:0009252	peptidoglycan biosynthetic process
							0.66	GO:0071555	“
4.47	3.41E-230	BJK46_000295	kinase/pyrophosphorylase	*ppdkR*		Putative pyruvate, phosphate dikinase regulatory protein	0.70	GO:0006470	protein dephosphorylation
							0.63	GO:0006468	protein phosphorylation
4.22	2.28E-202	BJK46_000300	pyruvate, phosphate dikinase	*ppdK*	0.63	Pyruvate, phosphate dikinase	0.67	GO:0006090	pyruvate metabolic process
							0.59	GO:0016310	phosphorylation
4.15	7.55E-274	BJK46_008070	DNA-binding response regulator	*vraR*			0.67	GO:2,000,112	regulation of cellular macromolecule biosynthetic process
							0.63	GO:0000160	phosphorelay signal transduction system
							0.58	GO:0006355	regulation of transcription, DNA-templated
4.14	2.29E-274	BJK46_008080	transporter	*vraT*	0.69	Transporter yvqF			
4.1	5.04E-252	BJK46_008075	sensor histidine kinase	*vraS*	0.89	Sensor protein VraS	0.68	GO:0018106	peptidyl-histidine phosphorylation
							0.63	GO:0000160	phosphorelay signal transduction system
4.1	5.40E-246	BJK46_000760	DUF1002 domain-containing protein		0.79	Extracellular protein			
**Log**_**2**_ **FC**	***p*** **-value**	**Gene_id**	**Gene product**	***Gene*** ***name***	**Description (shown by Pannzer2)**		**Biological process (shown by Pannzer2)**		
					**Estimated PPV (> 0.5)**	**Description**	**Estimated PPV (> 0.5)**	**GO-id**	**Description**
-5.33	0	BJK46_009895	hypothetical protein		0.59	surface rod structure-forming protein G (fragment)			
-4.84	2.28E-219	BJK46_012045	hypothetical protein						
-4.8	0	BJK46_008825	phosphoribosylglycinamide formyltransferase	*purN*	0.56	phosphoribosylglycinamide formyltransferase			
-4.79	0	BJK46_008820	bifunctional phosphoribosylaminoimidazolecarboxamide formyltransferase/IMP cyclohydrolase	*purH*	0.54	bifunctional purine biosynthesis protein PurH			
-4.69	0	BJK46_008830	phosphoribosylformylglycinamidine cyclo-ligase	*purM*	0.67	phosphoribosylformylglycinamidine cyclo-ligase			
-4.67	0	BJK46_008835	Amidophosphoribosyltransferase	*purF*	0.63	amidophosphoribosyltransferase	0.75	GO:0009113	purine nucleobase biosynthetic process
							0.70	GO:0006189	de novo’ IMP biosynthetic process
							0.70	GO:0009116	nucleoside metabolic process
							0.69	GO:0006541	glutamine metabolic process
-4.56	2.34E-321	BJK46_008815	phosphoribosylamine–glycine ligase	*purD*	0.65	phosphoribosylamine–glycine ligase	0.74	GO:0009113	purine nucleobase biosynthetic process
							0.69	GO:0006189	de novo’ IMP biosynthetic process
-4.54	4.33E-245	BJK46_008840	phosphoribosylformylglycinamidine synthase subunit PurL	*purL*	0.59	phosphoribosylformylglycinamidine synthase subunit PurL	0.70	GO:0006189	de novo’ IMP biosynthetic process
-4.47	3.47E-294	BJK46_008850	phosphoribosylformylglycinamidine synthase subunit PurS	*purS*	0.63	phosphoribosylformylglycinamidine synthase subunit PurS	0.71	GO:0006189	de novo’ IMP biosynthetic process
-4.42	0	BJK46_008845	phosphoribosylformylglycinamidine synthase subunit PurQ	*purQ*	0.60	phosphoribosylformylglycinamidine synthase subunit PurQ	0.70	GO:0006189	de novo’ IMP biosynthetic process
							0.69	GO:0006541	glutamine metabolic process
-4.3	0	BJK46_008855	phosphoribosylaminoimidazolesuccinocarboxamide synthase	*purC*	0.63	phosphoribosylaminoimidazole-succinocarboxamide synthase	0.71	GO:0009236	cobalamin biosynthetic process
							0.70	GO:0006189	de novo’ IMP biosynthetic process
-3.86	1.28E-235	BJK46_008860	5-(carboxyamino)imidazole ribonucleotide synthase	*purK*	0.61	N5-carboxyaminoimidazole ribonucleotide synthase	0.70	GO:0006189	de novo’ IMP biosynthetic process
-3.8	4.19E-66	BJK46_011905	peptidoglycan-binding protein						
-3.63	3.78E-181	BJK46_009875	ABC-type cobalamin Fe3+-siderophores transport system periplasmic component		0.73	hydroxamate siderophore binding lipoprotein			
-3.25	1.40E-126	BJK46_008865	5-(carboxyamino)imidazole ribonucleotide mutase	*purE*	0.57	N5-carboxyaminoimidazole ribonucleotide mutase	0.71	GO:0006189	de novo’ IMP biosynthetic process

Of the top 15 down-regulated genes, 11 genes (Down-Regulated Genes, *DRGs*), BJK46_008815 (*purD*), BJK46_008820 (*purH*), BJK46_008825 (*purN*), BJK46_008830 (*purM*), BJK46_008835 (*purF*), BJK46_008840 (*purL*), BJK46_008845 (*purQ*), BJK46_008850 (*purS*), BJK46_008855 (*purC*), BJK46_008860 (*purK*), and BJK46_008865 (*purE*), were located on a single *pur*-operon and assigned GO biological process classes predicted to be associated with ‘de novo’ IMP biosynthetic process. The GO biological process classes could not be predicted in the remaining 4 down-regulated genes.

The expression of the genes BJK46_008775 (encoding a PEP-protein phosphotransferase; PTS system enzyme I, E1), BJK46_002265 (encoding a histidine-containing phosphocarrier protein (HPr)), and BJK46_011055 (encoding a *pur*-operon repressor (PurR)) were not strongly induced or repressed by erythritol, exhibiting log_2_FC values of 0.550, 1.22, and 0.206, respectively, as shown in Additional file 1.

### Glucose supplementation restored erythritol-induced up-regulation of PTS- and cell wall-related gene transcription and growth inhibition of SP

**Fig. 3:**
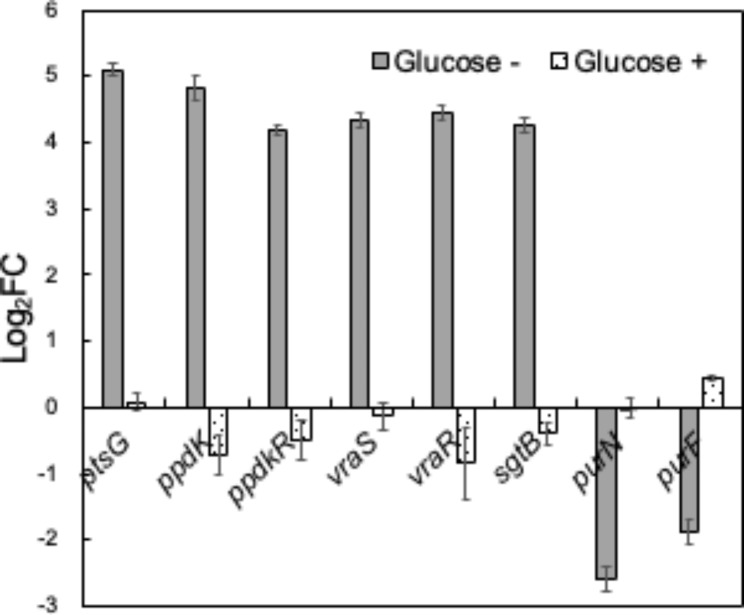
Differential gene expression analysis by RT-qPCR. The expression levels of several genes whose expression was regulated by erythritol were examined by RT-qPCR in the presence and absence of 1% glucose

**Fig. 4:**
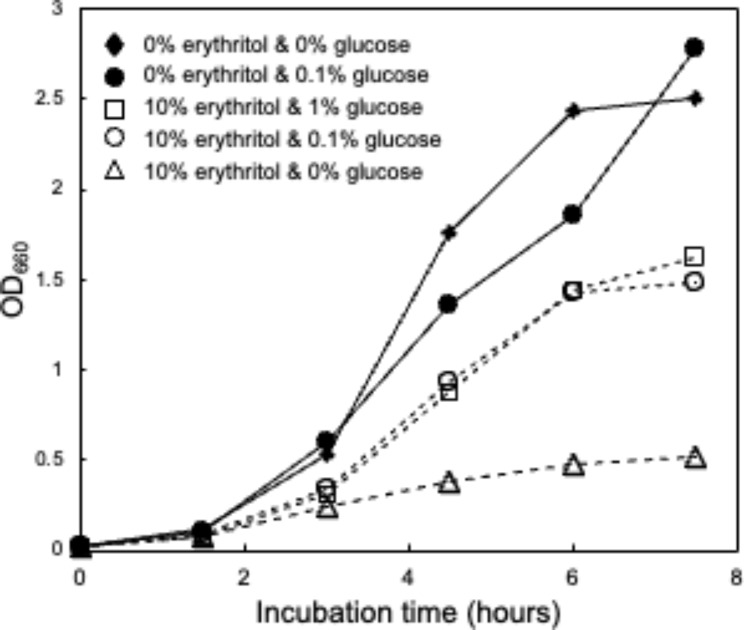
Time course of SP growth in 802 medium with erythritol and/or glucose (*n* = 3)

A quantitative reverse transcription PCR (RT-qPCR) analysis further confirmed the up-regulation of genes belonging to *URGs1* and *URGs2* in the presence of erythritol (Fig. 3). Because the PTS contributes to glucose uptake, we hypothesized that the up-regulation of PTS-related gene transcription was caused by glucose starvation in SP. Therefore, we compared the expression of PTS-related genes in SP between glucose-free and glucose-supplemented (1% [w/w]) conditions. We found that up-regulation of transcription of genes belonging to *URGs1* in response to erythritol was restored under glucose-supplemented conditions, suggesting that transcription of PTS-related genes was up-regulated in response to glucose starvation. We also found that up-regulation of transcription of genes belonging to *URGs2* in response to erythritol was restored under glucose-supplemented conditions. Moreover, supplementation of 0.1% glucose partially restored growth inhibition by erythritol (Fig. 4). Even when 1% glucose was added, the suppression of growth inhibition remained at the same level as when 0.1% glucose was added.

### Erythritol inhibited biofilm formation in SP

**Fig. 5:**
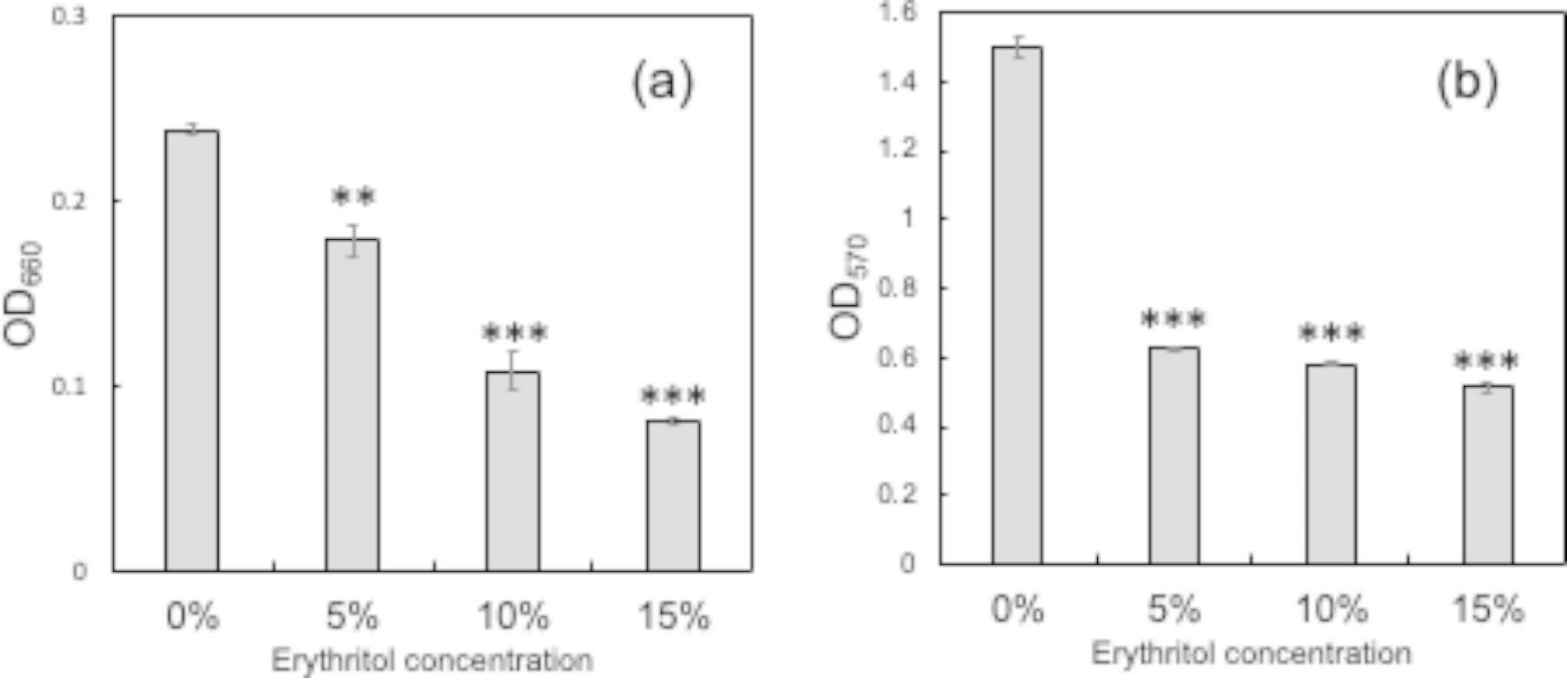
Inhibition of growth and biofilm formation of SP by erythritol. Cell growth as indicated by OD_660_ (**a**) and biofilm formation as indicated by OD_570_ (**b**) of the SP culture medium without erythritol (0%) and with erythritol (5, 10, 15%) (*n* = 8) were measured. Double asterisk indicates *p* < 0.01 and triple asterisk indicates *p* < 0.001 between the control (0%) group and each erythritol-added group

Down-regulation of genes belonging to *DRGs* was also confirmed by RT-qPCR analysis (Fig. 3). Previous reports suggested that down-regulation of *pur*-operon genes is associated with inhibition of biofilm formation in staphylococci [9]. Therefore, we hypothesized that erythritol inhibits biofilm formation in SP. Erythritol significantly inhibited the growth of SP (Fig. 5a) and the amount of biofilm formation (Fig. 5b) in a dose-dependent manner. In the condition with 5% erythritol, the biofilm inhibition effect (41.8% of OD_570_ vs. control) is notably greater than the growth inhibition effect (74.9% of OD_660_ vs. control).

## Discussion

**Fig. 6:**
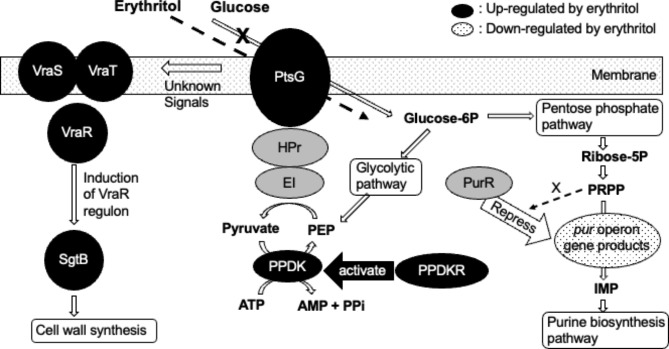
A proposed summary of the cellular responses to the addition of erythritol. Gene products of *URGs1*, *URGs2* (black circles), and *DRGs* (dotted circles) and their related gene products (gray circles) are shown. Compound names are shown in bold. Erythritol may antagonistically inhibit PTS-mediated glucose uptake by binding to the substrate binding site of PtsG, thereby exerting a growth inhibitory effect on SP. The cellular response to this may be induction of PtsG and its drivers, PPDK and PPDKR. Erythritol may respond to the VraTSR three-component system and promote cell wall synthesis by inducing SgtB, which may contribute to the suppression of some erythritol stress. Erythritol-induced glucose starvation may limit PRPP synthesis, preventing the synthesis of sufficient amounts of PRPP for binding to the PurR repressor and releasing expression of the *pur*-operon genes

Erythritol inhibited SP growth in a dose-dependent manner, as previously reported for other bacteria [4–7]. We performed a transcriptome analysis of SP with and without erythritol exposure to validate the mechanism of growth inhibition by erythritol. Among the top 15 up-regulated and top 15 down-regulated genes, we focused on the genes whose GO biological process classes were predicted for their gene products. A proposed summary of the cellular responses to the addition of erythritol is shown in Fig. 6.

The gene products of *URGs1* (*ptsG, ppdkR, ppdK*) are suggested to be involved in glucose transport. Microorganisms contain multiple PTS gene clusters with specificity for different sugars such as glucose, fructose, cellobiose, and xylose. The PTS is usually composed of one membrane-spanning protein and some soluble proteins. EI and HPr proteins are the general cytoplasmic PTS components, and in most organisms are involved in the uptake of all PTS carbohydrates. In contrast, the EIIA, EIIB, and EIIC (membrane-spanning) proteins are usually specific to one substrate or a small group of closely related carbohydrates [10]. These EII proteins are often fused to each other, and the *ptsG* gene of SP encodes the fused EIICBA protein. Bacterial PTS transports sugars up a concentration gradient with phosphorylation, and the phosphate donor is the energy-rich PEP. Pyruvate converted from PEP via PTS transportation is converted/reused by PPDK to PEP [11]. PPDK regulatory protein (PPDKR) activates PPDK via a Pi-dependent, PPi-forming phosphorolytic reaction [12]. The significant induction of *URGs1* expression by the addition of erythritol to SP cultures suggests that this may be a cellular response to erythritol-induced glucose starvation. Namely, erythritol may antagonistically inhibit PTS-mediated glucose uptake by binding to the substrate-binding site of PtsG, thereby inhibiting the growth of SP. The cellular response to this may be the induction of PtsG and its drivers, PPDK and PPDKR. This is supported by the fact that no significant induction of *URGs1* was observed under glucose-added conditions (Fig. 3). Since the growth inhibition was suppressed by adding 1/100th of the amount of glucose relative to erythritol, the affinity of erythritol to PtsG may be considerably lower than that of glucose. In the future, it will be necessary to verify whether erythritol binds to PtsG and competes with glucose uptake. In addition, PEP is well supplied by the glycolytic pathway in the presence of glucose, which may also be involved in suppressing the induction of PPDK and PPDKR by glucose.

The gene products of *URGs2* (*vraTSR* and *sgtB*) were suggested to be involved in cell wall drug resistance. It has been reported that *Staphylococcus aureus* responds to diverse classes of cell wall-inhibitory antibiotics, like methicillin, using the two-component regulatory system VraSR to up- or down-regulate a set of genes that presumably facilitates resistance to these antibiotics [13], and VraT has been reported to be a positive modulator of VraSR [14]. SgtB is known as the core cell wall stress stimulon together with PBP2 and MurZ and is also regulated by VraSR [15]. In Gram-positive bacteria, it has been reported that SgtB might participate in enhancing peptidoglycan biosynthesis and catalysing the incorporation of UDP-*N*-acetylglucosamine into peptidoglycan for cell wall elongation, thereby reducing sensitivity to antibiotics that inhibit cell wall synthesis [16]. Interestingly, no significant induction of *URGs2* was observed under glucose-added conditions (Fig. 3). The specific molecular signal responsible for *vraSTR* induction remains unknown, but some signalling from the PTS or glycolysis pathway might induce the expression of *vraTSR*. The above discussion suggests that erythritol may respond to the VraTSR three-component system and promote cell wall synthesis by inducing SgtB, which may contribute to the suppression of some erythritol stress.

Gene products of *DRGs* (*pur*-operon genes) were predicted to be involved in the ‘de novo’ IMP biosynthetic process, which leads to the purine biosynthesis pathway. It has been reported that the *pur*-operon repressor PurR of *Bacillus subtilis*, which is a structural homologue of PurR of staphylococci, regulates the transcription of all *pur*-operon genes encoding enzymes for synthesis of IMP from the starting material phosphoribosyl pyrophosphate (PRPP) [17]. Another study showed that PRPP appears to be the inducer of genes regulated by PurR, as it is the only molecule among many nucleobases, nucleosides, and nucleotides known to affect PurR-DNA binding in vitro [18]. The addition of erythritol to SP cultures significantly down-regulated *DRGs* under glucose-free conditions. This result may suggest that PRPP is not sufficiently synthesized in the presence of erythritol due to its inhibition of the PTS and is not present in sufficient amounts to bind to PurR in order to release the expression of the *pur*-operon genes. In the glucose-fed condition, PRPP metabolized from glucose may be sufficiently present to interact with PurR, and the down-regulation of the *pur*-operon genes may thus be suppressed.

It has been reported that the ‘de novo’ IMP biosynthetic process is crucial for *S. aureus* growth in minimal media but not in rich media [19], likely due to the complementary action of the purine salvage pathway in rich media [9]. This suggests that inhibition of the ‘de novo’ IMP biosynthetic process by erythritol is not directly responsible for the inhibition of SP growth in the ‘rich’ 802 medium in this study.

Increased expression of the *pur-*operon genes, which were down-regulated by erythritol, has been reported to be found during biofilm formation in Gram-positive bacteria such as *S. aureus* and *Enterococcus faecalis*, and deletion of these genes significantly impaired biofilm formation in *S. aureus* [9]. Furthermore, erythritol has been shown to be more effective than xylitol in inhibiting not only streptococcal growth but also biofilm formation [4]. These previous results suggest that erythritol might inhibit biofilm formation in SP by reducing the expression of the *pur-*operon genes. In fact, erythritol showed an inhibitory effect on biofilm formation of SP that exceeded its inhibitory effect on growth (Fig. 5). The relationship between biofilm formation and the ‘de novo’ IMP biosynthetic process has not been fully elucidated; however, it has been suggested that during biofilm formation the requirement for high amounts of purine synthesized through ‘de novo’ IMP biosynthesis might be widespread among bacteria [9].

Biofilms are complex matrices produced by microorganisms, in which cells are bound to each other and linked to biotic or abiotic surfaces [20]. Biofilm formation of staphylococci strains is well known as a factor that increases the severity of diseases and antimicrobial resistance [21]. Since *S. pseudintermedius* is the most common cause of pyoderma and otitis externa in dogs [1], the inhibitory effect of erythritol on the growth and biofilm formation of *S. pseudintermedius* raises the possibility that erythritol may be used as a therapeutic agent to treat or prevent canine infections caused by this bacterium. Indeed, there are examples of the use of erythritol in veterinary therapy, such as in the treatment of verrucous dermatitis in dairy cattle [22]. We are investigating the potential use of erythritol as a therapeutic agent to treat or prevent infections in animals caused by bacteria such as staphylococci.

## Conclusion

The aim of the present study was demonstrating the growth inhibition of *S. pseudintermedius* by erythritol and to define the changes in gene transcription signatures induced by erythritol. Erythritol inhibited *S. pseudintermedius* growth in a dose-dependent manner. Changes in the gene transcription profiles of *S. pseudintermedius* with or without erythritol were analysed by RNA-seq and RT-qPCR. We revealed that erythritol induced up-regulation of three genes (*ptsG*, *ppdK*, and *ppdkR*) that are related to the PTS. Glucose supplementation restored the up-regulation of the PTS-related genes in response to erythritol. These findings indicated that erythritol antagonistically inhibits PTS-mediated glucose uptake, thereby exerting a growth inhibitory effect on *S. pseudintermedius*. In addition, erythritol down-regulated eleven genes that are located in a single *pur*-operon and inhibited biofilm formation. Erythritol, by inhibiting the growth and biofilm formation of *S. pseudintermedius*, may be used as a therapeutic agent to treat or prevent canine infections.

## Methods

### Bacterial strain and preparation of media

SP was obtained from the Japan Collection of Microorganisms (JCM). For cultivation, 802 medium (1% hipolypepton [Fujifilm Wako, Osaka, Japan], 0.2% yeast extract [Fujifilm Wako], 0.1% MgSO_4_・7H_2_O [Fujifilm Wako], pH 7.0) with or without erythritol (B Food Science Co., Ltd., Tokyo, Japan) and/or glucose (Fujifilm Wako) were used. Xylitol, sorbitol, and maltitol were provided by B Food Science Co., Ltd.

### Growth test of SP with sugar alcohols

SP was inoculated into 802 medium and cultured at 30 °C overnight to serve as an inoculum. After dispensing 0.6 mL of 802 medium containing each sugar alcohol (erythritol, xylitol, sorbitol, and maltitol) at 0, 5, 10, or 15% (w/w) into 96 Deep Well Plates (AxyGen Scientific, CA, USA), each well was inoculated with 4 µL of each bacterium and cultured at 30 °C using an MBR-034P shaker (Taitec, Aichi, Japan) with shaking at 1,000 rpm for 16 h. Twenty microliters of these cultures were suspended in 180 µL of water in the 96-well flat-bottomed plates (4845-96 F; Watson Bio Lab, CA, USA), and turbidity (OD_660_) was measured using a microplate reader (SpectraMax M2; Molecular Devices, CA, USA) (*n* = 4). To assess the significance of differences, comparisons between the control (0%) group and each sugar-added group were performed by Tukey’s method using SPSS @ Statistics Version 26.0, with the probabilistic significance *p* < 0.05 indicating a significant difference.

### RNA-seq

A single colony of SP was inoculated into 3 mL of 802 medium in CELLSTAR® CELLreactor™ 15 ml (Greiner Bio-One, Frickenhausen, Germany)　and cultured at 30 °C for 15 h with shaking at 210 rpm using a BR-23FP MR shaker (Taitec) until OD_660_ = 3.4. For RNA preparation of bacteria cultured in 802 medium with or without 5% (w/w) erythritol, 60 or 30 µL of the above seed culture was inoculated into 3 mL of 802 medium, respectively, and incubated at 30 °C until OD_660_ = 0.8–1.0 for three independent cultures under each condition. After cooling 3 mL of the culture medium on ice, the cells were collected by centrifugation at 5,000 rpm for 5 min using the centrifuge MX-107 (Tomy Seiko, Tokyo, Japan), and 800 µl of RNA later® (Thermo Fisher Scientific, MA, USA) was then added to cells for RNA extraction. Total RNA was extracted using the RNeasy® Mini Kit (Qiagen, Hilden, Germany) according to the manufacturer’s instructions. The total RNA of samples were sent to Seibutsu Giken Inc. (Kanagawa, Japan) for next generation sequencing (NGS). Briefly, the concentrations of the RNA samples were measured using the Quantus Fluorometer and Quanti Fluor RNA system (Promega, Madison, USA). Quality check of the RNA was performed with 5200 Fragment Analyzer System and Agilent HS RNA Kit (Agilent Technologies, CA, USA). After removing rRNA in samples by riboPool™ (siTOOLs Biotech, Planegg, Germany), the NGS library was prepared by MGIEasy RNA Directional Library Prep Set (MGI Tech, Shenzhen, China) according to the manufacturer’s protocol. The concentrations of the library were measured with Qubit 3.0 Fluorometer and dsDNA HS Assay Kit (Thermo Fisher Scientific). Quality check of the library was performed with Fragment Analyzer and dsDNA 915 Reagent Kit (Advanced Analytical Technologies, Iowa, USA). A circular DNA was made from the library by MGIEasy Circularization Kit (MGI Tech). DNA Nanoball (DNB) was made with DNBSEQG 400 RS High throughput Sequencing Kit (MGI Tech) according to the manufacturer’s protocol. Sequencing was performed with DNBSEQ-G400 for at least 2 × 100 bp read. The adapters and primers from the delivered FASTQ files were removed using cutadapt (ver. 1.9.1). Short read sequences under 20 and low-quality score reads (under 40) were removed with a sickle (ver. 1.33). Bowtie2 (ver. 2.4.1) was used to read the alignment and mapping of the GCF_001792775.2 reference genomes. After reading and writing the alignment data in the SAM and BAM formats with the SAMtools, the data were sorted and indexed. The read counts per gene were obtained using the featureCounts (ver. 2.0.0). The relative expression level of each gene was normalized by the RPKM (Reads per Kilobase Million) and Transcripts Per Million methods. After normalization using the DEGES normalization method implemented in TCC (ver. 1.26.0), we performed a differential gene expression analysis using edgeR (ver. 3.28.1). The PCA plot and the volcano plot were created to visualize the RNA-seq results using the Nucleic Acid SeQuence Analysis Resource (NASQAR; https://nasqar.abudhabi.nyu.edu/#). The top 15 up-regulated and top 15 down-regulated genes in response to erythritol obtained by RNA-seq analysis were annotated using Protein ANNotation with Z-scoRE (PANNZER2; http://ekhidna2.biocenter.helsinki.fi/sanspanz/) to predict functional descriptions and Gene Ontology (GO) classes of the biological process.

### Analysis of RT-qPCR

RT-qPCR analysis was performed as follows. Total RNA of the cells cultured in 802 medium was extracted as above. Total RNA of the cells cultured in 802 medium containing 1% (w/w) glucose was prepared in the same manner as that described above (*n* = 3). Complementary DNA synthesis was performed using the Transcriptor First Strand cDNA Synthesis Kit (Roche Diagnostics, Ottweiler, Germany) with random primers. The primer sets used for RT-qPCR had 100% identity with the gene sequences of SP (GenBank: MLGE00000000.2) and the primer sets were selected using Primer-BLAST (Table 2). An RT-qPCR reaction was performed using these primer sets and TB Green® Fast qPCR Mix (Takara Bio, Shiga, Japan) with 45 cycles at 95 °C for 5 s and 55 °C for 60 s on a Thermal Cycler Dice® Real-Time System III (Takara Bio). The absolute copy numbers of genes listed in Table 2 in the cDNA samples were determined from the corresponding standard curves, using DNA samples of known concentration and the CT values. Relative gene expression levels were assessed using the *recA* gene as a reference gene [23].

**Table 2:** Sequences of PCR primers used for qPCR.

Primer set	Sequence (5’–3’)	PCR product length (bp)
purN	ACTCGCGCATATCGAAGTCA TACACCTTCCTGACGCAACC	195
ptsG	GCATTGGTGGCTCGTGATTC AAGGCAATTCGCTTGCAGTC	192
ppdK	TCATTCCACCGTGTGTCGTT	153
	CGGCGACGGGTAAAATTGTG	
ppdkR	CTCACCGCAAGCCCTGAATA	157
	TGACTGTCGCATTGAGCTGT	
vraS	CAATACGTTACGACATGCTG	169
	ACCAATCTCTATTGCACGTT	
vraR	CGTTAGATGCCGGTGTCGAT	177
	TCTCGCGCTCAGTCAACAAT	
sgtB	CGCTCAATCCGTTTAGCGAC	150
	ACGTGCGATTTTTGCATCGG	
purN	ACTCGCGCATATCGAAGTCA TACACCTTCCTGACGCAACC	195
purF	ATCCGAACGCCATTGGTCAT TTGGAAAATGCCGCCTGTTG	176
recA	GGGCCGAGCTCTGAAATTCT CTCCTTCGCGTGAAATCCCT	185

### Time course of SP growth with erythritol and/or glucose

SP was inoculated into 802 medium containing 0.1% (w/w) glucose and cultured at 37 °C for 2 h to serve as an inoculum (OD_660_ = 1.47). After dispensing 0.6 mL of 802 medium with 0% erythritol and 0% glucose; 0% erythritol and 0.1% glucose; 10% erythritol and 1% glucose; 10% erythritol and 0.1% glucose; and 10% erythritol or 0% glucose into 96 Deep Well Plates, each well was inoculated with 20 µL of the SP broth and cultured at 37 °C with shaking at 1,000 rpm. After 1.5, 3, 4.5, 6, and 7.5 h of incubation, turbidity (OD_660_) was measured as shown above (*n* = 4).

### Biofilm adhesion assay

Biofilm adhesion was examined using a crystal violet staining assay based on a previously reported method [24]. Briefly, SP was inoculated into 2 × 802 medium and cultured at 37 °C with shaking at 210 rpm until OD_660_ = 1.0. A 100-µL aliquot of this culture was added to 100 µL of erythritol solution containing 0, 5, 10, and 15% (w/w)　erythritol dispensed into 96-well cell culture plates (197-96CPS; Watson Bio Lab). In the case of glucose-containing cultures, glucose was added at a concentration of 1.0% (w/w). After incubation at 37 °C for 24 h without shaking to allow biofilm formation, OD_660_ was measured to use as an indicator of bacterial growth. The contents of the wells were then discarded with a simple decant and each well was washed by gently immersing in 500 mL of water twice to remove non-adherent cells while carefully maintaining the integrity of the formed biofilms. Biofilms were heat-fixed at 60 °C for 60 min. Adherent cells were stained with 150 µL of 0.2% (w/v) crystal violet for 15 min at room temperature and then dried at 30 °C. After resolubilization with 95% ethanol, OD_570_ was measured and used as an indicator of the amount of biofilm formation. In the statistical analyses, comparisons between the control (0%) group and each erythritol-added group were performed using Tukey’s method with SPSS @ Statistics Version 26.0. The sample size was *n* = 8.

### Electronic supplementary material

Below is the link to the electronic supplementary material.

Supplementary Material 1

## Data Availability

The datasets of RNA-seq generated during the current study are available in Gene Expression Omnibus with the accession number of GSE227665.

## List of Abbreviations

SP: *Staphylococcus pseudintermedius* JCM 17571
MRSP: Methicillin-resistant *Staphylococcus pseudintermedius*
PTS: Phosphoenolpyruvate-dependent sugar phosphotransferase system
PEP: Phosphoenolpyruvate
PRPP: Phosphoribosyl pyrophosphate
RT-qPCR: Quantitative reverse transcription PCR
FC: Fold change
*URGs*: Up-Regulated Genes
*DRGs*: Down-Regulated Genes
JCM: Japan Collection of Microorganisms
PCA: Principal Component Analysis
GO: Gene Ontology

## References

[1] Duim B, Verstappen KMHW, Kalupahana RS, Ranathunga L, Fluit AC, Wagenaar JA (2018). Methicillin-resistant *Staphylococcus pseudintermedius* among dogs in the description of novel SCCmec variants. Vet Microbiol.

[2] Priyantha MAR (2022). An overview of human infections caused by *Staphylococcus pseudintermedius*: a zoonotic risk of the oldest friend. Sri Lankan J Infect Dis.

[3] European Food Safety Authority (2015). Scientific opinion on the safety of the proposed extension of use of erythritol (E 968) as a food additive. EFSA J.

[4] Decock P, Mäkinen KK, Honkala E, Saag M, Kennepohl E, Eapen AK. Erythritol is more effective than xylitol and sorbitol in managing oral health endpoints. Int J Dent. 2016; 2016:9868421. 10.1155/2016/9868421 PMC5011233 27635141

[5] Hashino E, Kuboniwa M, Alghamdi SA, Yamaguchi M, Yamamoto R, Cho H, Amano A (2013). Erythritol alters microstructure and metabolomic profiles of biofilm composed of *Streptococcus gordonii* and *Porphyromonas gingivalis*. Mol Oral Microbiol.

[6] Fujii T, Inoue S, Kawai Y, Tochio T, Takahashi K (2022). Suppression of axillary odor and control of axillary bacterial flora by erythritol. J Cosmet Dermatol.

[7] Fujii T, Tochio T, Endo A. Ribotype-dependent growth inhibition and promotion by erythritol in *Cutibacterium acnes*. J Cosmet Dermatol. 2022; Apr 1. 10.1111/jocd.14958. 10.1111/jocd.14958 35364613

[8] Miyasawa-Horia H, Aizawa S, Takahashia N (2006). Difference in the xylitol sensitivity of acid production among *Streptococcus mutans* strains and the biochemical mechanism. Oral Microbiol Immunol.

[9] Gélinas M, Museau L, Milot A, Beauregard PB (2021). The *de novo* purine biosynthesis pathway is the only commonly regulated cellular pathway during biofilm formation in TSB-based medium in *Staphylococcus aureus* and *Enterococcus faecalis*. Microbiol Spectr.

[10] Deutscher J, Aké FMD, Derkaoui M, Zébré AC, Cao TN, Bouraoui H, Kentache T, Mokhtari A, Milohanic E, Joyet P (2014). The bacterial phosphoenolpyruvate:carbohydrate phosphotransferase system: regulation by protein phosphorylation and phosphorylation-dependent protein-protein interactions. Microbiol Mol Biol Rev.

[11] Cui J, Maloney MI, Olson DG, Lynd LR (2020). Conversion of phosphoenolpyruvate to pyruvate in *Thermoanaerobacterium saccharolyticum*. Metab Eng Commun.

[12] Chastain CJ, Xu W, Parsley K, Sarath G, Hibberd JM, Chollet R (2008). The pyruvate, orthophosphate dikinase regulatory proteins of Arabidopsis possess a novel, unprecedented Ser/Thr protein kinase primary structure. Plant J.

[13] Kuroda M, Kuroda H, Oshima T, Takeuchi F, Mori H, Hiramatsu K (2003). Two-component system VraSR positively modulates the regulation of cell-wall biosynthesis pathway in *Staphylococcus aureus*. Mol Microbiol.

[14] Boyle-Vavra S, Yin S, Jo DS, Montgomery CP, Daum RS (2013). VraT/YvqF is required for methicillin resistance and activation of the VraSR regulon in *Staphylococcus aureus*. Antimicrob Agents Chemother.

[15] Wu S, Lin K, Liu Y, Zhang H, Lei L (2020). Twocomponent signaling pathways modulate drug resistance of *Staphylococcus aureus* (Review). Biomed Rep.

[16] Qamar A, Golemi-Kotra D (2012). Dual roles of FmtA in *Staphylococcus aureus* cell wall biosynthesis and autolysis. Antimicrob Agents Chemother.

[17] Sinha SC, Krahn J, Shin BS, Tomchick DR, Zalkin H, Smith JL (2003). The purine repressor of *Bacillus subtilis*: a novel combination of domains adapted for transcription regulation. J Bacteriol.

[18] Weng M, Nagy PL, Zalkin H (1995). Identification of the *Bacillus subtilis pur* operon repressor. Proc Natl Acad Sci USA.

[19] Sause WE, Balasubramanian D, Irnov I, Copin R, Sullivan MJ, Sommerfield A, Chan R, Dhabaria A, Askenazi M, Ueberheide B, Shopsin B, Bakel HV, Torres VJ (2019). The purine biosynthesis regulator PurR moonlights as a virulence regulator in *Staphylococcus aureus*. Proc Natl Acad Sci USA.

[20] Yin W, Wang Y, Liu L, He J (2019). Biofilms: the microbial protective clothing in extreme environments. Int J Mol Sci.

[21] Arima S, Ochi H, Mitsuhashi M, Kibe R, Takahashi K, Kataoka Y (2018). *Staphylococcus pseudintermedius* biofilms secrete factors that induce inflammatory reactions *in vitro*. Lett Appl Microbiol.

[22] Sasaki N, Takakuwa J, Nishii S, Ishii M, Kadohira M, Naitou Y, Manabe H, Yamada H (2009). Effect of erythritol on verrucous dermatitis in dairy cattle. J Jpn Vet Med Assoc.

[23] Crawford EC, Singh A, Metcalf D, Gibson TWG, Weese SJ (2014). Identification of appropriate reference genes for qPCR studies in *Staphylococcus pseudintermedius* and preliminary assessment of *icaA* gene expression in biofilm-embedded bacteria. BMC Res Notes.

[24] Stepanović S, Vuković D, Hola V, Bonaventura GD, Djukić S, Cirković I, Ruzicka F (2007). Quantification of biofilm in microtiter plates: overview of testing conditions and practical recommendations for assessment of biofilm production by staphylococci. APMIS.

