# Supplementary material for: Erythritol alters gene transcriptome signatures, cell growth, and biofilm formation in Staphylococcus pseudintermedius
Source: BMC Vet Res. 2023 Sep 7;19:146. doi: 10.1186/s12917-023-03711-3 (PMC10483758; doi:10.1186/s12917-023-03711-3)
Supplement: Supplementary file 1 — Supplementary Material 1 [file 12917_2023_3711_MOESM1_ESM.docx]

**Additional file 1** Number of clean reads for the control sample (C1, C2, C3) and for the erythritol-treated sample (E1, E2, E3), respectively.

| **gene_id** | **C1** | **C2** | **C3** | **E1** | **E2** | **E3** | **logFC_E/C** | **p.value** |
| --- | --- | --- | --- | --- | --- | --- | --- | --- |
| BJK46_000005 | 28 | 21 | 25 | 136 | 361 | 192 | 1.618338981 | 5.98E-18 |
| BJK46_000010 | 24 | 35 | 24 | 36 | 37 | 27 | -1.64727 | 0.56326848 |
| BJK46_000130 | 18607 | 19784 | 18596 | 13848 | 13028 | 14378 | -0.2235682 | 1.01E-06 |
| BJK46_000135 | 21676 | 23021 | 20358 | 18565 | 18317 | 21935 | 0.119049688 | 0.08332342 |
| BJK46_000140 | 13493 | 14200 | 16044 | 11438 | 12487 | 11803 | -0.0744022 | 3.85E-05 |
| BJK46_000145 | 1730 | 1626 | 2048 | 2396 | 2818 | 2360 | 0.506844844 | 1.03E-05 |
| BJK46_000155 | 3749 | 3822 | 4249 | 4419 | 4570 | 4655 | 0.261155837 | 0.05953896 |
| BJK46_000160 | 1525 | 1435 | 1663 | 3862 | 4267 | 4295 | 1.451327409 | 8.01E-54 |
| BJK46_000165 | 920 | 834 | 1141 | 3044 | 3138 | 3161 | 1.69888362 | 1.32E-53 |
| BJK46_000170 | 138 | 137 | 131 | 205 | 304 | 289 | -0.01272655 | 0.0001395 |
| BJK46_000180 | 464 | 510 | 565 | 537 | 1117 | 682 | 0.141555774 | 0.01866787 |
| BJK46_000185 | 1093 | 1078 | 1237 | 1358 | 3000 | 1801 | 0.743570135 | 4.72E-05 |
| BJK46_000190 | 842 | 958 | 905 | 2540 | 2787 | 2459 | 1.497556037 | 6.15E-49 |
| BJK46_000195 | 1750 | 1817 | 1882 | 5496 | 5635 | 5252 | 1.621730825 | 3.29E-80 |
| BJK46_000200 | 1609 | 1574 | 1805 | 4732 | 5139 | 4594 | 1.56542758 | 1.43E-72 |
| BJK46_000205 | 5050 | 4999 | 5963 | 10705 | 11829 | 9907 | 1.13097467 | 9.16E-30 |
| BJK46_000210 | 15244 | 15207 | 12463 | 74078 | 91290 | 106954 | 2.979039184 | 2.58E-59 |
| BJK46_000215 | 4275 | 4623 | 3431 | 5918 | 6976 | 8551 | 0.860291681 | 3.33E-07 |
| BJK46_000220 | 27381 | 28809 | 32458 | 16145 | 17741 | 17004 | -0.52263987 | 1.12E-18 |
| BJK46_000225 | 212 | 252 | 287 | 213 | 258 | 256 | -0.87270163 | 0.62313615 |
| BJK46_000230 | 5417 | 6391 | 7684 | 6835 | 6891 | 6670 | 0.168545082 | 0.88646048 |
| BJK46_000235 | 1977 | 2147 | 3189 | 2645 | 3157 | 2456 | 0.224629289 | 0.32727583 |
| BJK46_000240 | 1710 | 1886 | 2003 | 2338 | 3058 | 2396 | 0.48802691 | 1.37E-05 |
| BJK46_000245 | 7273 | 7719 | 8593 | 3684 | 4070 | 3749 | -0.9650845 | 1.53E-53 |
| BJK46_000250 | 3094 | 3419 | 2481 | 1575 | 1446 | 1782 | -0.87859258 | 7.92E-12 |
| BJK46_000255 | 755 | 855 | 791 | 592 | 788 | 804 | -0.44843639 | 0.22246648 |
| BJK46_000260 | 665 | 756 | 710 | 1413 | 1695 | 1390 | 0.906817757 | 1.30E-13 |
| BJK46_000265 | 1244 | 1372 | 1437 | 2671 | 3485 | 2613 | 1.115568425 | 1.08E-25 |
| BJK46_000270 | 3 | 3 | 7 | 37 | 69 | 39 | 1.075396217 | 1.62E-14 |
| BJK46_000275 | 131 | 97 | 90 | 358 | 990 | 551 | 1.638241785 | 4.77E-14 |
| BJK46_000280 | 231 | 218 | 229 | 557 | 1314 | 776 | 1.312808782 | 6.69E-13 |
| BJK46_000285 | 156 | 144 | 155 | 342 | 592 | 418 | 0.725979454 | 2.19E-10 |
| BJK46_000290 | 188 | 205 | 198 | 1278 | 1536 | 1256 | 2.29783248 | 1.85E-56 |
| BJK46_000295 | 1751 | 1480 | 2141 | 39594 | 42040 | 37786 | 4.618671717 | 3.41E-230 |
| BJK46_000300 | 4587 | 3762 | 4354 | 76494 | 85290 | 74295 | 4.47413709 | 2.28E-202 |
| BJK46_000305 | 533 | 601 | 686 | 502 | 866 | 642 | -0.27215662 | 0.70933745 |
| BJK46_000310 | 349 | 386 | 435 | 1125 | 1272 | 1378 | 1.351254511 | 1.15E-24 |
| BJK46_000315 | 5952 | 6519 | 6203 | 15236 | 16727 | 19726 | 1.631343222 | 2.10E-39 |
| BJK46_000320 | 7304 | 7963 | 8925 | 19329 | 20847 | 20803 | 1.547462254 | 3.26E-44 |
| BJK46_000325 | 3093 | 3447 | 4159 | 11291 | 11722 | 10492 | 1.738498848 | 2.14E-75 |
| BJK46_000330 | 649 | 775 | 863 | 3338 | 3957 | 3277 | 2.199430699 | 3.55E-108 |
| BJK46_000335 | 445 | 461 | 523 | 1282 | 1703 | 1364 | 1.3357511 | 4.07E-25 |
| BJK46_000340 | 470 | 512 | 612 | 1516 | 1797 | 1493 | 1.380254333 | 1.71E-27 |
| BJK46_000345 | 2814 | 3036 | 3130 | 3062 | 3569 | 3460 | 0.200814869 | 0.18775419 |
| BJK46_000350 | 601 | 686 | 558 | 883 | 1158 | 1131 | 0.500715503 | 2.72E-05 |
| BJK46_000355 | 582 | 696 | 682 | 1193 | 1787 | 1371 | 0.940226969 | 2.17E-12 |
| BJK46_000360 | 230 | 248 | 280 | 692 | 934 | 726 | 1.075037907 | 8.56E-17 |
| BJK46_000365 | 369 | 324 | 381 | 662 | 786 | 650 | 0.469873174 | 1.05E-06 |
| BJK46_000370 | 925 | 804 | 1033 | 1513 | 1817 | 1422 | 0.678243542 | 2.21E-07 |
| BJK46_000375 | 1078 | 1108 | 1328 | 1692 | 2082 | 1581 | 0.560844535 | 5.51E-06 |
| BJK46_000380 | 679 | 715 | 781 | 282 | 378 | 328 | -1.64990376 | 2.41E-11 |
| BJK46_000385 | 21 | 26 | 21 | 49 | 73 | 37 | -0.698528 | 0.00065565 |
| BJK46_000390 | 2773 | 3125 | 2160 | 1942 | 2193 | 2725 | -0.20641947 | 0.04690723 |
| BJK46_000395 | 2243 | 2551 | 2033 | 3064 | 3031 | 4026 | 0.588892114 | 0.000159 |
| BJK46_000400 | 1104 | 1323 | 895 | 1320 | 1172 | 1762 | 0.25986449 | 0.13199649 |
| BJK46_000405 | 786 | 883 | 935 | 2000 | 2323 | 2034 | 1.225438506 | 9.73E-30 |
| BJK46_000410 | 1890 | 2106 | 2250 | 4381 | 4853 | 4235 | 1.142031332 | 5.98E-41 |
| BJK46_000415 | 1160 | 1331 | 1279 | 2769 | 2838 | 2793 | 1.165899091 | 6.84E-33 |
| BJK46_000420 | 1550 | 1778 | 1896 | 3718 | 3696 | 3618 | 1.107659189 | 1.48E-33 |
| BJK46_000425 | 1471 | 1597 | 1614 | 3176 | 3089 | 3084 | 1.017920737 | 4.68E-26 |
| BJK46_000430 | 1529 | 1570 | 1835 | 3429 | 3298 | 3213 | 1.036046202 | 2.05E-26 |
| BJK46_000435 | 1684 | 1795 | 1899 | 4038 | 3755 | 3567 | 1.104014968 | 6.99E-29 |
| BJK46_000440 | 1688 | 1831 | 1917 | 4467 | 4015 | 4339 | 1.266061867 | 5.72E-37 |
| BJK46_000445 | 3385 | 3840 | 3842 | 9189 | 7885 | 9081 | 1.310908441 | 1.32E-34 |
| BJK46_000450 | 1716 | 1964 | 2166 | 3778 | 3879 | 3573 | 0.974127783 | 3.83E-26 |
| BJK46_000455 | 1128 | 1383 | 1615 | 2778 | 3212 | 2574 | 1.074710809 | 7.85E-22 |
| BJK46_000460 | 865 | 1086 | 1150 | 2466 | 2612 | 2356 | 1.252343862 | 5.81E-33 |
| BJK46_000465 | 296 | 301 | 229 | 2325 | 2434 | 3071 | 2.988955955 | 7.13E-75 |
| BJK46_000470 | 48 | 49 | 44 | 148 | 387 | 193 | 0.954126746 | 1.33E-11 |
| BJK46_000475 | 160 | 157 | 144 | 397 | 908 | 552 | 1.191636469 | 1.35E-12 |
| BJK46_000480 | 1449 | 1610 | 1795 | 1498 | 2234 | 1541 | 0.091869583 | 0.68558297 |
| BJK46_000485 | 5044 | 5735 | 7179 | 6712 | 7232 | 6779 | 0.305716242 | 0.07580324 |
| BJK46_000490 | 392 | 447 | 404 | 265 | 531 | 335 | -0.80620769 | 0.35533375 |
| BJK46_000495 | 682 | 791 | 737 | 234 | 481 | 301 | -1.6865134 | 1.50E-07 |
| BJK46_000500 | 1718 | 2403 | 1636 | 1331 | 1093 | 1428 | -0.59037898 | 0.00010122 |
| BJK46_000505 | 26 | 24 | 17 | 65 | 187 | 78 | 0.441199598 | 2.10E-08 |
| BJK46_000510 | 1610 | 1900 | 1728 | 2988 | 3154 | 3610 | 0.917517235 | 1.71E-16 |
| BJK46_000515 | 144 | 174 | 143 | 447 | 496 | 594 | 0.963404075 | 4.37E-14 |
| BJK46_000520 | 37 | 56 | 34 | 69 | 133 | 91 | -0.36246083 | 0.00030741 |
| BJK46_000525 | 12 | 4 | 6 | 24 | 96 | 41 | 0.502196516 | 3.59E-08 |
| BJK46_000530 | 73 | 64 | 58 | 215 | 484 | 280 | 1.128352125 | 1.88E-13 |
| BJK46_000535 | 36 | 43 | 26 | 57 | 107 | 78 | -0.46398896 | 0.00041602 |
| BJK46_000540 | 1711 | 1857 | 2205 | 1364 | 1843 | 1396 | -0.33731492 | 0.00036732 |
| BJK46_000545 | 9464 | 10689 | 12036 | 4544 | 5078 | 4826 | -1.04815014 | 2.72E-55 |
| BJK46_000550 | 7924 | 9274 | 9283 | 5122 | 5237 | 5774 | -0.61788048 | 9.47E-20 |
| BJK46_000555 | 74 | 49 | 77 | 71 | 214 | 124 | -0.43568891 | 0.00683302 |
| BJK46_000560 | 109 | 81 | 79 | 124 | 316 | 171 | -0.07437902 | 0.00084165 |
| BJK46_000565 | 75 | 73 | 52 | 199 | 496 | 264 | 1.045685601 | 5.08E-11 |
| BJK46_000570 | 90 | 106 | 68 | 261 | 712 | 371 | 1.273657134 | 1.35E-11 |
| BJK46_000575 | 70 | 61 | 44 | 205 | 579 | 322 | 1.425218194 | 3.30E-13 |
| BJK46_000580 | 30 | 33 | 26 | 74 | 185 | 109 | 0.35635416 | 9.30E-09 |
| BJK46_000585 | 30 | 40 | 32 | 88 | 181 | 99 | 0.232921986 | 2.48E-08 |
| BJK46_000590 | 1700 | 2250 | 1761 | 1951 | 1859 | 2392 | 0.133968135 | 0.66082117 |
| BJK46_000595 | 329 | 354 | 349 | 291 | 486 | 359 | -0.54170202 | 0.77328715 |
| BJK46_000600 | 8573 | 10180 | 7125 | 4419 | 4635 | 6413 | -0.65964458 | 2.14E-07 |
| BJK46_000605 | 34 | 41 | 33 | 72 | 192 | 90 | 0.029075367 | 3.49E-06 |
| BJK46_000610 | 229 | 222 | 238 | 502 | 1203 | 715 | 1.140610691 | 5.05E-11 |
| BJK46_000615 | 110 | 99 | 88 | 429 | 1195 | 588 | 1.952909537 | 7.13E-18 |
| BJK46_000620 | 65 | 71 | 68 | 248 | 698 | 352 | 1.488925181 | 4.44E-15 |
| BJK46_000625 | 49 | 50 | 42 | 160 | 404 | 230 | 1.124441571 | 2.21E-13 |
| BJK46_000630 | 5 | 2 | 1 | 11 | 24 | 11 | -0.29476454 | 4.04E-05 |
| BJK46_000635 | 195 | 280 | 171 | 175 | 163 | 214 | -1.14743068 | 0.26619837 |
| BJK46_000640 | 1328 | 1331 | 1323 | 1203 | 1597 | 1430 | 0.017762024 | 0.90458535 |
| BJK46_000645 | 200 | 212 | 158 | 286 | 287 | 350 | -0.142161 | 0.00792005 |
| BJK46_000650 | 1548 | 1695 | 1314 | 1521 | 1681 | 1790 | 0.116804973 | 0.65148382 |
| BJK46_000655 | 12 | 4 | 9 | 16 | 41 | 25 | -0.62569592 | 0.00025251 |
| BJK46_000660 | 104 | 107 | 99 | 217 | 342 | 231 | 0.278078163 | 3.31E-07 |
| BJK46_000665 | 61 | 70 | 59 | 140 | 341 | 170 | 0.432234926 | 1.20E-07 |
| BJK46_000670 | 94 | 94 | 120 | 128 | 233 | 145 | -0.4865997 | 0.01613118 |
| BJK46_000675 | 3216 | 3978 | 3746 | 5019 | 5069 | 5938 | 0.606609239 | 2.30E-07 |
| BJK46_000680 | 10854 | 12798 | 12643 | 13724 | 14169 | 15586 | 0.477614124 | 0.03374058 |
| BJK46_000685 | 25131 | 28165 | 34080 | 20203 | 19357 | 16541 | -0.35395797 | 2.45E-09 |
| BJK46_000690 | 28599 | 31462 | 37195 | 20775 | 21729 | 18733 | -0.37298938 | 9.06E-12 |
| BJK46_000695 | 11674 | 12420 | 14249 | 5334 | 5835 | 5104 | -1.10590053 | 4.10E-58 |
| BJK46_000700 | 5793 | 6535 | 7201 | 2706 | 2999 | 2511 | -1.19512918 | 3.57E-62 |
| BJK46_000705 | 17580 | 20355 | 14878 | 9065 | 9860 | 13395 | -0.49743364 | 1.06E-06 |
| BJK46_000710 | 3336 | 3495 | 3793 | 5036 | 5460 | 5171 | 0.61473285 | 1.10E-11 |
| BJK46_000715 | 8470 | 10335 | 10892 | 9082 | 9670 | 9988 | 0.117595278 | 0.21871236 |
| BJK46_000720 | 1208 | 1156 | 1476 | 972 | 1617 | 1092 | -0.18183752 | 0.40693948 |
| BJK46_000725 | 10595 | 11453 | 13086 | 5835 | 5557 | 5818 | -0.89938261 | 1.38E-36 |
| BJK46_000730 | 123 | 101 | 102 | 236 | 554 | 333 | 0.750288589 | 7.47E-09 |
| BJK46_000735 | 75 | 53 | 56 | 122 | 276 | 156 | 0.233669899 | 2.56E-06 |
| BJK46_000740 | 271 | 304 | 311 | 451 | 1020 | 578 | 0.567886997 | 7.48E-06 |
| BJK46_000745 | 2607 | 2784 | 2940 | 2796 | 3131 | 2789 | 0.093619609 | 0.98850153 |
| BJK46_000750 | 67376 | 77167 | 80747 | 125039 | 118435 | 122031 | 1.132270787 | 3.86E-09 |
| BJK46_000755 | 88632 | 96146 | 107147 | 112138 | 110322 | 105961 | 0.611513453 | 0.2915589 |
| BJK46_000760 | 2118 | 2249 | 2401 | 35805 | 37330 | 42669 | 4.24392698 | 5.40E-246 |
| BJK46_000765 | 199 | 193 | 196 | 326 | 712 | 420 | 0.498904516 | 2.68E-06 |
| BJK46_000770 | 787 | 849 | 734 | 1144 | 1378 | 1617 | 0.629342771 | 5.76E-06 |
| BJK46_000775 | 614 | 625 | 710 | 358 | 829 | 472 | -0.74299626 | 0.19447303 |
| BJK46_000780 | 425 | 395 | 517 | 384 | 811 | 524 | -0.19639621 | 0.22976115 |
| BJK46_000785 | 555 | 523 | 639 | 1101 | 1368 | 1100 | 0.787919274 | 7.74E-11 |
| BJK46_000790 | 904 | 1030 | 984 | 2328 | 2450 | 2475 | 1.288232055 | 4.74E-35 |
| BJK46_000795 | 2401 | 2651 | 2992 | 1863 | 2529 | 1855 | -0.35267062 | 1.77E-05 |
| BJK46_000800 | 1275 | 1555 | 1236 | 577 | 697 | 772 | -1.19078077 | 8.66E-11 |
| BJK46_000805 | 128 | 178 | 193 | 182 | 241 | 225 | -0.57902956 | 0.1684941 |
| BJK46_000810 | 26987 | 29000 | 33338 | 13000 | 14750 | 14508 | -0.8095254 | 5.46E-30 |
| BJK46_000815 | 62578 | 67794 | 80076 | 30037 | 32159 | 31953 | -0.8003442 | 5.05E-30 |
| BJK46_000820 | 34776 | 38185 | 44502 | 16844 | 17215 | 17085 | -0.9029889 | 2.06E-33 |
| BJK46_000825 | 26194 | 30434 | 40027 | 10023 | 10639 | 10064 | -1.37645762 | 9.33E-47 |
| BJK46_000830 | 2550 | 2758 | 2918 | 2926 | 3031 | 2767 | 0.114537259 | 0.77390002 |
| BJK46_000835 | 2027 | 2057 | 2262 | 2355 | 2653 | 2310 | 0.225027912 | 0.09941785 |
| BJK46_000840 | 296 | 334 | 336 | 400 | 463 | 397 | -0.25383614 | 0.09365025 |
| BJK46_000845 | 237 | 226 | 165 | 295 | 421 | 403 | 0.060347046 | 0.0018683 |
| BJK46_000850 | 265 | 312 | 223 | 542 | 589 | 654 | 0.565597232 | 2.93E-07 |
| BJK46_000855 | 8568 | 9381 | 10516 | 7642 | 7636 | 7134 | -0.21079604 | 5.88E-07 |
| BJK46_000860 | 2544 | 2868 | 3427 | 2598 | 2717 | 2639 | -0.11358917 | 0.01676228 |
| BJK46_000865 | 2163 | 2542 | 2606 | 3651 | 3764 | 3478 | 0.61024952 | 2.36E-10 |
| BJK46_000870 | 1180 | 1277 | 1374 | 2150 | 2560 | 2167 | 0.836985405 | 6.97E-17 |
| BJK46_000875 | 235 | 244 | 226 | 345 | 984 | 526 | 0.620272978 | 1.02E-05 |
| BJK46_000880 | 286 | 280 | 301 | 418 | 1085 | 573 | 0.579756807 | 2.14E-05 |
| BJK46_000885 | 439 | 428 | 408 | 570 | 1517 | 766 | 0.634697603 | 8.08E-05 |
| BJK46_000890 | 443 | 496 | 521 | 709 | 1664 | 984 | 0.785542915 | 1.53E-06 |
| BJK46_000895 | 3897 | 4295 | 4432 | 4341 | 4639 | 4537 | 0.154086072 | 0.61956781 |
| BJK46_000900 | 3862 | 3948 | 4342 | 4691 | 5140 | 4744 | 0.317916706 | 0.0067419 |
| BJK46_000905 | 2999 | 3462 | 3630 | 4278 | 4775 | 4273 | 0.450572857 | 5.00E-06 |
| BJK46_000910 | 2788 | 3150 | 3007 | 4984 | 5036 | 5974 | 0.883743594 | 4.34E-16 |
| BJK46_000915 | 11835 | 13201 | 14064 | 11265 | 12210 | 11657 | 0.052396112 | 0.00839848 |
| BJK46_000920 | 6535 | 7552 | 4938 | 11907 | 10875 | 12945 | 1.061766344 | 2.56E-08 |
| BJK46_000925 | 2929 | 3071 | 3120 | 1740 | 1933 | 1770 | -0.72367274 | 1.36E-22 |
| BJK46_000930 | 4593 | 5138 | 6082 | 3279 | 3677 | 3119 | -0.59232689 | 8.17E-18 |
| BJK46_000935 | 3416 | 3735 | 4314 | 2295 | 2640 | 2082 | -0.67722828 | 2.47E-18 |
| BJK46_000940 | 4384 | 4505 | 5192 | 2784 | 3219 | 2804 | -0.63203142 | 4.72E-22 |
| BJK46_000945 | 1814 | 2100 | 2039 | 3138 | 2995 | 3412 | 0.705954837 | 7.95E-11 |
| BJK46_000950 | 1184 | 1380 | 1312 | 2154 | 2141 | 2651 | 0.833518087 | 1.25E-11 |
| BJK46_000955 | 415 | 556 | 421 | 60607 | 50834 | 65969 | 7.061778528 | 9.00E-300 |
| BJK46_000960 | 4638 | 4861 | 5712 | 4885 | 5421 | 4902 | 0.066434813 | 0.4052344 |
| BJK46_000965 | 4022 | 4145 | 4954 | 3905 | 4219 | 4016 | -0.05479822 | 0.02637913 |
| BJK46_000970 | 5674 | 6215 | 7234 | 4775 | 5441 | 4709 | -0.28291172 | 3.49E-08 |
| BJK46_000975 | 3228 | 3326 | 3918 | 2684 | 3262 | 2868 | -0.21337831 | 0.00011425 |
| BJK46_000980 | 3022 | 3261 | 3059 | 1915 | 2017 | 2279 | -0.5672493 | 2.78E-11 |
| BJK46_000985 | 39828 | 61084 | 31781 | 19901 | 13109 | 20142 | -0.99846868 | 2.20E-09 |
| BJK46_000990 | 17480 | 18283 | 20504 | 17227 | 18307 | 18018 | 0.187661059 | 0.16443363 |
| BJK46_000995 | 14249 | 14608 | 16205 | 14261 | 14917 | 14732 | 0.196772323 | 0.2908267 |
| BJK46_001000 | 4750 | 4957 | 5272 | 10008 | 9445 | 10086 | 1.078543427 | 7.21E-27 |
| BJK46_001005 | 7023 | 6995 | 7784 | 12028 | 12992 | 13929 | 0.993817187 | 4.50E-19 |
| BJK46_001010 | 21408 | 22106 | 24188 | 27490 | 27713 | 28528 | 0.59476641 | 0.01485739 |
| BJK46_001015 | 22168 | 22637 | 26248 | 21487 | 21606 | 19616 | 0.097326603 | 0.01859853 |
| BJK46_001020 | 31585 | 32954 | 39271 | 31681 | 31423 | 27733 | 0.123407066 | 0.0207883 |
| BJK46_001025 | 2593 | 2950 | 3192 | 3282 | 3613 | 3256 | 0.25386439 | 0.0434402 |
| BJK46_001030 | 3994 | 4252 | 4325 | 3641 | 4211 | 3658 | -0.08059511 | 0.01106841 |
| BJK46_001035 | 11654 | 12882 | 14238 | 4038 | 4624 | 4410 | -1.45661474 | 5.55E-96 |
| BJK46_001040 | 293 | 309 | 282 | 318 | 637 | 367 | -0.14177787 | 0.0441705 |
| BJK46_001045 | 216 | 218 | 216 | 187 | 391 | 235 | -0.59713095 | 0.35977408 |
| BJK46_001050 | 775 | 763 | 730 | 417 | 881 | 612 | -0.66516682 | 0.12905457 |
| BJK46_001055 | 9376 | 9758 | 9496 | 12632 | 12250 | 14282 | 0.633905563 | 8.71E-05 |
| BJK46_001060 | 20351 | 19928 | 23221 | 27709 | 26555 | 26097 | 0.623959563 | 0.00767737 |
| BJK46_001065 | 10897 | 11153 | 11108 | 15050 | 15397 | 16834 | 0.725188357 | 3.61E-06 |
| BJK46_001070 | 390 | 410 | 444 | 479 | 501 | 463 | -0.32960781 | 0.40005142 |
| BJK46_001075 | 5695 | 6372 | 6965 | 7429 | 7936 | 7129 | 0.339768716 | 0.01539575 |
| BJK46_001080 | 10172 | 10654 | 11382 | 3954 | 4447 | 3464 | -1.36030085 | 3.31E-66 |
| BJK46_001085 | 1779 | 1972 | 1912 | 2722 | 2825 | 2730 | 0.569257642 | 2.03E-08 |
| BJK46_001100 | 420 | 481 | 495 | 358 | 512 | 385 | -0.72074203 | 0.24545211 |
| BJK46_001105 | 7596 | 8110 | 8546 | 8528 | 8637 | 8721 | 0.224257197 | 0.6726226 |
| BJK46_001110 | 15280 | 16186 | 17479 | 14360 | 13913 | 13625 | 0.012148698 | 0.00347839 |
| BJK46_001115 | 11420 | 12123 | 12925 | 9011 | 9237 | 9157 | -0.23655106 | 3.19E-09 |
| BJK46_001120 | 6919 | 7870 | 8293 | 5936 | 5950 | 5906 | -0.2795267 | 7.57E-09 |
| BJK46_001125 | 8733 | 9102 | 10344 | 7393 | 7432 | 6771 | -0.25572255 | 5.22E-08 |
| BJK46_001130 | 1145 | 995 | 1253 | 845 | 1227 | 951 | -0.33246416 | 0.12504069 |
| BJK46_001135 | 2240 | 2428 | 2512 | 2443 | 2600 | 2560 | 0.107262355 | 0.80371758 |
| BJK46_001140 | 6220 | 7356 | 6678 | 7071 | 7480 | 8167 | 0.267753346 | 0.25233143 |
| BJK46_001145 | 4089 | 4949 | 4669 | 4873 | 5336 | 5793 | 0.285309686 | 0.05716785 |
| BJK46_001150 | 2062 | 2496 | 2285 | 2349 | 2457 | 2775 | 0.171105882 | 0.3791228 |
| BJK46_001155 | 306 | 370 | 414 | 434 | 917 | 551 | 0.216836187 | 0.00234754 |
| BJK46_001160 | 307 | 332 | 391 | 621 | 1106 | 783 | 0.782446006 | 4.79E-09 |
| BJK46_001165 | 690 | 673 | 715 | 1877 | 2826 | 2168 | 1.614865638 | 1.35E-34 |
| BJK46_001170 | 2146 | 2087 | 2030 | 4400 | 4418 | 4539 | 1.125002885 | 1.42E-28 |
| BJK46_001175 | 312 | 358 | 288 | 424 | 566 | 461 | -0.01278354 | 0.00938236 |
| BJK46_001180 | 1468 | 1877 | 1692 | 1554 | 1744 | 1803 | 0.015880811 | 0.68883434 |
| BJK46_001185 | 525 | 617 | 401 | 438 | 499 | 693 | -0.40061114 | 0.98112205 |
| BJK46_001190 | 1672 | 1998 | 1992 | 2562 | 2662 | 2505 | 0.472618445 | 8.67E-06 |
| BJK46_001195 | 2875 | 3201 | 3085 | 4059 | 4182 | 4109 | 0.473537987 | 3.84E-06 |
| BJK46_001200 | 994 | 1026 | 1105 | 713 | 891 | 920 | -0.52356313 | 0.01098894 |
| BJK46_001205 | 431 | 468 | 470 | 2510 | 2801 | 2706 | 2.41105772 | 1.63E-113 |
| BJK46_001210 | 1046 | 1104 | 1132 | 2094 | 2889 | 2319 | 1.126960927 | 4.13E-24 |
| BJK46_001215 | 1855 | 1966 | 2111 | 2501 | 3015 | 2512 | 0.453807006 | 1.54E-05 |
| BJK46_001220 | 241 | 267 | 266 | 439 | 561 | 528 | 0.331163004 | 2.19E-06 |
| BJK46_001225 | 419 | 383 | 522 | 1799 | 2192 | 1678 | 1.879820478 | 6.34E-42 |
| BJK46_001230 | 566 | 549 | 714 | 2321 | 2664 | 2075 | 1.843255565 | 2.14E-49 |
| BJK46_001235 | 918 | 946 | 1159 | 3526 | 4156 | 3254 | 1.864755898 | 1.50E-72 |
| BJK46_001240 | 566 | 624 | 620 | 2034 | 2146 | 1936 | 1.620443361 | 1.68E-44 |
| BJK46_001245 | 28 | 29 | 23 | 129 | 277 | 143 | 1.178227427 | 1.94E-15 |
| BJK46_001250 | 2035 | 1986 | 2410 | 2758 | 3359 | 2758 | 0.487912711 | 9.96E-06 |
| BJK46_001255 | 602 | 560 | 782 | 604 | 885 | 683 | -0.19594699 | 0.61128376 |
| BJK46_001260 | 998 | 1231 | 1278 | 1082 | 1330 | 1087 | -0.11793075 | 0.61994985 |
| BJK46_001265 | 571 | 630 | 756 | 1113 | 1396 | 1139 | 0.669649708 | 1.24E-08 |
| BJK46_001270 | 425 | 473 | 497 | 1063 | 1470 | 1172 | 1.094172415 | 1.87E-17 |
| BJK46_001275 | 381 | 407 | 390 | 1075 | 1652 | 1197 | 1.380346897 | 1.24E-21 |
| BJK46_001280 | 82 | 96 | 68 | 294 | 708 | 392 | 1.444515494 | 5.01E-15 |
| BJK46_001285 | 93 | 87 | 71 | 352 | 836 | 484 | 1.731760452 | 2.62E-18 |
| BJK46_001290 | 572 | 654 | 617 | 768 | 872 | 889 | 0.130130515 | 0.01490852 |
| BJK46_001295 | 1779 | 1941 | 1771 | 8751 | 8100 | 8755 | 2.26964705 | 2.45E-115 |
| BJK46_001300 | 972 | 1020 | 1229 | 1593 | 1896 | 1629 | 0.607620905 | 2.48E-07 |
| BJK46_001305 | 1203 | 1252 | 1470 | 2125 | 2391 | 2191 | 0.771535218 | 2.15E-14 |
| BJK46_001310 | 669 | 778 | 973 | 1736 | 2103 | 1844 | 1.147591745 | 5.67E-20 |
| BJK46_001315 | 567 | 552 | 701 | 1932 | 2442 | 1987 | 1.676782837 | 1.42E-41 |
| BJK46_001320 | 1374 | 1439 | 1420 | 1617 | 1752 | 1862 | 0.281541021 | 0.02600384 |
| BJK46_001325 | 192 | 192 | 188 | 910 | 1211 | 989 | 1.881365198 | 5.85E-36 |
| BJK46_001330 | 1623 | 1702 | 1949 | 1161 | 1515 | 1231 | -0.46812821 | 6.72E-06 |
| BJK46_001335 | 5817 | 6311 | 6509 | 5081 | 5558 | 5711 | -0.11577265 | 0.00072115 |
| BJK46_001340 | 1126 | 1231 | 1291 | 3744 | 4666 | 3851 | 1.763425946 | 4.54E-82 |
| BJK46_001345 | 441 | 558 | 457 | 492 | 526 | 611 | -0.31850677 | 0.61280498 |
| BJK46_001350 | 501 | 621 | 538 | 528 | 584 | 633 | -0.36152038 | 0.96587872 |
| BJK46_001355 | 5041 | 5560 | 6154 | 1361 | 1758 | 1422 | -1.85778749 | 2.05E-114 |
| BJK46_001360 | 2566 | 2934 | 2881 | 3196 | 3624 | 3442 | 0.325545297 | 0.00368857 |
| BJK46_001365 | 248 | 262 | 274 | 655 | 1159 | 753 | 1.141325453 | 2.92E-14 |
| BJK46_001370 | 276 | 330 | 313 | 702 | 1336 | 790 | 1.096210372 | 5.08E-12 |
| BJK46_001375 | 336 | 307 | 356 | 676 | 1091 | 716 | 0.805237617 | 1.40E-09 |
| BJK46_001380 | 626 | 616 | 677 | 1312 | 2001 | 1304 | 1.050589418 | 3.87E-13 |
| BJK46_001385 | 46 | 39 | 23 | 87 | 188 | 166 | 0.512891042 | 2.39E-08 |
| BJK46_001390 | 127 | 101 | 87 | 572 | 881 | 619 | 1.911792458 | 5.79E-26 |
| BJK46_001395 | 157 | 122 | 155 | 769 | 1338 | 897 | 2.129679111 | 4.59E-31 |
| BJK46_001400 | 122 | 119 | 97 | 564 | 1044 | 652 | 1.936202137 | 5.23E-25 |
| BJK46_001405 | 94 | 92 | 84 | 431 | 916 | 530 | 1.856517971 | 2.54E-22 |
| BJK46_001410 | 2741 | 2875 | 3053 | 1895 | 2379 | 2105 | -0.42357294 | 2.02E-09 |
| BJK46_001415 | 1188 | 1249 | 1241 | 756 | 971 | 844 | -0.69286333 | 2.36E-05 |
| BJK46_001420 | 2720 | 2768 | 3185 | 1454 | 1747 | 1670 | -0.81421854 | 5.76E-24 |
| BJK46_001425 | 4851 | 4704 | 6035 | 2839 | 3201 | 2893 | -0.74847721 | 2.77E-24 |
| BJK46_001430 | 200 | 196 | 185 | 274 | 654 | 354 | 0.27238625 | 0.00015789 |
| BJK46_001435 | 116 | 149 | 137 | 183 | 384 | 253 | -0.00227576 | 0.00033662 |
| BJK46_001440 | 81 | 93 | 110 | 165 | 346 | 223 | 0.221504636 | 2.85E-06 |
| BJK46_001445 | 149 | 136 | 154 | 156 | 324 | 198 | -0.43586661 | 0.04265145 |
| BJK46_001450 | 292 | 336 | 343 | 384 | 718 | 464 | 0.050062856 | 0.00617158 |
| BJK46_001455 | 301 | 410 | 345 | 286 | 346 | 352 | -0.77095958 | 0.42667822 |
| BJK46_001460 | 414 | 537 | 453 | 402 | 438 | 431 | -0.68312972 | 0.27569105 |
| BJK46_001465 | 53 | 50 | 63 | 73 | 79 | 44 | -1.37097258 | 0.56651578 |
| BJK46_001470 | 120 | 129 | 110 | 50 | 128 | 90 | -1.81254473 | 0.09592352 |
| BJK46_001475 | 774 | 777 | 838 | 1157 | 3028 | 1672 | 1.074278143 | 1.81E-07 |
| BJK46_001480 | 800 | 986 | 1072 | 1164 | 1375 | 1185 | 0.242784893 | 0.01727955 |
| BJK46_001485 | 1280 | 1434 | 1592 | 1598 | 2015 | 1620 | 0.257975799 | 0.04100959 |
| BJK46_001490 | 1367 | 1503 | 1684 | 1728 | 2131 | 1649 | 0.26132939 | 0.04451568 |
| BJK46_001495 | 1246 | 1358 | 1544 | 1578 | 1843 | 1506 | 0.217480924 | 0.08963454 |
| BJK46_001500 | 2384 | 2645 | 2932 | 2932 | 3531 | 2698 | 0.227174694 | 0.12223978 |
| BJK46_001505 | 1201 | 1420 | 1299 | 1443 | 1653 | 1406 | 0.147291564 | 0.25695472 |
| BJK46_001510 | 1711 | 1881 | 1981 | 2110 | 2358 | 1871 | 0.194454962 | 0.19950687 |
| BJK46_001515 | 2122 | 2371 | 2431 | 2471 | 2814 | 2353 | 0.162895692 | 0.35620615 |
| BJK46_001520 | 1355 | 1552 | 1648 | 1849 | 2014 | 1837 | 0.321122912 | 0.00566604 |
| BJK46_001525 | 4592 | 4906 | 5434 | 7080 | 7577 | 6980 | 0.614172144 | 4.69E-11 |
| BJK46_001530 | 26146 | 27259 | 23692 | 38156 | 43829 | 53466 | 1.118843015 | 8.03E-08 |
| BJK46_001535 | 757 | 757 | 790 | 1559 | 2180 | 1725 | 1.11970282 | 1.66E-18 |
| BJK46_001540 | 1007 | 1301 | 1861 | 589 | 1036 | 674 | -1.03371835 | 7.25E-06 |
| BJK46_001545 | 1061 | 1092 | 1444 | 1206 | 2012 | 1281 | 0.226968911 | 0.12086034 |
| BJK46_001550 | 654 | 621 | 695 | 801 | 1196 | 814 | 0.198720386 | 0.01183626 |
| BJK46_001555 | 846 | 999 | 965 | 1841 | 1956 | 1867 | 0.945998061 | 7.56E-17 |
| BJK46_001560 | 2679 | 3180 | 3023 | 5933 | 5741 | 6035 | 1.050298946 | 3.12E-28 |
| BJK46_001565 | 2980 | 3138 | 3578 | 1595 | 2016 | 1601 | -0.8778721 | 1.76E-26 |
| BJK46_001570 | 928 | 1096 | 1137 | 1331 | 1622 | 1626 | 0.448337913 | 0.00013651 |
| BJK46_001575 | 4892 | 5453 | 5374 | 7111 | 7843 | 8289 | 0.646798088 | 3.10E-10 |
| BJK46_001580 | 16171 | 16295 | 16159 | 12982 | 13089 | 13098 | -0.08232956 | 0.00015897 |
| BJK46_001585 | 80 | 86 | 82 | 180 | 463 | 287 | 0.729443645 | 1.90E-09 |
| BJK46_001590 | 195 | 212 | 221 | 435 | 1035 | 568 | 0.961293915 | 1.51E-09 |
| BJK46_001595 | 197 | 191 | 208 | 388 | 903 | 533 | 0.847616248 | 6.80E-09 |
| BJK46_001600 | 189 | 191 | 205 | 319 | 688 | 422 | 0.476909486 | 2.24E-06 |
| BJK46_001605 | 411 | 438 | 424 | 402 | 759 | 517 | -0.16346533 | 0.14651451 |
| BJK46_001610 | 396 | 399 | 406 | 467 | 794 | 585 | 0.084175877 | 0.00814854 |
| BJK46_001615 | 177 | 162 | 185 | 209 | 532 | 326 | 0.08153074 | 0.00089321 |
| BJK46_001620 | 93 | 104 | 84 | 79 | 223 | 142 | -0.69200353 | 0.07466936 |
| BJK46_001625 | 231 | 234 | 237 | 275 | 585 | 359 | -0.00702556 | 0.00478327 |
| BJK46_001630 | 227 | 302 | 243 | 283 | 364 | 381 | -0.32877831 | 0.11203452 |
| BJK46_001635 | 110 | 137 | 119 | 174 | 295 | 186 | -0.24460949 | 0.00285213 |
| BJK46_001640 | 5103 | 5725 | 5573 | 3541 | 3651 | 3672 | -0.53745563 | 1.88E-17 |
| BJK46_001645 | 1448 | 1577 | 1641 | 1344 | 1453 | 1302 | -0.22540389 | 0.02030449 |
| BJK46_001650 | 1338 | 1399 | 1424 | 1180 | 1188 | 1166 | -0.31619306 | 0.01239221 |
| BJK46_001655 | 2829 | 3002 | 3497 | 3656 | 4201 | 3337 | 0.300957707 | 0.01999762 |
| BJK46_001660 | 14087 | 13402 | 12581 | 6044 | 7319 | 8471 | -0.72825522 | 3.12E-15 |
| BJK46_001665 | 861 | 939 | 880 | 2245 | 2539 | 2721 | 1.449365015 | 5.94E-38 |
| BJK46_001670 | 3729 | 3329 | 4266 | 4267 | 4690 | 3824 | 0.225964056 | 0.24264695 |
| BJK46_001675 | 43038 | 42556 | 54236 | 56518 | 57930 | 48038 | 0.581580532 | 0.17620508 |
| BJK46_001680 | 11673 | 12105 | 14522 | 14646 | 16016 | 14359 | 0.4606116 | 0.05513989 |
| BJK46_001685 | 27479 | 28225 | 26685 | 35628 | 34936 | 37456 | 0.699885165 | 0.00353468 |
| BJK46_001690 | 1261 | 1390 | 1408 | 1715 | 2051 | 1903 | 0.461969482 | 3.04E-05 |
| BJK46_001695 | 2538 | 2862 | 3111 | 2891 | 3174 | 2921 | 0.112106848 | 0.8188527 |
| BJK46_001700 | 570 | 587 | 583 | 1185 | 1811 | 1365 | 1.08410709 | 6.33E-15 |
| BJK46_001705 | 188 | 182 | 180 | 338 | 817 | 444 | 0.707686055 | 1.93E-07 |
| BJK46_001710 | 199 | 181 | 184 | 357 | 972 | 492 | 0.86391494 | 8.38E-08 |
| BJK46_001715 | 2764 | 2831 | 2863 | 953 | 1233 | 1060 | -1.39122294 | 1.71E-49 |
| BJK46_001720 | 2207 | 2106 | 2274 | 779 | 1031 | 921 | -1.32781549 | 1.63E-31 |
| BJK46_001725 | 1914 | 2202 | 2402 | 706 | 945 | 797 | -1.48537519 | 7.96E-37 |
| BJK46_001730 | 5102 | 5755 | 6361 | 1933 | 2411 | 1955 | -1.41249759 | 4.18E-75 |
| BJK46_007945 | 11859 | 13051 | 12367 | 16592 | 16162 | 17703 | 0.665493342 | 0.00028216 |
| BJK46_007950 | 4753 | 4940 | 5901 | 9716 | 10443 | 9769 | 1.048546056 | 1.96E-29 |
| BJK46_007955 | 2703 | 2783 | 3225 | 6275 | 6647 | 6367 | 1.204082406 | 2.61E-47 |
| BJK46_007960 | 3332 | 3410 | 4098 | 2446 | 2542 | 2151 | -0.56952793 | 1.21E-13 |
| BJK46_007965 | 4993 | 5067 | 5463 | 5763 | 5681 | 6299 | 0.261678033 | 0.11025231 |
| BJK46_007970 | 2487 | 2879 | 2795 | 3485 | 3438 | 3623 | 0.405219867 | 0.00027034 |
| BJK46_007975 | 9586 | 10432 | 11218 | 6882 | 7146 | 6688 | -0.45877119 | 2.83E-17 |
| BJK46_007980 | 2871 | 3286 | 3630 | 3210 | 3394 | 3120 | 0.032153395 | 0.39067801 |
| BJK46_007985 | 486 | 581 | 585 | 581 | 842 | 603 | -0.12606371 | 0.21782972 |
| BJK46_007990 | 3172 | 3300 | 3584 | 6321 | 6089 | 6302 | 0.954207303 | 2.08E-26 |
| BJK46_007995 | 4933 | 5287 | 5419 | 10466 | 10286 | 11647 | 1.159181639 | 4.17E-29 |
| BJK46_008000 | 6031 | 6596 | 7320 | 11822 | 12408 | 11474 | 0.984004098 | 2.31E-23 |
| BJK46_008005 | 1296 | 1387 | 1665 | 3046 | 3618 | 2986 | 1.170994275 | 2.13E-32 |
| BJK46_008010 | 1531 | 1723 | 1863 | 5615 | 6229 | 5722 | 1.81623718 | 3.67E-113 |
| BJK46_008015 | 1125 | 1241 | 1130 | 25061 | 25323 | 28876 | 4.570112615 | 6.13E-307 |
| BJK46_008020 | 7517 | 7885 | 9248 | 9654 | 10231 | 9063 | 0.376248191 | 0.03357545 |
| BJK46_008025 | 7519 | 8585 | 8344 | 5457 | 5978 | 6490 | -0.35181383 | 2.37E-09 |
| BJK46_008030 | 2466 | 2934 | 2907 | 1663 | 1852 | 1697 | -0.65032833 | 1.48E-17 |
| BJK46_008035 | 5768 | 6300 | 6835 | 8522 | 8368 | 8427 | 0.52957006 | 4.08E-06 |
| BJK46_008040 | 12315 | 13739 | 15817 | 18046 | 18797 | 17059 | 0.611357586 | 0.00164382 |
| BJK46_008045 | 3242 | 4107 | 4106 | 5294 | 5387 | 5454 | 0.55883938 | 1.04E-07 |
| BJK46_008050 | 17295 | 18384 | 21184 | 18503 | 21097 | 21601 | 0.37106315 | 0.64866894 |
| BJK46_008055 | 17602 | 17972 | 20533 | 18618 | 21247 | 22044 | 0.404616655 | 0.42952162 |
| BJK46_008060 | 8163 | 8576 | 9304 | 3679 | 4053 | 3823 | -1.09860795 | 1.67E-66 |
| BJK46_008065 | 4191 | 4352 | 4752 | 1631 | 1813 | 1670 | -1.35014642 | 1.72E-75 |
| BJK46_008070 | 2658 | 2989 | 3363 | 53964 | 55158 | 50957 | 4.364541582 | 7.55E-274 |
| BJK46_008075 | 3621 | 4091 | 4825 | 71248 | 74982 | 69276 | 4.362913814 | 5.04E-252 |
| BJK46_008080 | 2761 | 3259 | 3537 | 54828 | 57735 | 55772 | 4.362332691 | 2.29E-274 |
| BJK46_008085 | 1273 | 1486 | 1583 | 37406 | 37005 | 42903 | 4.86766802 | 1.42E-302 |
| BJK46_008090 | 9826 | 10176 | 12034 | 9620 | 9973 | 8894 | -0.00080352 | 0.00648216 |
| BJK46_008095 | 2347 | 2530 | 2602 | 1498 | 1782 | 1723 | -0.56792084 | 3.39E-13 |
| BJK46_008100 | 4686 | 5303 | 5940 | 6843 | 7288 | 6820 | 0.479753649 | 5.15E-06 |
| BJK46_008105 | 5337 | 6063 | 7144 | 7968 | 8529 | 7485 | 0.477285386 | 0.00015214 |
| BJK46_008110 | 2605 | 2829 | 2251 | 2670 | 2962 | 3497 | 0.273579197 | 0.15292365 |
| BJK46_008115 | 641 | 657 | 712 | 889 | 1264 | 1102 | 0.431476359 | 5.53E-05 |
| BJK46_008120 | 492 | 507 | 581 | 2577 | 2943 | 2514 | 2.236528792 | 3.50E-100 |
| BJK46_008125 | 2373 | 2806 | 2507 | 12827 | 12397 | 13538 | 2.408979162 | 1.98E-124 |
| BJK46_008130 | 312 | 315 | 341 | 369 | 546 | 399 | -0.20752445 | 0.06904918 |
| BJK46_008135 | 184 | 187 | 229 | 214 | 310 | 274 | -0.46451359 | 0.1182353 |
| BJK46_008140 | 3743 | 3956 | 4511 | 3149 | 3447 | 3283 | -0.25880404 | 1.09E-06 |
| BJK46_008145 | 7151 | 7248 | 7784 | 7580 | 8116 | 7925 | 0.203632253 | 0.72267239 |
| BJK46_008150 | 21912 | 24080 | 29195 | 14805 | 14935 | 13527 | -0.52855469 | 5.76E-16 |
| BJK46_008155 | 18005 | 19799 | 24472 | 11193 | 11400 | 9675 | -0.70774687 | 2.73E-20 |
| BJK46_008160 | 4248 | 4666 | 5724 | 2688 | 3288 | 3041 | -0.646536 | 1.24E-18 |
| BJK46_008165 | 25143 | 26798 | 27740 | 14366 | 11959 | 13078 | -0.76061423 | 2.62E-20 |
| BJK46_008170 | 4558 | 5059 | 5673 | 9282 | 8811 | 9395 | 0.945033287 | 9.63E-21 |
| BJK46_008175 | 5068 | 5562 | 6493 | 3483 | 3974 | 3521 | -0.57972901 | 1.57E-20 |
| BJK46_008180 | 4065 | 4555 | 5519 | 2396 | 3003 | 2370 | -0.82090745 | 5.81E-24 |
| BJK46_008185 | 1840 | 2105 | 2054 | 1265 | 1374 | 1298 | -0.62059976 | 7.84E-12 |
| BJK46_008190 | 14053 | 16286 | 13361 | 11164 | 9750 | 12669 | -0.17406936 | 0.00060413 |
| BJK46_008195 | 20670 | 22274 | 22216 | 5742 | 6339 | 5924 | -1.66656103 | 4.78E-96 |
| BJK46_008200 | 2915 | 3055 | 3242 | 2785 | 2668 | 2695 | -0.14624248 | 0.00506393 |
| BJK46_008205 | 6318 | 6952 | 7558 | 6144 | 6081 | 6120 | -0.09284818 | 0.00137016 |
| BJK46_008210 | 7182 | 7675 | 9024 | 6390 | 6730 | 5808 | -0.23168338 | 1.67E-06 |
| BJK46_008215 | 7388 | 8260 | 9257 | 7135 | 7779 | 7257 | -0.04576613 | 0.00268944 |
| BJK46_008220 | 1788 | 1891 | 2253 | 3955 | 4157 | 3723 | 1.02988613 | 2.37E-28 |
| BJK46_008225 | 2110 | 2256 | 2411 | 4112 | 4383 | 4162 | 0.935494394 | 3.95E-28 |
| BJK46_008230 | 867 | 941 | 1018 | 552 | 768 | 650 | -0.81693629 | 0.00018402 |
| BJK46_008235 | 6686 | 7296 | 8511 | 3042 | 3605 | 3224 | -1.1231084 | 2.04E-60 |
| BJK46_008240 | 24389 | 26150 | 30165 | 16832 | 18221 | 17395 | -0.34444133 | 3.67E-12 |
| BJK46_008245 | 10008 | 10455 | 11720 | 6948 | 7219 | 6928 | -0.47054364 | 7.09E-18 |
| BJK46_008250 | 7045 | 7494 | 8330 | 8393 | 9163 | 8728 | 0.327894327 | 0.06368143 |
| BJK46_008255 | 5617 | 6029 | 6426 | 11966 | 12382 | 11448 | 1.118647852 | 3.47E-32 |
| BJK46_008260 | 4144 | 4230 | 4666 | 5025 | 5606 | 5631 | 0.378653463 | 0.00056072 |
| BJK46_008265 | 1556 | 1627 | 1902 | 1181 | 1495 | 1269 | -0.40083242 | 0.00011008 |
| BJK46_008270 | 2920 | 3238 | 3234 | 3717 | 3710 | 4092 | 0.334540053 | 0.00615003 |
| BJK46_008275 | 1397 | 1684 | 1679 | 2013 | 2286 | 2183 | 0.454813453 | 3.12E-05 |
| BJK46_008280 | 1166 | 1133 | 1241 | 966 | 926 | 954 | -0.46887939 | 0.00683194 |
| BJK46_008285 | 3208 | 3292 | 3215 | 3154 | 3115 | 3133 | -0.01256893 | 0.19920205 |
| BJK46_008290 | 2419 | 2592 | 2728 | 2168 | 2527 | 2248 | -0.13468303 | 0.00592701 |
| BJK46_008295 | 677 | 763 | 776 | 709 | 922 | 763 | -0.19188484 | 0.77866735 |
| BJK46_008300 | 5779 | 5998 | 6432 | 4451 | 4083 | 4286 | -0.44271879 | 6.74E-12 |
| BJK46_008305 | 5412 | 6041 | 6548 | 3993 | 3748 | 3818 | -0.57555159 | 2.99E-18 |
| BJK46_008310 | 11717 | 12499 | 13637 | 11304 | 12212 | 11448 | 0.088082429 | 0.0305959 |
| BJK46_008315 | 896 | 867 | 1064 | 1006 | 1305 | 1121 | 0.112484279 | 0.13056951 |
| BJK46_008320 | 1682 | 1703 | 2033 | 1589 | 1875 | 1653 | -0.08197862 | 0.12635217 |
| BJK46_008325 | 2877 | 2809 | 3576 | 3173 | 3762 | 3167 | 0.164206224 | 0.47549125 |
| BJK46_008330 | 3994 | 4126 | 3311 | 1907 | 2235 | 2829 | -0.69516205 | 3.21E-08 |
| BJK46_008335 | 2532 | 3071 | 3471 | 2086 | 2114 | 1914 | -0.535204 | 2.05E-11 |
| BJK46_008340 | 2415 | 2862 | 3287 | 1911 | 2190 | 1962 | -0.46676111 | 2.49E-10 |
| BJK46_008345 | 2922 | 3271 | 3496 | 1584 | 1742 | 1467 | -0.994394 | 5.99E-37 |
| BJK46_008350 | 10047 | 11403 | 12748 | 7227 | 7562 | 6860 | -0.50997191 | 8.08E-19 |
| BJK46_008355 | 1737 | 1665 | 2086 | 1354 | 1397 | 1352 | -0.43277734 | 7.68E-06 |
| BJK46_008360 | 1428 | 1538 | 1667 | 958 | 1271 | 1061 | -0.57682672 | 3.77E-06 |
| BJK46_008365 | 7525 | 8182 | 8562 | 7862 | 7534 | 7284 | 0.020533875 | 0.05378332 |
| BJK46_008370 | 4610 | 5118 | 5313 | 4729 | 4509 | 4830 | -0.0343937 | 0.05165621 |
| BJK46_008375 | 9025 | 9266 | 8558 | 5792 | 6193 | 7967 | -0.3276971 | 2.83E-05 |
| BJK46_008380 | 14240 | 15451 | 18417 | 13800 | 14674 | 12166 | -0.00846367 | 0.00313861 |
| BJK46_008385 | 10754 | 11941 | 14014 | 9835 | 10394 | 8679 | -0.16104349 | 8.67E-06 |
| BJK46_008390 | 13063 | 14176 | 15186 | 12713 | 13254 | 12605 | 0.083379297 | 0.02061857 |
| BJK46_008395 | 9832 | 11192 | 11495 | 10137 | 10180 | 10280 | 0.088712739 | 0.06990801 |
| BJK46_008400 | 1548 | 1643 | 1758 | 2245 | 2437 | 2147 | 0.475391028 | 6.38E-06 |
| BJK46_008405 | 48371 | 52072 | 31146 | 48985 | 51694 | 75633 | 0.789067873 | 0.09957464 |
| BJK46_008410 | 8297 | 8936 | 9537 | 10187 | 10429 | 10228 | 0.36151877 | 0.06554872 |
| BJK46_008415 | 5134 | 5661 | 5136 | 6143 | 5822 | 6203 | 0.26173531 | 0.15843073 |
| BJK46_008420 | 7141 | 8401 | 9223 | 5500 | 5685 | 5502 | -0.46959318 | 1.32E-15 |
| BJK46_008425 | 6172 | 6869 | 7929 | 4191 | 4443 | 3940 | -0.6645659 | 8.10E-25 |
| BJK46_008430 | 28189 | 31201 | 36483 | 17973 | 19415 | 16813 | -0.53512618 | 1.63E-17 |
| BJK46_008435 | 20053 | 21611 | 24195 | 13071 | 13627 | 13328 | -0.46506526 | 3.11E-16 |
| BJK46_008440 | 34939 | 37314 | 33930 | 19168 | 17744 | 18392 | -0.65247474 | 2.67E-18 |
| BJK46_008445 | 9922 | 9975 | 11402 | 7682 | 8910 | 7718 | -0.21829617 | 7.59E-08 |
| BJK46_008450 | 8641 | 10657 | 10913 | 6674 | 6678 | 6364 | -0.48475758 | 1.16E-15 |
| BJK46_008455 | 11749 | 14009 | 14485 | 9452 | 9445 | 9426 | -0.31446064 | 4.14E-11 |
| BJK46_008460 | 762 | 883 | 857 | 419 | 687 | 500 | -1.03070016 | 0.00011426 |
| BJK46_008465 | 573 | 603 | 603 | 291 | 436 | 294 | -1.3721331 | 1.12E-05 |
| BJK46_008470 | 7157 | 7321 | 8275 | 1682 | 1867 | 1670 | -2.08000114 | 2.98E-183 |
| BJK46_008475 | 28755 | 31374 | 35377 | 28497 | 29808 | 25497 | 0.117095651 | 0.01721454 |
| BJK46_008480 | 1173 | 1337 | 1253 | 1629 | 1741 | 1649 | 0.375966219 | 0.00165196 |
| BJK46_008485 | 22540 | 26440 | 29712 | 4352 | 4729 | 4388 | -2.35918701 | 2.77E-142 |
| BJK46_008490 | 12947 | 13263 | 13460 | 143245 | 135604 | 137839 | 3.73576935 | 5.41E-168 |
| BJK46_008495 | 3885 | 4295 | 3974 | 7248 | 7082 | 8112 | 0.952290715 | 4.91E-19 |
| BJK46_008500 | 1577 | 1771 | 1818 | 932 | 1258 | 1109 | -0.71311264 | 1.65E-09 |
| BJK46_008505 | 1163 | 1220 | 1390 | 834 | 1302 | 899 | -0.4707431 | 0.01234703 |
| BJK46_008510 | 673 | 642 | 835 | 598 | 914 | 608 | -0.37500416 | 0.62976648 |
| BJK46_008515 | 270 | 320 | 301 | 628 | 790 | 692 | 0.704263945 | 1.16E-10 |
| BJK46_008520 | 1216 | 1355 | 1237 | 1629 | 1924 | 1951 | 0.500330908 | 4.32E-05 |
| BJK46_008525 | 4444 | 4648 | 5278 | 7220 | 7110 | 6369 | 0.600273964 | 6.88E-09 |
| BJK46_008530 | 22 | 21 | 11 | 35 | 98 | 54 | -0.19746177 | 1.81E-05 |
| BJK46_008535 | 131 | 151 | 157 | 821 | 678 | 618 | 1.55940479 | 7.73E-25 |
| BJK46_008540 | 19305 | 21205 | 18305 | 59449 | 66140 | 74629 | 2.087815887 | 1.71E-40 |
| BJK46_008545 | 79 | 80 | 94 | 187 | 331 | 225 | 0.402751918 | 1.40E-08 |
| BJK46_008550 | 3351 | 4347 | 2619 | 4848 | 5235 | 6511 | 0.758057107 | 0.00017833 |
| BJK46_008555 | 2810 | 3587 | 2117 | 3488 | 3588 | 4816 | 0.53606649 | 0.01950922 |
| BJK46_008560 | 15 | 8 | 16 | 80 | 206 | 91 | 1.351500552 | 4.88E-15 |
| BJK46_008565 | 2371 | 2846 | 3032 | 4217 | 3941 | 4006 | 0.604735361 | 1.23E-08 |
| BJK46_008570 | 2327 | 2709 | 2930 | 4226 | 3881 | 4084 | 0.655812829 | 4.57E-10 |
| BJK46_008575 | 291 | 294 | 309 | 112 | 270 | 155 | -1.70749359 | 0.00267934 |
| BJK46_008580 | 98 | 95 | 123 | 189 | 434 | 217 | 0.284254879 | 6.97E-06 |
| BJK46_008585 | 125 | 120 | 165 | 299 | 608 | 294 | 0.602007325 | 1.33E-07 |
| BJK46_008590 | 44 | 35 | 62 | 76 | 191 | 101 | -0.16099262 | 0.00010047 |
| BJK46_008595 | 64 | 56 | 75 | 112 | 282 | 160 | 0.138308562 | 6.17E-06 |
| BJK46_008600 | 121 | 108 | 129 | 168 | 365 | 207 | -0.05905995 | 0.00059536 |
| BJK46_008605 | 145 | 164 | 166 | 219 | 433 | 309 | 0.078298933 | 0.0001709 |
| BJK46_008610 | 933 | 1103 | 1250 | 1422 | 1580 | 1330 | 0.316391884 | 0.00683082 |
| BJK46_008615 | 1054 | 1154 | 1336 | 1256 | 1474 | 1242 | 0.07636752 | 0.40291703 |
| BJK46_008620 | 2819 | 2997 | 3395 | 3475 | 3867 | 3875 | 0.325116291 | 0.00465127 |
| BJK46_008625 | 2517 | 2743 | 3061 | 2607 | 2915 | 2496 | -0.02490649 | 0.16065006 |
| BJK46_008630 | 2139 | 2057 | 1691 | 2128 | 2062 | 2568 | 0.216181293 | 0.35187593 |
| BJK46_008635 | 1704 | 1812 | 2050 | 1188 | 1426 | 1256 | -0.54914326 | 7.64E-09 |
| BJK46_008640 | 2048 | 2243 | 2635 | 1635 | 1831 | 1524 | -0.45747853 | 1.61E-08 |
| BJK46_008645 | 11623 | 13724 | 15557 | 5390 | 5659 | 5170 | -1.19217331 | 3.25E-59 |
| BJK46_008650 | 9777 | 10762 | 12283 | 6608 | 7213 | 6766 | -0.5324136 | 5.62E-21 |
| BJK46_008655 | 16632 | 19072 | 18776 | 23288 | 22176 | 25930 | 0.658021047 | 0.00255516 |
| BJK46_008660 | 4106 | 4685 | 5161 | 7172 | 7678 | 6800 | 0.711065543 | 1.58E-14 |
| BJK46_008665 | 463 | 544 | 575 | 415 | 479 | 410 | -0.78997185 | 0.0573874 |
| BJK46_008670 | 2632 | 2754 | 2599 | 1720 | 2045 | 2186 | -0.40995721 | 1.19E-06 |
| BJK46_008675 | 3288 | 3570 | 3142 | 2416 | 2542 | 3010 | -0.30111399 | 0.00026506 |
| BJK46_008680 | 22752 | 23425 | 24719 | 36071 | 35499 | 36903 | 0.915965055 | 1.10E-07 |
| BJK46_008685 | 3026 | 3043 | 3485 | 2239 | 2365 | 2275 | -0.44422693 | 6.35E-11 |
| BJK46_008690 | 173 | 153 | 152 | 223 | 383 | 275 | -0.05405733 | 0.00103639 |
| BJK46_008695 | 127 | 144 | 151 | 168 | 324 | 204 | -0.32898703 | 0.01283227 |
| BJK46_008700 | 1902 | 2342 | 1873 | 4896 | 4161 | 4958 | 1.231701321 | 3.38E-21 |
| BJK46_008705 | 8823 | 10071 | 8912 | 27300 | 24609 | 28954 | 1.77985968 | 2.42E-35 |
| BJK46_008710 | 4985 | 4991 | 5458 | 6709 | 6648 | 6028 | 0.400921663 | 0.00084574 |
| BJK46_008715 | 967 | 1129 | 1152 | 1095 | 1418 | 1192 | 0.066135231 | 0.34325813 |
| BJK46_008720 | 338 | 347 | 427 | 355 | 509 | 350 | -0.50300282 | 0.7761427 |
| BJK46_008725 | 559 | 508 | 688 | 497 | 783 | 576 | -0.34685353 | 0.95989651 |
| BJK46_008730 | 270 | 275 | 338 | 312 | 419 | 293 | -0.50612225 | 0.48852048 |
| BJK46_008735 | 16714 | 19122 | 21107 | 8404 | 8819 | 8369 | -0.93896319 | 7.78E-42 |
| BJK46_008740 | 725 | 744 | 641 | 578 | 691 | 740 | -0.42319828 | 0.42666558 |
| BJK46_008745 | 3407 | 3920 | 3778 | 3219 | 3513 | 3505 | -0.07461491 | 0.02300387 |
| BJK46_008750 | 10719 | 13440 | 14858 | 3938 | 4322 | 3898 | -1.5671842 | 8.35E-85 |
| BJK46_008755 | 2850 | 3236 | 2906 | 4705 | 5029 | 5267 | 0.78589441 | 1.75E-14 |
| BJK46_008760 | 6778 | 7244 | 7553 | 42457 | 38565 | 42699 | 2.769435121 | 3.67E-103 |
| BJK46_008765 | 6878 | 7595 | 8031 | 49049 | 45751 | 50382 | 2.951398622 | 1.16E-118 |
| BJK46_008770 | 2316 | 2921 | 1990 | 3162 | 2346 | 3103 | 0.283427709 | 0.23000622 |
| BJK46_008775 | 56279 | 58851 | 67958 | 68390 | 70650 | 65128 | 0.55007646 | 0.34766135 |
| BJK46_008780 | 15024 | 15993 | 18018 | 19917 | 21145 | 19013 | 0.550747696 | 0.01500554 |
| BJK46_008785 | 2354 | 2473 | 2988 | 2615 | 3130 | 2646 | 0.132089065 | 0.63805957 |
| BJK46_008790 | 3808 | 4027 | 4702 | 4456 | 4862 | 4417 | 0.190171688 | 0.34611523 |
| BJK46_008795 | 8149 | 9305 | 9069 | 32856 | 31785 | 36540 | 2.181545154 | 1.29E-62 |
| BJK46_008800 | 1579 | 1918 | 2145 | 2406 | 2328 | 2185 | 0.320496662 | 0.01475366 |
| BJK46_008805 | 4330 | 4689 | 5269 | 5097 | 5178 | 4938 | 0.155761452 | 0.66735516 |
| BJK46_008810 | 3004 | 3291 | 3668 | 2934 | 3129 | 2960 | -0.10476046 | 0.00851484 |
| BJK46_008815 | 28647 | 27579 | 30185 | 1123 | 1426 | 1112 | -4.49401966 | 2.34187116128751e-321 |
| BJK46_008820 | 30296 | 29902 | 31509 | 970 | 1304 | 1052 | -4.72256598 | 0 |
| BJK46_008825 | 13117 | 13387 | 14015 | 424 | 580 | 455 | -4.78198024 | 0 |
| BJK46_008830 | 21920 | 20533 | 23068 | 712 | 1008 | 819 | -4.64909524 | 0 |
| BJK46_008835 | 25677 | 25100 | 26915 | 880 | 1223 | 949 | -4.62010499 | 0 |
| BJK46_008840 | 30521 | 31092 | 33211 | 1187 | 1726 | 1166 | -4.4756786 | 4.33E-245 |
| BJK46_008845 | 8007 | 8280 | 8991 | 331 | 508 | 345 | -4.43754643 | 0 |
| BJK46_008850 | 5192 | 5639 | 5643 | 195 | 323 | 224 | -4.62147694 | 3.47E-294 |
| BJK46_008855 | 8267 | 9690 | 9160 | 385 | 552 | 442 | -4.29465131 | 0 |
| BJK46_008860 | 8159 | 9538 | 9361 | 477 | 795 | 597 | -3.86107009 | 1.28E-235 |
| BJK46_008865 | 2514 | 3107 | 2803 | 239 | 367 | 280 | -3.48657591 | 1.40E-126 |
| BJK46_008870 | 20259 | 21627 | 21527 | 14787 | 14185 | 13081 | -0.34381995 | 2.21E-10 |
| BJK46_008875 | 3618 | 3888 | 4344 | 3994 | 4746 | 4370 | 0.197377688 | 0.26963926 |
| BJK46_008880 | 558 | 644 | 641 | 22990 | 22460 | 24014 | 5.282832999 | 0 |
| BJK46_008885 | 101815 | 108012 | 112311 | 67422 | 52422 | 56966 | -0.46082107 | 2.14E-12 |
| BJK46_008890 | 153828 | 161144 | 167971 | 105968 | 85261 | 95057 | -0.30478413 | 1.41E-10 |
| BJK46_008895 | 69138 | 70392 | 75931 | 47886 | 40876 | 45034 | -0.31477669 | 3.39E-10 |
| BJK46_008900 | 30717 | 30990 | 33081 | 22173 | 18125 | 19289 | -0.38793346 | 1.90E-09 |
| BJK46_008905 | 549 | 518 | 604 | 706 | 976 | 675 | 0.111952743 | 0.01895633 |
| BJK46_008910 | 3366 | 3961 | 3626 | 2884 | 2938 | 3406 | -0.21039658 | 0.00145013 |
| BJK46_008915 | 345 | 435 | 437 | 506 | 774 | 566 | 0.087324029 | 0.00646676 |
| BJK46_008920 | 156 | 156 | 170 | 163 | 301 | 184 | -0.6052158 | 0.1737202 |
| BJK46_008925 | 189 | 222 | 265 | 227 | 372 | 278 | -0.45685443 | 0.17435554 |
| BJK46_008930 | 187 | 207 | 211 | 247 | 384 | 298 | -0.22990103 | 0.012838 |
| BJK46_008935 | 108 | 115 | 145 | 152 | 282 | 186 | -0.34824008 | 0.00903139 |
| BJK46_008940 | 240 | 269 | 268 | 332 | 590 | 427 | 0.080175686 | 0.0012303 |
| BJK46_008945 | 223 | 203 | 224 | 326 | 662 | 405 | 0.318665898 | 4.68E-05 |
| BJK46_008950 | 54 | 48 | 59 | 58 | 161 | 114 | -0.52473263 | 0.00326939 |
| BJK46_008955 | 87 | 100 | 67 | 115 | 317 | 153 | -0.10804538 | 0.00115697 |
| BJK46_008960 | 8092 | 8809 | 9745 | 9056 | 10090 | 9469 | 0.252552325 | 0.5842599 |
| BJK46_008965 | 9634 | 10896 | 10313 | 13613 | 14414 | 15123 | 0.686098142 | 2.92E-06 |
| BJK46_008970 | 8928 | 9385 | 9282 | 10429 | 10649 | 11701 | 0.41080637 | 0.03787802 |
| BJK46_008975 | 978 | 973 | 1070 | 1478 | 1566 | 1551 | 0.51388151 | 8.19E-06 |
| BJK46_008980 | 34158 | 36937 | 45050 | 38657 | 40844 | 36814 | 0.342286671 | 0.60862576 |
| BJK46_008985 | 10909 | 12306 | 11143 | 20140 | 17762 | 22019 | 1.033429614 | 7.09E-10 |
| BJK46_008990 | 2009 | 2201 | 2037 | 5514 | 5120 | 5159 | 1.375217515 | 9.24E-43 |
| BJK46_008995 | 3344 | 3451 | 3596 | 2748 | 2998 | 2961 | -0.22121416 | 5.06E-05 |
| BJK46_009000 | 18860 | 19880 | 22040 | 14882 | 14886 | 13916 | -0.22448571 | 3.28E-08 |
| BJK46_009005 | 5125 | 5620 | 6718 | 3498 | 3887 | 3160 | -0.66773222 | 2.04E-20 |
| BJK46_009010 | 8138 | 8502 | 10792 | 5588 | 6539 | 5336 | -0.54169459 | 1.57E-15 |
| BJK46_009015 | 6479 | 6540 | 7399 | 4536 | 5338 | 4793 | -0.40543476 | 1.40E-13 |
| BJK46_009020 | 9012 | 9789 | 10156 | 10360 | 8892 | 9909 | 0.165931886 | 0.63165686 |
| BJK46_009025 | 1500 | 1461 | 1952 | 1476 | 1686 | 1446 | -0.1033993 | 0.17805905 |
| BJK46_009030 | 1151 | 1090 | 1354 | 1087 | 1313 | 999 | -0.20118748 | 0.29011594 |
| BJK46_009035 | 1214 | 1180 | 1300 | 1113 | 1303 | 1165 | -0.14780066 | 0.37047296 |
| BJK46_009040 | 275 | 328 | 294 | 397 | 388 | 473 | -0.16836235 | 0.0372126 |
| BJK46_009045 | 1195 | 1384 | 1193 | 1871 | 1841 | 2324 | 0.653944917 | 2.37E-06 |
| BJK46_009050 | 394 | 496 | 359 | 367 | 462 | 495 | -0.48117473 | 0.95445682 |
| BJK46_009055 | 572 | 597 | 389 | 407 | 461 | 635 | -0.54348542 | 0.60351539 |
| BJK46_009060 | 2748 | 3314 | 3182 | 1989 | 2008 | 2151 | -0.56035156 | 7.89E-13 |
| BJK46_009065 | 978 | 1205 | 1188 | 707 | 727 | 761 | -0.83392425 | 2.62E-06 |
| BJK46_009070 | 4628 | 5345 | 5338 | 3245 | 3490 | 3810 | -0.48559198 | 8.64E-13 |
| BJK46_009075 | 1869 | 2179 | 2176 | 1230 | 1271 | 1425 | -0.67512057 | 5.89E-12 |
| BJK46_009080 | 6 | 10 | 10 | 32 | 118 | 64 | 0.762393034 | 6.19E-11 |
| BJK46_009085 | 3670 | 4188 | 4083 | 3491 | 3301 | 3889 | -0.11680941 | 0.01799585 |
| BJK46_009090 | 1124 | 1392 | 1334 | 1164 | 1037 | 1168 | -0.29369048 | 0.07038273 |
| BJK46_009095 | 3270 | 3690 | 3563 | 4575 | 4580 | 4608 | 0.438206652 | 4.87E-05 |
| BJK46_009100 | 698 | 749 | 529 | 614 | 465 | 1015 | -0.34398749 | 0.94395758 |
| BJK46_009105 | 558 | 545 | 561 | 383 | 510 | 486 | -0.77105436 | 0.06504733 |
| BJK46_009120 | 3052 | 3414 | 2783 | 2007 | 1672 | 1881 | -0.71066731 | 8.33E-11 |
| BJK46_009125 | 56 | 45 | 63 | 85 | 135 | 93 | -0.53856347 | 0.00226008 |
| BJK46_009130 | 69 | 49 | 65 | 130 | 248 | 157 | 0.217531708 | 5.53E-07 |
| BJK46_009135 | 10625 | 11591 | 12657 | 15046 | 15769 | 15928 | 0.643842803 | 2.86E-05 |
| BJK46_009140 | 7016 | 7732 | 8920 | 10032 | 10277 | 10122 | 0.510899319 | 0.00014366 |
| BJK46_009145 | 3444 | 3888 | 4366 | 5161 | 5259 | 5147 | 0.474563013 | 3.59E-06 |
| BJK46_009150 | 464 | 753 | 532 | 772 | 759 | 790 | 0.077397009 | 0.07621919 |
| BJK46_009155 | 146045 | 182573 | 125421 | 92823 | 62786 | 85331 | -0.47366296 | 9.80E-08 |
| BJK46_009160 | 126 | 150 | 149 | 81 | 84 | 79 | -2.04938724 | 0.00048361 |
| BJK46_009165 | 3005 | 3250 | 3549 | 2018 | 2209 | 1757 | -0.69037585 | 3.89E-18 |
| BJK46_009170 | 2817 | 3002 | 3381 | 1610 | 1974 | 1534 | -0.82985002 | 1.26E-23 |
| BJK46_009175 | 2397 | 2674 | 3140 | 1360 | 1665 | 1260 | -0.92714515 | 3.98E-23 |
| BJK46_009180 | 2005 | 2020 | 2399 | 1581 | 1773 | 1351 | -0.44866818 | 1.01E-06 |
| BJK46_009185 | 2064 | 2072 | 2617 | 1663 | 1905 | 1382 | -0.44103659 | 5.69E-06 |
| BJK46_009190 | 2129 | 2181 | 2623 | 1390 | 1628 | 1268 | -0.69188606 | 7.84E-14 |
| BJK46_009195 | 2211 | 2174 | 2602 | 900 | 1044 | 879 | -1.34137262 | 5.76E-39 |
| BJK46_009200 | 12807 | 14294 | 16217 | 10864 | 11507 | 11195 | -0.15314753 | 6.86E-07 |
| BJK46_009205 | 8132 | 8558 | 9159 | 2867 | 3048 | 3286 | -1.42713684 | 9.09E-85 |
| BJK46_009210 | 2980 | 3264 | 4134 | 2411 | 2883 | 2490 | -0.37416442 | 2.55E-07 |
| BJK46_009215 | 16987 | 18473 | 21525 | 10483 | 11199 | 10921 | -0.57148305 | 1.74E-19 |
| BJK46_009220 | 1798 | 2145 | 1517 | 2955 | 3718 | 4723 | 1.074802117 | 7.14E-10 |
| BJK46_009225 | 13648 | 15776 | 16939 | 13103 | 13242 | 12856 | -0.01024692 | 0.00125035 |
| BJK46_009230 | 11617 | 12861 | 14798 | 11613 | 12309 | 11067 | 0.0427488 | 0.00907508 |
| BJK46_009235 | 2470 | 2841 | 3178 | 3754 | 3881 | 3169 | 0.384327229 | 0.00148254 |
| BJK46_009240 | 1549 | 1775 | 1853 | 578 | 923 | 700 | -1.38960792 | 1.05E-18 |
| BJK46_009245 | 1121 | 1166 | 1194 | 1527 | 1859 | 1631 | 0.469246476 | 4.91E-05 |
| BJK46_009250 | 3667 | 4952 | 3018 | 6525 | 5003 | 5808 | 0.654808754 | 0.00187743 |
| BJK46_009255 | 3452 | 4217 | 2315 | 3622 | 3152 | 5031 | 0.297487074 | 0.41025261 |
| BJK46_009260 | 16129 | 18247 | 20061 | 58087 | 51801 | 52481 | 1.889162656 | 2.48E-40 |
| BJK46_009265 | 3801 | 4615 | 4045 | 7807 | 8991 | 10354 | 1.197624901 | 2.57E-24 |
| BJK46_009270 | 11609 | 12142 | 12009 | 23206 | 20146 | 23344 | 1.140008265 | 1.41E-13 |
| BJK46_009275 | 6822 | 7869 | 7555 | 15831 | 14558 | 16530 | 1.255134615 | 1.93E-25 |
| BJK46_009280 | 1054 | 1080 | 1103 | 28665 | 24522 | 26857 | 4.691571316 | 2.45E-302 |
| BJK46_009285 | 3379 | 3792 | 3752 | 6179 | 5825 | 6493 | 0.819112264 | 1.69E-16 |
| BJK46_009290 | 23677 | 25307 | 27311 | 17356 | 15587 | 15508 | -0.38851619 | 3.26E-11 |
| BJK46_009295 | 11528 | 12678 | 13777 | 7087 | 7782 | 6913 | -0.64537955 | 4.15E-28 |
| BJK46_009300 | 5184 | 5824 | 6400 | 3861 | 4205 | 3530 | -0.52578358 | 3.91E-17 |
| BJK46_009305 | 17364 | 20008 | 18476 | 15504 | 14683 | 18238 | 0.04096431 | 0.0231609 |
| BJK46_009310 | 3659 | 4219 | 2877 | 14293 | 14174 | 17130 | 2.201412112 | 2.45E-42 |
| BJK46_009315 | 3948 | 4233 | 4475 | 2560 | 2689 | 2862 | -0.60314364 | 3.90E-18 |
| BJK46_009320 | 74089 | 83799 | 101622 | 28119 | 28235 | 24433 | -1.31849045 | 3.06E-49 |
| BJK46_009325 | 29010 | 30418 | 29507 | 7594 | 6590 | 7470 | -1.8103582 | 2.92E-70 |
| BJK46_009330 | 12046 | 13586 | 10586 | 1672 | 1491 | 1665 | -2.84688515 | 8.37E-124 |
| BJK46_009335 | 2843 | 3079 | 3487 | 4071 | 4542 | 3975 | 0.465210918 | 3.07E-06 |
| BJK46_009340 | 841 | 970 | 1081 | 1191 | 1456 | 1097 | 0.227201593 | 0.02629545 |
| BJK46_009345 | 6923 | 7166 | 7840 | 4216 | 3976 | 4115 | -0.76324899 | 2.35E-28 |
| BJK46_009350 | 4029 | 4732 | 3917 | 2848 | 2326 | 2822 | -0.62826211 | 1.51E-09 |
| BJK46_009355 | 51951 | 53873 | 65298 | 76579 | 75018 | 66766 | 0.745248518 | 0.00839893 |
| BJK46_009360 | 1211 | 1313 | 1650 | 2772 | 2737 | 2306 | 0.921420791 | 5.37E-15 |
| BJK46_009365 | 2137 | 2328 | 1835 | 1955 | 1899 | 2781 | 0.077194046 | 0.9585462 |
| BJK46_009370 | 6787 | 6714 | 7505 | 4440 | 4364 | 4591 | -0.57743863 | 5.94E-19 |
| BJK46_009375 | 4427 | 5124 | 5411 | 7884 | 7803 | 8141 | 0.758479628 | 3.99E-15 |
| BJK46_009380 | 3154 | 3883 | 4130 | 6409 | 6639 | 5989 | 0.837629366 | 3.69E-19 |
| BJK46_009385 | 2592 | 3105 | 2593 | 2734 | 2745 | 2927 | 0.052469462 | 0.66470295 |
| BJK46_009390 | 29169 | 33942 | 22769 | 10510 | 10600 | 13714 | -1.03940135 | 1.49E-16 |
| BJK46_009395 | 8275 | 8928 | 9399 | 6344 | 5970 | 6350 | -0.40205119 | 8.32E-12 |
| BJK46_009400 | 3373 | 3640 | 2931 | 4753 | 5235 | 5985 | 0.734556512 | 1.09E-07 |
| BJK46_009405 | 5872 | 6685 | 6379 | 7886 | 7491 | 7928 | 0.399048586 | 0.00537646 |
| BJK46_009410 | 1123 | 1154 | 1326 | 467 | 701 | 497 | -1.40444106 | 3.39E-13 |
| BJK46_009415 | 1985 | 2026 | 2067 | 691 | 827 | 620 | -1.62176982 | 2.74E-35 |
| BJK46_009420 | 2319 | 2291 | 2409 | 730 | 988 | 751 | -1.57591514 | 1.32E-41 |
| BJK46_009425 | 1937 | 2172 | 2231 | 918 | 1002 | 817 | -1.26854036 | 2.71E-30 |
| BJK46_009430 | 3109 | 4023 | 3206 | 2473 | 2561 | 3001 | -0.327095 | 0.00019042 |
| BJK46_009435 | 63749 | 79280 | 50210 | 80295 | 68391 | 93089 | 0.736735226 | 0.14614123 |
| BJK46_009440 | 8890 | 9650 | 9369 | 15101 | 14099 | 16718 | 0.913118915 | 1.03E-10 |
| BJK46_009445 | 1400 | 1631 | 1299 | 1034 | 1223 | 1413 | -0.31343982 | 0.03985343 |
| BJK46_009450 | 1384 | 1741 | 1377 | 2398 | 1997 | 2394 | 0.601181835 | 5.10E-05 |
| BJK46_009455 | 991 | 1171 | 996 | 3729 | 3768 | 3906 | 1.869239905 | 2.77E-71 |
| BJK46_009460 | 315 | 413 | 384 | 287 | 411 | 346 | -0.74657319 | 0.44291642 |
| BJK46_009465 | 104 | 93 | 96 | 110 | 144 | 116 | -0.91953542 | 0.30254437 |
| BJK46_009470 | 3418 | 3857 | 3709 | 3227 | 2971 | 3306 | -0.16966424 | 0.00354452 |
| BJK46_009475 | 2320 | 2528 | 2630 | 1883 | 1929 | 1973 | -0.34942634 | 6.56E-07 |
| BJK46_009480 | 547 | 696 | 507 | 747 | 629 | 717 | -0.1149397 | 0.32000052 |
| BJK46_009485 | 27 | 32 | 32 | 93 | 109 | 98 | 0.097832553 | 5.57E-09 |
| BJK46_009490 | 92 | 89 | 93 | 120 | 250 | 154 | -0.31943909 | 0.00233595 |
| BJK46_009495 | 348 | 391 | 411 | 425 | 365 | 296 | -0.72615023 | 0.51083064 |
| BJK46_009500 | 1477 | 1581 | 1543 | 1487 | 1441 | 1611 | -0.04850926 | 0.48101579 |
| BJK46_009505 | 2963 | 3336 | 3350 | 3646 | 3695 | 3664 | 0.231310365 | 0.10235377 |
| BJK46_009510 | 49 | 35 | 62 | 59 | 136 | 96 | -0.56772343 | 0.00452198 |
| BJK46_009515 | 38274 | 41095 | 46258 | 19881 | 19536 | 19389 | -0.79116756 | 1.54E-28 |
| BJK46_009520 | 41076 | 45036 | 50600 | 20207 | 20961 | 21523 | -0.81278638 | 4.05E-30 |
| BJK46_009525 | 3686 | 3824 | 4640 | 3545 | 4169 | 3509 | -0.06448319 | 0.0308571 |
| BJK46_009530 | 2755 | 3005 | 3605 | 2582 | 3196 | 2504 | -0.14659254 | 0.00934348 |
| BJK46_009535 | 2872 | 2956 | 3589 | 2570 | 3031 | 2408 | -0.20367552 | 0.0009624 |
| BJK46_009540 | 1576 | 1728 | 2051 | 1720 | 1928 | 1510 | -0.05451848 | 0.27827282 |
| BJK46_009545 | 23252 | 24954 | 30988 | 8635 | 8577 | 7537 | -1.43880846 | 5.07E-56 |
| BJK46_009550 | 44449 | 49797 | 58528 | 17724 | 17210 | 15033 | -1.30578262 | 1.78E-47 |
| BJK46_009555 | 26407 | 28618 | 36323 | 11483 | 11748 | 9593 | -1.21344345 | 6.87E-39 |
| BJK46_009560 | 25721 | 28793 | 34213 | 11855 | 12664 | 11594 | -1.02889722 | 1.91E-39 |
| BJK46_009565 | 14930 | 17320 | 18414 | 7072 | 6777 | 6624 | -1.12494037 | 1.01E-50 |
| BJK46_009570 | 19270 | 21979 | 23964 | 8208 | 8184 | 7766 | -1.21223971 | 1.15E-51 |
| BJK46_009575 | 28551 | 30454 | 37550 | 11604 | 10280 | 9332 | -1.36890401 | 3.83E-43 |
| BJK46_009580 | 53923 | 58689 | 68987 | 22051 | 21112 | 20155 | -1.19060904 | 6.49E-45 |
| BJK46_009585 | 35089 | 37081 | 45821 | 15291 | 14979 | 14265 | -1.11397723 | 2.05E-40 |
| BJK46_009590 | 22900 | 25209 | 30335 | 10419 | 9778 | 9638 | -1.14299769 | 5.16E-41 |
| BJK46_009595 | 35056 | 37085 | 44512 | 13814 | 13148 | 12426 | -1.28393768 | 1.64E-48 |
| BJK46_009600 | 28213 | 30495 | 34935 | 9283 | 9337 | 9285 | -1.49220122 | 2.34E-69 |
| BJK46_009605 | 33022 | 35472 | 40904 | 10324 | 9712 | 9313 | -1.63450864 | 1.48E-71 |
| BJK46_009610 | 19524 | 21517 | 25199 | 6253 | 5901 | 5262 | -1.7357358 | 2.17E-76 |
| BJK46_009615 | 19768 | 22051 | 24056 | 6526 | 6139 | 5895 | -1.63109831 | 9.49E-78 |
| BJK46_009620 | 35467 | 37957 | 42227 | 10930 | 10412 | 9741 | -1.62870461 | 8.67E-73 |
| BJK46_009625 | 25352 | 26859 | 30306 | 7534 | 7637 | 7090 | -1.65986553 | 1.11E-84 |
| BJK46_009630 | 25722 | 27093 | 30788 | 7693 | 7539 | 7110 | -1.67227659 | 8.06E-82 |
| BJK46_009635 | 27275 | 29331 | 29900 | 8336 | 7760 | 7701 | -1.62691041 | 2.55E-73 |
| BJK46_009640 | 24625 | 26802 | 27198 | 7824 | 7072 | 6916 | -1.62837893 | 1.73E-69 |
| BJK46_009645 | 31135 | 31973 | 36392 | 9697 | 9204 | 8432 | -1.6120783 | 5.73E-69 |
| BJK46_009650 | 41875 | 41933 | 49738 | 12658 | 12826 | 11645 | -1.56331136 | 3.11E-69 |
| BJK46_009655 | 28296 | 29151 | 32882 | 8618 | 8519 | 8144 | -1.5929942 | 1.50E-76 |
| BJK46_009660 | 27164 | 27530 | 31057 | 8438 | 8375 | 8450 | -1.52213104 | 9.91E-72 |
| BJK46_009665 | 53560 | 55090 | 65471 | 16791 | 15775 | 14905 | -1.56559923 | 5.69E-64 |
| BJK46_009670 | 21045 | 22638 | 26930 | 6496 | 6204 | 5919 | -1.71660989 | 4.96E-78 |
| BJK46_009675 | 35221 | 38155 | 43113 | 11090 | 10518 | 10765 | -1.57565166 | 2.83E-70 |
| BJK46_009680 | 40314 | 43113 | 47988 | 12953 | 13174 | 12596 | -1.47814185 | 5.25E-69 |
| BJK46_009685 | 27056 | 30505 | 31252 | 9544 | 9540 | 9097 | -1.40707141 | 4.50E-65 |
| BJK46_009690 | 2163 | 2225 | 2344 | 5460 | 5462 | 5902 | 1.362426092 | 4.36E-51 |
| BJK46_009695 | 159 | 182 | 174 | 115 | 276 | 181 | -0.93789707 | 0.81037111 |
| BJK46_009700 | 19019 | 19769 | 19723 | 4530 | 4742 | 4688 | -1.9133575 | 1.79E-123 |
| BJK46_009705 | 4487 | 4954 | 5734 | 3568 | 4338 | 3584 | -0.34570527 | 8.93E-09 |
| BJK46_009710 | 2648 | 2919 | 2828 | 6331 | 6296 | 6622 | 1.250192858 | 1.38E-43 |
| BJK46_009715 | 605 | 563 | 752 | 647 | 957 | 660 | -0.12147171 | 0.36026933 |
| BJK46_009720 | 4442 | 5002 | 4605 | 2396 | 2844 | 3022 | -0.72884419 | 4.40E-19 |
| BJK46_009725 | 3724 | 4028 | 4253 | 2788 | 2993 | 2873 | -0.43196093 | 1.34E-12 |
| BJK46_009730 | 8737 | 8908 | 9317 | 14256 | 12903 | 13210 | 0.763722014 | 2.82E-08 |
| BJK46_009735 | 9137 | 9647 | 10235 | 9800 | 9602 | 9537 | 0.154093899 | 0.43123687 |
| BJK46_009740 | 1118 | 991 | 984 | 752 | 803 | 739 | -0.65903546 | 0.00125355 |
| BJK46_009745 | 4601 | 5085 | 5768 | 6074 | 6457 | 6069 | 0.343683505 | 0.00429566 |
| BJK46_009750 | 3598 | 3971 | 4108 | 5072 | 5314 | 5079 | 0.46274676 | 2.13E-06 |
| BJK46_009755 | 13478 | 15217 | 17346 | 4147 | 4679 | 4139 | -1.6993114 | 1.27E-112 |
| BJK46_009760 | 5596 | 5818 | 6729 | 4571 | 4726 | 4432 | -0.33280545 | 5.09E-10 |
| BJK46_009765 | 3885 | 3987 | 4783 | 3089 | 3671 | 3268 | -0.28827171 | 5.02E-07 |
| BJK46_009770 | 2139 | 2140 | 2625 | 1876 | 2335 | 1926 | -0.15146205 | 0.01323115 |
| BJK46_009775 | 2968 | 3095 | 3297 | 3164 | 3420 | 3262 | 0.108458367 | 0.89977006 |
| BJK46_009780 | 2016 | 2282 | 2378 | 2308 | 2480 | 2519 | 0.15418592 | 0.40693759 |
| BJK46_009785 | 2233 | 2577 | 2759 | 2192 | 2562 | 1996 | -0.14512046 | 0.01262406 |
| BJK46_009790 | 1324 | 1521 | 1580 | 2237 | 2427 | 2149 | 0.629199663 | 7.14E-10 |
| BJK46_009795 | 1276 | 1475 | 1562 | 1193 | 1419 | 1184 | -0.24837131 | 0.03347077 |
| BJK46_009800 | 1830 | 2074 | 2258 | 1616 | 2080 | 1651 | -0.19915989 | 0.00541908 |
| BJK46_009805 | 3320 | 3632 | 3905 | 2477 | 2641 | 2361 | -0.5044743 | 3.04E-14 |
| BJK46_009810 | 3436 | 3513 | 4141 | 2618 | 2882 | 2336 | -0.46816803 | 9.68E-11 |
| BJK46_009815 | 3289 | 3421 | 3747 | 2645 | 2898 | 2631 | -0.32181846 | 4.90E-08 |
| BJK46_009820 | 17522 | 16343 | 14610 | 3959 | 4483 | 3821 | -1.85880595 | 1.26E-74 |
| BJK46_009825 | 11056 | 10664 | 9705 | 2876 | 3251 | 2725 | -1.75993614 | 7.63E-79 |
| BJK46_009830 | 9573 | 9612 | 9004 | 3273 | 3696 | 3031 | -1.42911469 | 1.61E-64 |
| BJK46_009835 | 772 | 795 | 833 | 618 | 986 | 737 | -0.3487693 | 0.52440056 |
| BJK46_009840 | 893 | 935 | 949 | 2079 | 2375 | 2247 | 1.225551446 | 6.47E-31 |
| BJK46_009845 | 3324 | 3655 | 3957 | 2549 | 2760 | 2542 | -0.44263331 | 1.73E-12 |
| BJK46_009850 | 2591 | 2782 | 3324 | 1765 | 2027 | 1782 | -0.6153004 | 4.71E-16 |
| BJK46_009855 | 2504 | 2532 | 2953 | 1847 | 2150 | 1857 | -0.42738743 | 2.95E-09 |
| BJK46_009860 | 1489 | 1598 | 1701 | 1135 | 1403 | 1137 | -0.43947883 | 8.71E-05 |
| BJK46_009865 | 1416 | 1651 | 1539 | 3203 | 3323 | 3042 | 1.075815511 | 8.49E-29 |
| BJK46_009870 | 2179 | 2028 | 2206 | 4169 | 3960 | 3654 | 0.906160578 | 3.17E-17 |
| BJK46_009875 | 27006 | 30924 | 25966 | 2234 | 2129 | 2393 | -3.51233541 | 3.78E-181 |
| BJK46_009880 | 3354 | 3906 | 3495 | 2549 | 2693 | 2784 | -0.3871445 | 4.69E-08 |
| BJK46_009885 | 2225 | 2524 | 2846 | 3231 | 3793 | 3444 | 0.497804971 | 7.28E-07 |
| BJK46_009890 | 1348 | 1437 | 1533 | 2384 | 2826 | 2424 | 0.829615932 | 1.49E-17 |
| BJK46_009895 | 39356 | 40301 | 43774 | 870 | 1206 | 1001 | -5.25113244 | 0 |
| BJK46_009900 | 1576 | 1707 | 1908 | 1680 | 1904 | 1668 | 0.0161299 | 0.63231134 |
| BJK46_009905 | 2397 | 2512 | 3025 | 2908 | 3322 | 2750 | 0.209722195 | 0.17796889 |
| BJK46_009910 | 1716 | 1759 | 2171 | 2306 | 2653 | 2161 | 0.354447194 | 0.00410972 |
| BJK46_009915 | 3799 | 4417 | 4499 | 12548 | 13351 | 13270 | 1.734537752 | 1.09E-85 |
| BJK46_009920 | 1991 | 1999 | 2153 | 1181 | 1361 | 1186 | -0.74066921 | 2.88E-15 |
| BJK46_009925 | 2039 | 2584 | 2730 | 2821 | 2788 | 2658 | 0.205118844 | 0.21728841 |
| BJK46_009930 | 1222 | 1566 | 1961 | 1608 | 2036 | 1529 | 0.125409347 | 0.60567222 |
| BJK46_009935 | 2103 | 2510 | 3246 | 2391 | 2835 | 2325 | -0.01548693 | 0.29337291 |
| BJK46_009940 | 2258 | 2582 | 3389 | 2352 | 2929 | 2427 | -0.05438034 | 0.14687745 |
| BJK46_009945 | 3260 | 3939 | 4921 | 3742 | 4169 | 3747 | 0.010518207 | 0.24524296 |
| BJK46_009950 | 1120 | 1181 | 1335 | 3518 | 3975 | 3685 | 1.639540008 | 1.18E-79 |
| BJK46_009955 | 2030 | 2083 | 2106 | 621 | 1199 | 735 | -1.41526204 | 2.00E-15 |
| BJK46_009960 | 254 | 295 | 317 | 322 | 937 | 505 | 0.288036926 | 0.00120583 |
| BJK46_009965 | 235 | 206 | 232 | 297 | 925 | 466 | 0.491688899 | 8.65E-05 |
| BJK46_009970 | 489 | 438 | 482 | 698 | 1579 | 941 | 0.764534551 | 2.58E-06 |
| BJK46_009975 | 305 | 303 | 368 | 441 | 1014 | 560 | 0.420261218 | 0.00015761 |
| BJK46_009980 | 418 | 498 | 478 | 643 | 1035 | 783 | 0.395666534 | 7.29E-05 |
| BJK46_009985 | 287 | 304 | 331 | 464 | 918 | 587 | 0.485327529 | 8.87E-06 |
| BJK46_009990 | 306 | 287 | 334 | 493 | 1166 | 749 | 0.782010506 | 2.89E-07 |
| BJK46_009995 | 126 | 109 | 123 | 232 | 608 | 318 | 0.653920246 | 1.34E-07 |
| BJK46_010000 | 162 | 165 | 148 | 400 | 923 | 519 | 1.134803378 | 1.11E-11 |
| BJK46_010005 | 115 | 136 | 116 | 307 | 788 | 445 | 1.119969165 | 1.95E-11 |
| BJK46_010010 | 59 | 67 | 60 | 197 | 503 | 247 | 1.081697484 | 3.13E-12 |
| BJK46_010015 | 969 | 974 | 884 | 2642 | 7238 | 3656 | 2.141307831 | 1.71E-19 |
| BJK46_010020 | 157916 | 172903 | 207323 | 107313 | 103508 | 91549 | -0.36441383 | 2.88E-14 |
| BJK46_010025 | 19984 | 22351 | 26345 | 14995 | 13319 | 11918 | -0.5171163 | 2.15E-12 |
| BJK46_010030 | 414 | 472 | 413 | 481 | 568 | 634 | -0.13387042 | 0.11051123 |
| BJK46_010035 | 263 | 335 | 271 | 353 | 421 | 488 | -0.12874857 | 0.02732476 |
| BJK46_010040 | 1340 | 1395 | 1587 | 1111 | 1740 | 1172 | -0.18688718 | 0.22373719 |
| BJK46_010045 | 2704 | 2991 | 3047 | 1554 | 1516 | 1807 | -0.82543854 | 3.09E-19 |
| BJK46_010050 | 732 | 799 | 724 | 717 | 833 | 775 | -0.25563943 | 0.88004477 |
| BJK46_010055 | 177 | 215 | 220 | 297 | 359 | 360 | -0.06740654 | 0.00128404 |
| BJK46_010070 | 1741 | 1982 | 2146 | 1540 | 1632 | 1514 | -0.32192211 | 3.16E-05 |
| BJK46_010075 | 6799 | 7340 | 8581 | 5415 | 6187 | 4926 | -0.37347074 | 3.00E-10 |
| BJK46_010080 | 7033 | 8153 | 8015 | 3307 | 3421 | 3319 | -1.14147603 | 3.54E-63 |
| BJK46_010085 | 4481 | 5033 | 5446 | 2159 | 2314 | 2056 | -1.15650083 | 6.69E-59 |
| BJK46_010090 | 4110 | 4513 | 4920 | 2019 | 2092 | 1903 | -1.13659245 | 3.78E-54 |
| BJK46_010095 | 4876 | 5640 | 5752 | 2558 | 2719 | 2488 | -1.01919059 | 3.10E-49 |
| BJK46_010100 | 13808 | 15685 | 13480 | 12529 | 13332 | 15625 | 0.170787387 | 0.31191008 |
| BJK46_010105 | 8639 | 9261 | 9016 | 7875 | 8982 | 9549 | 0.108556541 | 0.29139849 |
| BJK46_010110 | 7926 | 8600 | 10259 | 10545 | 11096 | 9565 | 0.381652938 | 0.06108284 |
| BJK46_010115 | 2991 | 3095 | 3678 | 4185 | 4874 | 4246 | 0.494506313 | 8.54E-07 |
| BJK46_010120 | 2937 | 3268 | 3710 | 4381 | 4568 | 4057 | 0.44167421 | 2.68E-05 |
| BJK46_010125 | 5163 | 5791 | 6213 | 8704 | 8755 | 8034 | 0.670604116 | 8.87E-11 |
| BJK46_010130 | 914 | 1021 | 1100 | 1745 | 2071 | 1867 | 0.849441163 | 1.06E-14 |
| BJK46_010135 | 44972 | 47122 | 54293 | 11627 | 12465 | 11873 | -1.73821581 | 1.59E-89 |
| BJK46_010140 | 11974 | 12725 | 14810 | 9769 | 10003 | 8922 | -0.27160284 | 1.92E-09 |
| BJK46_010145 | 6109 | 6933 | 7905 | 5596 | 6027 | 5546 | -0.19726942 | 4.34E-06 |
| BJK46_010150 | 7191 | 7887 | 8334 | 8942 | 8145 | 8723 | 0.267287777 | 0.34029612 |
| BJK46_010155 | 870 | 823 | 864 | 760 | 1047 | 903 | -0.16458808 | 0.94807081 |
| BJK46_010160 | 794 | 881 | 859 | 680 | 837 | 748 | -0.44393472 | 0.13300209 |
| BJK46_010165 | 241 | 254 | 216 | 147 | 217 | 177 | -1.32877786 | 0.04289056 |
| BJK46_010170 | 3139 | 3138 | 3276 | 4327 | 4506 | 4198 | 0.491958722 | 1.28E-06 |
| BJK46_010175 | 1987 | 2141 | 1989 | 2196 | 2371 | 2661 | 0.256597668 | 0.08585522 |
| BJK46_010180 | 2429 | 2741 | 2614 | 2646 | 3043 | 3413 | 0.247795262 | 0.10142837 |
| BJK46_010185 | 1372 | 1635 | 1642 | 1463 | 1856 | 1990 | 0.173211044 | 0.26994386 |
| BJK46_010190 | 4479 | 4883 | 4827 | 5750 | 5851 | 6384 | 0.407763301 | 0.00054084 |
| BJK46_010195 | 2858 | 2923 | 3065 | 3420 | 3593 | 3880 | 0.335163836 | 0.00507743 |
| BJK46_010200 | 14479 | 16304 | 15006 | 14918 | 13040 | 15472 | 0.153969117 | 0.23800404 |
| BJK46_010205 | 1289 | 1413 | 1618 | 1220 | 1497 | 1269 | -0.17195707 | 0.12264503 |
| BJK46_010210 | 1262 | 1394 | 1745 | 1282 | 1514 | 1214 | -0.18062191 | 0.12178128 |
| BJK46_010215 | 1161 | 1210 | 1441 | 1194 | 1563 | 1119 | -0.06799202 | 0.75859412 |
| BJK46_010220 | 735 | 766 | 806 | 890 | 1171 | 911 | 0.115913439 | 0.04844524 |
| BJK46_010225 | 471 | 489 | 463 | 260 | 366 | 311 | -1.23095935 | 0.00060734 |
| BJK46_010230 | 4609 | 5183 | 5045 | 3115 | 3076 | 3368 | -0.58502387 | 2.71E-16 |
| BJK46_010235 | 3823 | 4242 | 4075 | 5656 | 6040 | 6851 | 0.669911673 | 4.78E-10 |
| BJK46_010240 | 3640 | 4123 | 4377 | 4730 | 5261 | 5306 | 0.391965285 | 0.00027857 |
| BJK46_010245 | 8659 | 9530 | 10453 | 11108 | 12345 | 11469 | 0.46250624 | 0.0036806 |
| BJK46_010250 | 1040 | 1299 | 1234 | 1824 | 1942 | 2238 | 0.723865472 | 4.26E-09 |
| BJK46_010255 | 29266 | 29431 | 34388 | 16701 | 16316 | 16301 | -0.63614763 | 5.48E-21 |
| BJK46_010260 | 78028 | 83118 | 97886 | 46448 | 43232 | 40412 | -0.60623166 | 3.50E-20 |
| BJK46_010265 | 44475 | 48133 | 55688 | 25967 | 24215 | 22482 | -0.70605812 | 5.28E-22 |
| BJK46_010270 | 70082 | 74585 | 85049 | 40652 | 39378 | 34944 | -0.62744498 | 3.25E-21 |
| BJK46_010275 | 27578 | 30159 | 34043 | 17261 | 16294 | 15345 | -0.62949632 | 1.28E-19 |
| BJK46_010280 | 28170 | 29306 | 33949 | 16724 | 15626 | 14594 | -0.68614422 | 5.08E-21 |
| BJK46_010285 | 15141 | 16315 | 18983 | 10259 | 10252 | 9496 | -0.53195765 | 1.97E-18 |
| BJK46_010290 | 29119 | 30998 | 36746 | 20203 | 19824 | 17437 | -0.46393144 | 1.07E-13 |
| BJK46_010295 | 18752 | 20290 | 23205 | 12649 | 12030 | 10746 | -0.57175931 | 1.59E-16 |
| BJK46_010300 | 30867 | 31184 | 36261 | 23071 | 23531 | 20235 | -0.26233752 | 6.69E-09 |
| BJK46_010305 | 18773 | 18108 | 22601 | 13853 | 14807 | 12113 | -0.29845502 | 1.30E-08 |
| BJK46_010310 | 27486 | 28291 | 33872 | 21709 | 21641 | 18087 | -0.25927495 | 5.90E-08 |
| BJK46_010315 | 13864 | 13992 | 16996 | 10478 | 10582 | 9189 | -0.36214955 | 2.92E-11 |
| BJK46_010320 | 1405 | 1488 | 1848 | 1475 | 1851 | 1627 | 0.050738607 | 0.98823454 |
| BJK46_010325 | 2859 | 2834 | 3398 | 3256 | 3756 | 3355 | 0.227245355 | 0.11624513 |
| BJK46_010330 | 9518 | 10407 | 12921 | 4840 | 5331 | 4957 | -1.00029621 | 1.13E-42 |
| BJK46_010335 | 9369 | 10124 | 12413 | 5368 | 5389 | 5020 | -0.89787181 | 1.25E-34 |
| BJK46_010340 | 4493 | 4867 | 5918 | 2486 | 2627 | 2282 | -0.99819645 | 6.74E-38 |
| BJK46_010345 | 68985 | 77899 | 66389 | 32830 | 33032 | 41389 | -0.63583438 | 5.85E-15 |
| BJK46_010350 | 12178 | 12779 | 14560 | 9654 | 10513 | 9180 | -0.23942065 | 4.33E-09 |
| BJK46_010355 | 9703 | 10541 | 12441 | 11304 | 12098 | 10515 | 0.24222141 | 0.95214925 |
| BJK46_010360 | 7701 | 8238 | 8631 | 13740 | 13013 | 13113 | 0.871237613 | 1.16E-13 |
| BJK46_010365 | 16390 | 17558 | 19039 | 28578 | 27707 | 29137 | 0.966982308 | 6.37E-10 |
| BJK46_010370 | 26395 | 27729 | 30522 | 28209 | 28098 | 27770 | 0.290622653 | 0.4929757 |
| BJK46_010375 | 511 | 539 | 570 | 1080 | 1258 | 1138 | 0.816977522 | 1.66E-12 |
| BJK46_010380 | 11419 | 12400 | 10888 | 11376 | 9700 | 10827 | 0.061016899 | 0.09780116 |
| BJK46_010385 | 12894 | 14795 | 16793 | 13017 | 13408 | 12888 | 0.053282613 | 0.010709 |
| BJK46_010390 | 9281 | 10873 | 8795 | 6938 | 6766 | 8585 | -0.2493822 | 0.00024795 |
| BJK46_010395 | 1497 | 1672 | 1617 | 734 | 960 | 805 | -1.06503574 | 8.59E-15 |
| BJK46_010400 | 3762 | 4016 | 4017 | 3518 | 3299 | 3546 | -0.14296204 | 0.00418335 |
| BJK46_010405 | 13542 | 15499 | 17141 | 8635 | 8640 | 8212 | -0.66195995 | 2.35E-25 |
| BJK46_010410 | 46735 | 54227 | 61046 | 21307 | 20708 | 20371 | -1.05262167 | 5.67E-39 |
| BJK46_010415 | 4096 | 4676 | 5473 | 3002 | 3316 | 2832 | -0.58585488 | 5.20E-17 |
| BJK46_010420 | 2661 | 3154 | 3581 | 2122 | 2371 | 2014 | -0.49731402 | 3.63E-11 |
| BJK46_010425 | 27495 | 28206 | 30761 | 9172 | 9216 | 9342 | -1.39476324 | 4.00E-64 |
| BJK46_010430 | 23130 | 23032 | 25467 | 7388 | 8543 | 8568 | -1.32582179 | 4.01E-58 |
| BJK46_010435 | 30076 | 29386 | 34052 | 9645 | 10283 | 9958 | -1.39065691 | 1.80E-63 |
| BJK46_010440 | 9209 | 10106 | 10624 | 6865 | 6587 | 6279 | -0.47688058 | 1.68E-15 |
| BJK46_010445 | 9849 | 10729 | 12506 | 6450 | 7227 | 5972 | -0.61784863 | 5.89E-22 |
| BJK46_010450 | 4893 | 5837 | 5867 | 5585 | 5683 | 5273 | 0.06845241 | 0.40412896 |
| BJK46_010455 | 4244 | 4785 | 5112 | 5091 | 5545 | 4762 | 0.185127709 | 0.40523623 |
| BJK46_010460 | 1051 | 1066 | 1123 | 1694 | 1957 | 1662 | 0.654276865 | 8.47E-09 |
| BJK46_010465 | 7098 | 7126 | 8813 | 40449 | 40404 | 38777 | 2.635292694 | 2.40E-99 |
| BJK46_010470 | 3242 | 3402 | 3859 | 3600 | 3739 | 3535 | 0.095195107 | 0.89822152 |
| BJK46_010475 | 4733 | 5073 | 6027 | 5188 | 5618 | 4795 | 0.047999602 | 0.30478362 |
| BJK46_010480 | 9570 | 10404 | 12262 | 9256 | 9990 | 8509 | -0.04899957 | 0.00128611 |
| BJK46_010485 | 5871 | 6227 | 6921 | 5073 | 5047 | 4683 | -0.28905004 | 3.60E-08 |
| BJK46_010490 | 3526 | 3761 | 4308 | 3884 | 3943 | 3517 | 0.017712001 | 0.26139238 |
| BJK46_010495 | 73655 | 78919 | 91573 | 51467 | 54019 | 49813 | -0.25781485 | 1.05E-11 |
| BJK46_010500 | 48077 | 52884 | 63321 | 32276 | 35303 | 30216 | -0.40064362 | 1.21E-13 |
| BJK46_010505 | 19216 | 21217 | 24911 | 19793 | 19565 | 17503 | 0.071604779 | 0.01477803 |
| BJK46_010510 | 8195 | 9109 | 10630 | 7633 | 7966 | 6797 | -0.18600542 | 1.23E-05 |
| BJK46_010515 | 13689 | 14702 | 17321 | 9652 | 9990 | 8590 | -0.49325782 | 2.14E-16 |
| BJK46_010520 | 11952 | 12861 | 15539 | 8662 | 8899 | 7683 | -0.49554762 | 7.70E-16 |
| BJK46_010525 | 11067 | 12527 | 14903 | 9473 | 10292 | 8674 | -0.24760037 | 5.64E-08 |
| BJK46_010530 | 12421 | 13314 | 15733 | 12786 | 13886 | 11537 | 0.100242328 | 0.05858218 |
| BJK46_010535 | 5047 | 5403 | 6336 | 5848 | 5865 | 5259 | 0.091110637 | 0.6042777 |
| BJK46_010540 | 4542 | 4935 | 5447 | 5497 | 6043 | 5324 | 0.243787427 | 0.11000018 |
| BJK46_010545 | 4190 | 4003 | 3834 | 3856 | 4327 | 4846 | 0.161458045 | 0.65848376 |
| BJK46_010550 | 8169 | 9022 | 9586 | 14269 | 14093 | 14362 | 0.863950565 | 4.39E-13 |
| BJK46_010555 | 3891 | 3986 | 4819 | 7728 | 8265 | 6794 | 0.916709109 | 2.11E-20 |
| BJK46_010560 | 3173 | 3175 | 4094 | 6022 | 6391 | 5656 | 0.856490427 | 7.01E-18 |
| BJK46_010565 | 45928 | 48977 | 55846 | 33293 | 32732 | 30757 | -0.29935893 | 6.22E-11 |
| BJK46_010570 | 14391 | 14478 | 16098 | 31975 | 29808 | 31655 | 1.328498143 | 8.59E-21 |
| BJK46_010575 | 16789 | 18017 | 19586 | 19108 | 19828 | 18597 | 0.341405006 | 0.8313531 |
| BJK46_010580 | 10617 | 11767 | 13357 | 10636 | 11401 | 10568 | 0.061379606 | 0.01930876 |
| BJK46_010585 | 28567 | 33780 | 33907 | 34992 | 33405 | 36022 | 0.436937833 | 0.57537424 |
| BJK46_010590 | 10287 | 11067 | 11858 | 6040 | 5494 | 5877 | -0.80916067 | 5.28E-29 |
| BJK46_010595 | 12918 | 14518 | 14735 | 8025 | 7228 | 7527 | -0.7175137 | 1.77E-24 |
| BJK46_010600 | 962 | 988 | 1175 | 588 | 763 | 508 | -1.0424685 | 1.06E-06 |
| BJK46_010605 | 2302 | 2581 | 2773 | 1429 | 1740 | 1465 | -0.71295281 | 1.06E-18 |
| BJK46_010610 | 1221 | 1296 | 1403 | 816 | 1127 | 879 | -0.62325783 | 8.64E-05 |
| BJK46_010615 | 33426 | 37602 | 34504 | 25133 | 23429 | 27731 | -0.16574744 | 7.16E-06 |
| BJK46_010620 | 3425 | 3913 | 4529 | 4946 | 5388 | 4773 | 0.410891354 | 0.00026167 |
| BJK46_010625 | 84295 | 90240 | 100657 | 32249 | 32680 | 34409 | -1.09525379 | 4.21E-46 |
| BJK46_010630 | 16 | 6 | 10 | 56 | 129 | 72 | 1.01369866 | 4.32E-13 |
| BJK46_010635 | 23 | 13 | 16 | 85 | 217 | 99 | 1.116657282 | 3.45E-13 |
| BJK46_010640 | 81 | 143 | 67 | 130 | 137 | 139 | -0.68088633 | 0.17273722 |
| BJK46_010645 | 7194 | 8165 | 9103 | 9972 | 9613 | 9106 | 0.374471207 | 0.04099393 |
| BJK46_010650 | 5607 | 5856 | 6856 | 6175 | 6756 | 5796 | 0.114316883 | 0.7069834 |
| BJK46_010655 | 4897 | 5260 | 6339 | 6398 | 6839 | 5999 | 0.30354915 | 0.03976533 |
| BJK46_010660 | 7741 | 8818 | 7493 | 6897 | 7149 | 8894 | 0.042481454 | 0.24096969 |
| BJK46_010665 | 9567 | 10375 | 10445 | 10088 | 9958 | 10852 | 0.191798745 | 0.66975011 |
| BJK46_010670 | 5636 | 6349 | 6296 | 6093 | 6640 | 6794 | 0.178100785 | 0.66618602 |
| BJK46_010675 | 5709 | 6400 | 6761 | 7150 | 8163 | 7971 | 0.401804221 | 0.00133543 |
| BJK46_010680 | 10312 | 11002 | 10120 | 10366 | 9645 | 10286 | 0.116123943 | 0.22663642 |
| BJK46_010685 | 2780 | 3097 | 3297 | 3019 | 3186 | 3040 | 0.046810524 | 0.52649252 |
| BJK46_010690 | 2791 | 3131 | 3468 | 5100 | 4871 | 5150 | 0.741004752 | 3.59E-14 |
| BJK46_010695 | 68 | 81 | 79 | 95 | 154 | 150 | -0.52267098 | 0.00593509 |
| BJK46_010700 | 27378 | 31886 | 21862 | 91200 | 100586 | 130179 | 2.356850659 | 1.85E-28 |
| BJK46_010705 | 14828 | 16563 | 17340 | 12040 | 12007 | 12053 | -0.20570884 | 9.19E-08 |
| BJK46_010710 | 24678 | 27532 | 30550 | 16514 | 17537 | 15969 | -0.45072421 | 1.37E-15 |
| BJK46_010715 | 6956 | 8035 | 8962 | 4495 | 4857 | 4323 | -0.72540407 | 4.39E-30 |
| BJK46_010720 | 91455 | 111841 | 66383 | 62594 | 54941 | 83152 | -0.00875779 | 0.01354673 |
| BJK46_010725 | 2272 | 2531 | 2517 | 3367 | 3195 | 3743 | 0.521682593 | 5.40E-06 |
| BJK46_010730 | 7830 | 7949 | 8022 | 7418 | 7691 | 8753 | 0.119666956 | 0.50810435 |
| BJK46_010735 | 1066 | 1232 | 1336 | 747 | 806 | 790 | -0.81867877 | 5.04E-07 |
| BJK46_010740 | 2116 | 2261 | 2537 | 1842 | 2039 | 1877 | -0.24436222 | 0.0001257 |
| BJK46_010745 | 2986 | 3026 | 2936 | 1446 | 1447 | 1627 | -0.96962931 | 3.97E-25 |
| BJK46_010750 | 817 | 845 | 961 | 723 | 927 | 781 | -0.36912197 | 0.24154023 |
| BJK46_010755 | 1282 | 1365 | 1552 | 1037 | 1388 | 1074 | -0.35426407 | 0.00979815 |
| BJK46_010760 | 8612 | 9337 | 9640 | 5596 | 6263 | 6730 | -0.46168664 | 3.51E-14 |
| BJK46_010765 | 17059 | 18632 | 20613 | 8603 | 8901 | 8031 | -0.9300638 | 1.32E-40 |
| BJK46_010770 | 11087 | 11759 | 14133 | 5635 | 6348 | 5092 | -0.9844177 | 2.00E-39 |
| BJK46_010775 | 10483 | 10904 | 13369 | 5834 | 6626 | 5229 | -0.84683747 | 1.22E-29 |
| BJK46_010780 | 6728 | 7412 | 5503 | 5423 | 6134 | 7840 | 0.060789781 | 0.52936961 |
| BJK46_010785 | 11150 | 12284 | 12919 | 10807 | 11020 | 11652 | 0.075833312 | 0.03821488 |
| BJK46_010790 | 2592 | 2841 | 3311 | 705 | 784 | 686 | -2.04204781 | 7.85E-100 |
| BJK46_010795 | 4561 | 4817 | 5703 | 1166 | 1591 | 1196 | -1.91452991 | 1.42E-92 |
| BJK46_010800 | 277 | 280 | 245 | 226 | 410 | 294 | -0.5862258 | 0.59032672 |
| BJK46_010805 | 336 | 383 | 338 | 265 | 604 | 424 | -0.40683422 | 0.41356706 |
| BJK46_010810 | 3765 | 4444 | 4186 | 3003 | 2624 | 2893 | -0.50060189 | 6.95E-10 |
| BJK46_010820 | 2215 | 2209 | 2348 | 1204 | 1333 | 1405 | -0.78653312 | 1.09E-17 |
| BJK46_010825 | 8816 | 9421 | 8302 | 4372 | 4953 | 5806 | -0.72882466 | 1.19E-15 |
| BJK46_010830 | 5181 | 5692 | 5270 | 2715 | 3160 | 3499 | -0.74031949 | 1.81E-18 |
| BJK46_010835 | 99 | 123 | 146 | 204 | 373 | 218 | 0.071239468 | 7.65E-05 |
| BJK46_010840 | 43 | 49 | 45 | 126 | 314 | 155 | 0.653753798 | 7.21E-10 |
| BJK46_010845 | 9 | 15 | 8 | 34 | 120 | 53 | 0.479374411 | 6.46E-09 |
| BJK46_010850 | 21 | 22 | 11 | 60 | 165 | 96 | 0.723635921 | 2.43E-10 |
| BJK46_010855 | 19 | 22 | 17 | 78 | 211 | 119 | 1.005610525 | 1.63E-13 |
| BJK46_010860 | 12 | 12 | 12 | 56 | 110 | 67 | 0.665783379 | 4.69E-13 |
| BJK46_010865 | 67 | 75 | 60 | 162 | 226 | 200 | 0.30354315 | 1.15E-08 |
| BJK46_010870 | 185 | 184 | 191 | 245 | 339 | 339 | -0.14158573 | 0.0031157 |
| BJK46_010875 | 131 | 152 | 164 | 154 | 245 | 181 | -0.66987814 | 0.20299794 |
| BJK46_010880 | 142 | 182 | 185 | 188 | 255 | 190 | -0.65728112 | 0.27844676 |
| BJK46_010885 | 138 | 192 | 176 | 173 | 256 | 181 | -0.71965677 | 0.39862601 |
| BJK46_010890 | 154 | 176 | 160 | 152 | 242 | 172 | -0.82475779 | 0.58281218 |
| BJK46_010895 | 145 | 133 | 145 | 143 | 177 | 150 | -0.94091058 | 0.74038963 |
| BJK46_010945 | 16361 | 17225 | 20456 | 15329 | 16466 | 15034 | 0.045795068 | 0.00568438 |
| BJK46_010950 | 5990 | 6901 | 8141 | 6209 | 7175 | 5565 | -0.05841145 | 0.01963815 |
| BJK46_010955 | 2689 | 3176 | 3530 | 3042 | 3347 | 2694 | -0.01290577 | 0.23168978 |
| BJK46_010960 | 1752 | 1946 | 2165 | 2335 | 2660 | 2077 | 0.286532482 | 0.02811067 |
| BJK46_010965 | 1472 | 1537 | 1943 | 3001 | 3389 | 2721 | 0.90430125 | 5.35E-17 |
| BJK46_010970 | 23664 | 26560 | 30623 | 26435 | 26327 | 25937 | 0.261082076 | 0.3610186 |
| BJK46_010975 | 3692 | 4059 | 4407 | 5852 | 6351 | 5979 | 0.644773113 | 1.01E-13 |
| BJK46_010980 | 80985 | 85972 | 82898 | 92627 | 83045 | 95751 | 0.53795366 | 0.62157322 |
| BJK46_010985 | 6009 | 6333 | 6989 | 8420 | 8406 | 8089 | 0.473921498 | 9.44E-05 |
| BJK46_010990 | 6498 | 6839 | 7608 | 7749 | 8123 | 7394 | 0.25984399 | 0.24063933 |
| BJK46_010995 | 14168 | 16238 | 14343 | 9984 | 10204 | 10943 | -0.31665112 | 2.67E-09 |
| BJK46_011000 | 4895 | 5046 | 5413 | 7130 | 8249 | 7980 | 0.686543839 | 2.80E-13 |
| BJK46_011005 | 3010 | 3006 | 3594 | 4394 | 5014 | 4430 | 0.574832118 | 3.17E-09 |
| BJK46_011010 | 6345 | 6943 | 7775 | 8374 | 9276 | 7906 | 0.395335163 | 0.00508574 |
| BJK46_011015 | 4727 | 4852 | 5632 | 4293 | 5280 | 4024 | -0.10809641 | 0.00739718 |
| BJK46_011020 | 10195 | 10843 | 12273 | 8072 | 9543 | 7774 | -0.23653204 | 4.35E-08 |
| BJK46_011025 | 1953 | 2266 | 2265 | 2080 | 2370 | 2161 | 0.048164634 | 0.68321591 |
| BJK46_011030 | 64144 | 67998 | 66819 | 37515 | 35794 | 41512 | -0.43411268 | 2.49E-13 |
| BJK46_011035 | 11524 | 12390 | 14055 | 10552 | 10737 | 10042 | -0.08249159 | 7.82E-05 |
| BJK46_011040 | 10956 | 12122 | 14039 | 10124 | 11413 | 9482 | -0.0687841 | 0.00024555 |
| BJK46_011045 | 3916 | 4454 | 4538 | 4843 | 4399 | 4162 | 0.10883271 | 0.97131399 |
| BJK46_011050 | 7865 | 8569 | 9390 | 8887 | 9006 | 8640 | 0.179339092 | 0.79652114 |
| BJK46_011055 | 8244 | 8868 | 10360 | 9749 | 9885 | 8836 | 0.205559958 | 0.94026883 |
| BJK46_011060 | 5277 | 5519 | 6447 | 6168 | 6667 | 5975 | 0.206034077 | 0.38297338 |
| BJK46_011065 | 91774 | 111460 | 75169 | 46761 | 51304 | 85811 | -0.23021878 | 0.00167162 |
| BJK46_011070 | 4856 | 5027 | 6204 | 3880 | 4217 | 3596 | -0.39809285 | 3.51E-10 |
| BJK46_011075 | 3206 | 3385 | 4050 | 2701 | 2991 | 2496 | -0.3415671 | 2.64E-07 |
| BJK46_011080 | 3499 | 3515 | 4463 | 2775 | 3364 | 2740 | -0.32942717 | 1.67E-06 |
| BJK46_011085 | 19655 | 21696 | 24126 | 17370 | 18297 | 16599 | -0.05890268 | 7.25E-05 |
| BJK46_011090 | 2835 | 2940 | 3589 | 1658 | 2284 | 1827 | -0.68077476 | 1.27E-14 |
| BJK46_011095 | 1169 | 1238 | 1400 | 685 | 959 | 743 | -0.8634377 | 3.43E-07 |
| BJK46_011100 | 2100 | 2242 | 2627 | 1461 | 1844 | 1405 | -0.55973608 | 4.26E-10 |
| BJK46_011105 | 1678 | 1790 | 1903 | 1175 | 1338 | 1175 | -0.57700588 | 7.25E-09 |
| BJK46_011110 | 2799 | 3057 | 3575 | 2151 | 2611 | 2071 | -0.43856507 | 4.11E-09 |
| BJK46_011115 | 3034 | 3363 | 4042 | 2355 | 2785 | 2416 | -0.4292791 | 9.04E-10 |
| BJK46_001735 | 19 | 27 | 21 | 64 | 247 | 104 | 0.747186659 | 1.92E-09 |
| BJK46_001740 | 11558 | 11637 | 11869 | 5090 | 4784 | 5409 | -1.08514134 | 3.75E-41 |
| BJK46_001745 | 18752 | 20339 | 22110 | 30860 | 30872 | 30521 | 0.88148769 | 1.06E-07 |
| BJK46_001750 | 11038 | 11513 | 13793 | 13015 | 14181 | 12049 | 0.321196373 | 0.57049682 |
| BJK46_001755 | 7350 | 8029 | 9463 | 9674 | 10985 | 9047 | 0.404546618 | 0.01936651 |
| BJK46_001760 | 10435 | 11892 | 9865 | 11864 | 11401 | 13941 | 0.398507868 | 0.21570122 |
| BJK46_001765 | 5139 | 6058 | 6077 | 6929 | 6619 | 7043 | 0.33942944 | 0.01612901 |
| BJK46_001770 | 24578 | 26674 | 30154 | 39469 | 40090 | 36918 | 0.833552634 | 8.08E-06 |
| BJK46_001775 | 321 | 359 | 414 | 357 | 666 | 676 | 0.036568073 | 0.01530712 |
| BJK46_001780 | 571 | 607 | 683 | 649 | 1051 | 1066 | 0.231086055 | 0.00763144 |
| BJK46_001785 | 3125 | 3163 | 3278 | 2209 | 2548 | 2017 | -0.47767895 | 3.71E-10 |
| BJK46_001790 | 51253 | 54588 | 63868 | 41790 | 42581 | 38657 | -0.10300439 | 1.03E-06 |
| BJK46_001795 | 38333 | 40203 | 50058 | 16242 | 17088 | 14398 | -1.13365754 | 5.65E-40 |
| BJK46_001800 | 113172 | 123797 | 155273 | 47656 | 47882 | 40898 | -1.10640022 | 2.75E-39 |
| BJK46_001805 | 5511 | 7654 | 6566 | 8352 | 7312 | 9261 | 0.449348197 | 0.0170262 |
| BJK46_001810 | 7214 | 7599 | 8062 | 6549 | 6617 | 7889 | -0.01844626 | 0.05389695 |
| BJK46_001815 | 246192 | 303703 | 176934 | 102962 | 72505 | 147556 | -0.7213424 | 5.64E-07 |
| BJK46_001820 | 159 | 150 | 138 | 179 | 309 | 200 | -0.40026671 | 0.03388553 |
| BJK46_001825 | 589 | 567 | 636 | 529 | 881 | 641 | -0.22052435 | 0.53701353 |
| BJK46_001830 | 5490 | 5697 | 6035 | 4902 | 5312 | 5245 | -0.08765403 | 0.00247933 |
| BJK46_001835 | 12470 | 13096 | 14355 | 11736 | 12159 | 12012 | 0.057011379 | 0.01133316 |
| BJK46_001840 | 1260 | 1341 | 1418 | 1339 | 1443 | 1410 | 0.002897546 | 0.99027521 |
| BJK46_001845 | 24214 | 24483 | 25137 | 33219 | 28592 | 30959 | 0.622658151 | 0.01995613 |
| BJK46_001850 | 7508 | 7297 | 7714 | 9705 | 8737 | 9058 | 0.414638332 | 0.01555867 |
| BJK46_001855 | 5928 | 5786 | 6357 | 7729 | 6963 | 7348 | 0.375675153 | 0.01085677 |
| BJK46_001860 | 16514 | 16316 | 18277 | 21371 | 19106 | 19422 | 0.486082157 | 0.11373045 |
| BJK46_001865 | 6204 | 6238 | 7017 | 7821 | 7144 | 7344 | 0.295037137 | 0.1097108 |
| BJK46_001870 | 4565 | 4457 | 5076 | 5690 | 5777 | 5814 | 0.360373859 | 0.00249032 |
| BJK46_001875 | 5420 | 5438 | 5880 | 6891 | 6981 | 7453 | 0.432088092 | 0.00030112 |
| BJK46_001880 | 2796 | 2954 | 3392 | 2414 | 2527 | 2449 | -0.27499263 | 6.59E-06 |
| BJK46_001885 | 5210 | 5485 | 5981 | 3065 | 3278 | 3137 | -0.76124191 | 1.36E-32 |
| BJK46_001890 | 7336 | 7905 | 9054 | 4650 | 5282 | 4641 | -0.65267265 | 3.11E-27 |
| BJK46_001895 | 21401 | 24508 | 26545 | 15824 | 16204 | 14824 | -0.3624232 | 4.31E-12 |
| BJK46_001900 | 4340 | 4547 | 4749 | 6318 | 6317 | 6445 | 0.552557685 | 2.49E-08 |
| BJK46_001905 | 1711 | 2072 | 2143 | 2894 | 3032 | 3001 | 0.620641069 | 3.05E-10 |
| BJK46_001910 | 8160 | 9144 | 9993 | 10738 | 12131 | 11103 | 0.484693217 | 0.00099534 |
| BJK46_001915 | 1399 | 1625 | 1239 | 1679 | 1890 | 2068 | 0.392929891 | 0.01221837 |
| BJK46_001920 | 11824 | 12392 | 13502 | 16631 | 17002 | 18855 | 0.708803981 | 3.15E-05 |
| BJK46_001925 | 5628 | 5506 | 6484 | 5567 | 5701 | 5113 | -0.0317304 | 0.03680648 |
| BJK46_001930 | 5018 | 5096 | 5841 | 4604 | 5052 | 4679 | -0.08991495 | 0.00254531 |
| BJK46_001935 | 1900 | 2137 | 2319 | 2139 | 2384 | 2156 | 0.093207445 | 0.90258695 |
| BJK46_001940 | 1667 | 1853 | 1590 | 2295 | 2670 | 2813 | 0.620357113 | 5.30E-07 |
| BJK46_001945 | 8429 | 8652 | 9125 | 8136 | 7904 | 7887 | -0.00208046 | 0.01914736 |
| BJK46_001950 | 16110 | 16747 | 18555 | 16619 | 17043 | 15538 | 0.18507094 | 0.19365337 |
| BJK46_001955 | 3846 | 3986 | 4460 | 8077 | 8343 | 7683 | 1.045562202 | 6.34E-34 |
| BJK46_001960 | 2565 | 2669 | 2946 | 5126 | 5579 | 5185 | 1.004993777 | 2.03E-33 |
| BJK46_001965 | 2302 | 2465 | 2864 | 3892 | 4063 | 3754 | 0.656630431 | 2.76E-12 |
| BJK46_001970 | 1044 | 1083 | 1229 | 718 | 1034 | 748 | -0.63765121 | 0.00130699 |
| BJK46_001975 | 787 | 724 | 958 | 1083 | 1264 | 1071 | 0.280638223 | 0.00577416 |
| BJK46_001980 | 5813 | 6631 | 6911 | 7315 | 7299 | 6830 | 0.243725631 | 0.2454239 |
| BJK46_001985 | 252 | 279 | 308 | 172 | 280 | 160 | -1.34851151 | 0.02711385 |
| BJK46_001990 | 26898 | 28047 | 32741 | 20208 | 19698 | 17131 | -0.33874398 | 4.09E-10 |
| BJK46_001995 | 9506 | 10547 | 12070 | 7661 | 7955 | 6783 | -0.37549025 | 1.65E-11 |
| BJK46_002000 | 16202 | 17417 | 20833 | 11966 | 12489 | 10289 | -0.41634871 | 1.40E-11 |
| BJK46_002005 | 14057 | 14719 | 18283 | 9177 | 9379 | 7753 | -0.64072486 | 3.70E-19 |
| BJK46_002010 | 9032 | 9656 | 11436 | 6221 | 6665 | 5917 | -0.55537694 | 8.07E-20 |
| BJK46_002015 | 4271 | 4425 | 4756 | 7164 | 6702 | 6319 | 0.653629485 | 3.35E-10 |
| BJK46_002020 | 891 | 1022 | 926 | 736 | 866 | 807 | -0.47597273 | 0.03934025 |
| BJK46_002025 | 1245 | 1383 | 1371 | 709 | 846 | 835 | -0.91200691 | 3.20E-09 |
| BJK46_002030 | 875 | 939 | 950 | 482 | 645 | 528 | -1.07978664 | 5.87E-07 |
| BJK46_002035 | 957 | 1066 | 1130 | 504 | 759 | 572 | -1.07797418 | 1.88E-07 |
| BJK46_002040 | 5205 | 5681 | 6271 | 8261 | 8644 | 8704 | 0.679095809 | 9.46E-12 |
| BJK46_002045 | 5548 | 5967 | 6664 | 7935 | 8676 | 8589 | 0.575032503 | 6.04E-08 |
| BJK46_002050 | 91442 | 97305 | 77700 | 57801 | 65698 | 80009 | 0.013106047 | 0.0017267 |
| BJK46_002055 | 12329 | 13050 | 12738 | 8418 | 8577 | 9007 | -0.37889147 | 1.86E-12 |
| BJK46_002060 | 4739 | 5628 | 4152 | 3521 | 3681 | 4111 | -0.29921068 | 0.00032528 |
| BJK46_002065 | 1081 | 1398 | 1357 | 945 | 1148 | 1090 | -0.38158099 | 0.01252415 |
| BJK46_002070 | 667 | 810 | 871 | 424 | 735 | 522 | -0.88039839 | 0.00448546 |
| BJK46_002075 | 556 | 640 | 685 | 401 | 793 | 432 | -0.70892595 | 0.20702197 |
| BJK46_002080 | 4828 | 5181 | 4983 | 2606 | 2859 | 3041 | -0.7746922 | 4.97E-25 |
| BJK46_002085 | 320 | 415 | 478 | 207 | 398 | 249 | -1.22722114 | 0.01716248 |
| BJK46_002090 | 351 | 389 | 417 | 174 | 340 | 245 | -1.37805061 | 0.00333468 |
| BJK46_002095 | 11950 | 14073 | 12813 | 11368 | 11215 | 13820 | 0.109237358 | 0.1593257 |
| BJK46_002100 | 173 | 203 | 213 | 322 | 409 | 373 | 0.116227151 | 4.17E-05 |
| BJK46_002105 | 1120 | 1248 | 1136 | 2143 | 2342 | 2486 | 0.979879186 | 1.95E-18 |
| BJK46_002110 | 549 | 596 | 513 | 628 | 689 | 759 | -0.0727445 | 0.15516941 |
| BJK46_002115 | 456 | 529 | 441 | 512 | 623 | 738 | -0.07417491 | 0.10264039 |
| BJK46_002120 | 260 | 358 | 337 | 360 | 413 | 423 | -0.31804357 | 0.18570922 |
| BJK46_002125 | 1360 | 1768 | 1644 | 2249 | 2259 | 2405 | 0.552557616 | 2.83E-06 |
| BJK46_002130 | 131 | 134 | 109 | 656 | 693 | 824 | 1.800495945 | 6.51E-29 |
| BJK46_002140 | 3175 | 3506 | 4035 | 3101 | 3390 | 3136 | -0.11041697 | 0.00660193 |
| BJK46_002145 | 10305 | 11077 | 13235 | 10340 | 10928 | 9250 | 0.00153407 | 0.00693039 |
| BJK46_002150 | 2868 | 3144 | 3691 | 3253 | 3729 | 3491 | 0.153601118 | 0.52885428 |
| BJK46_002155 | 3965 | 5587 | 3889 | 4051 | 3188 | 3982 | -0.19829448 | 0.02976398 |
| BJK46_002160 | 6103 | 6409 | 6810 | 6084 | 6334 | 6936 | 0.086836868 | 0.46202748 |
| BJK46_002165 | 34634 | 37388 | 44054 | 37371 | 36652 | 32754 | 0.21131954 | 0.10569459 |
| BJK46_002170 | 15730 | 15726 | 20148 | 22583 | 22920 | 19158 | 0.591596872 | 0.01618096 |
| BJK46_002175 | 7696 | 7557 | 9773 | 11143 | 11664 | 9931 | 0.547802908 | 0.00043074 |
| BJK46_002180 | 10991 | 11386 | 14619 | 16587 | 17232 | 13968 | 0.59892141 | 0.00379651 |
| BJK46_002185 | 23589 | 25523 | 30999 | 38087 | 37936 | 33366 | 0.764504798 | 0.00035836 |
| BJK46_002190 | 16432 | 18987 | 23244 | 24062 | 25535 | 23336 | 0.600356401 | 0.01501132 |
| BJK46_002195 | 945 | 951 | 892 | 1977 | 1884 | 1837 | 0.962748415 | 2.03E-14 |
| BJK46_002200 | 1254 | 1240 | 1397 | 1044 | 1453 | 1403 | -0.08731769 | 0.62806259 |
| BJK46_002205 | 1526 | 1629 | 1765 | 1289 | 1588 | 1491 | -0.19975818 | 0.02393174 |
| BJK46_002210 | 881 | 943 | 1066 | 1772 | 2125 | 1769 | 0.905799623 | 1.45E-15 |
| BJK46_002215 | 9562 | 10722 | 10368 | 23152 | 26915 | 29566 | 1.616627612 | 5.80E-33 |
| BJK46_002225 | 83 | 89 | 91 | 135 | 182 | 147 | -0.41032833 | 0.00239537 |
| BJK46_002230 | 4998 | 5735 | 5757 | 9188 | 9007 | 8972 | 0.822393789 | 2.24E-16 |
| BJK46_002235 | 4091 | 4623 | 5277 | 7647 | 8095 | 7134 | 0.790961249 | 7.88E-17 |
| BJK46_002240 | 3602 | 3792 | 4425 | 7070 | 7539 | 6791 | 0.926457073 | 3.11E-26 |
| BJK46_002245 | 16197 | 17260 | 17289 | 42899 | 42627 | 40878 | 1.609571948 | 7.70E-33 |
| BJK46_002250 | 7047 | 7574 | 8445 | 14511 | 14614 | 14006 | 1.079918461 | 1.09E-24 |
| BJK46_002255 | 3218 | 3425 | 3885 | 7096 | 6976 | 6463 | 1.027712431 | 8.80E-31 |
| BJK46_002260 | 4163 | 4365 | 5007 | 9527 | 9772 | 8765 | 1.140876253 | 1.08E-36 |
| BJK46_002265 | 4804 | 5104 | 5820 | 11196 | 12043 | 10558 | 1.21960979 | 2.41E-39 |
| BJK46_002270 | 4168 | 4231 | 4587 | 5116 | 5455 | 5406 | 0.359276295 | 0.00126758 |
| BJK46_002275 | 14395 | 14934 | 16706 | 16771 | 17921 | 16057 | 0.384738639 | 0.4048688 |
| BJK46_002280 | 5843 | 6379 | 7129 | 7741 | 8295 | 7573 | 0.391279578 | 0.00251928 |
| BJK46_002285 | 1981 | 2240 | 2396 | 2390 | 2685 | 2733 | 0.263182684 | 0.03487119 |
| BJK46_002290 | 3383 | 3660 | 4087 | 3951 | 4270 | 4210 | 0.210700028 | 0.18374003 |
| BJK46_002295 | 15630 | 15800 | 19360 | 9920 | 10605 | 9771 | -0.52611592 | 1.39E-18 |
| BJK46_002300 | 20939 | 22021 | 26171 | 13300 | 13564 | 12399 | -0.55979052 | 2.61E-18 |
| BJK46_002305 | 48587 | 53632 | 47437 | 34753 | 34203 | 43403 | -0.07647368 | 0.00029125 |
| BJK46_002310 | 324 | 277 | 339 | 245 | 428 | 286 | -0.72015018 | 0.83300542 |
| BJK46_002315 | 542 | 536 | 581 | 424 | 849 | 528 | -0.37964363 | 0.87021351 |
| BJK46_002320 | 8644 | 9554 | 10223 | 7208 | 7317 | 7033 | -0.26918065 | 3.46E-09 |
| BJK46_002325 | 1444 | 1600 | 1752 | 1759 | 1880 | 1821 | 0.185818493 | 0.17999663 |
| BJK46_002330 | 10393 | 11487 | 11584 | 16532 | 15665 | 18105 | 0.807720904 | 1.56E-07 |
| BJK46_002335 | 4617 | 4976 | 4856 | 3802 | 3818 | 3921 | -0.27033079 | 1.64E-06 |
| BJK46_002340 | 1347 | 1409 | 1583 | 2746 | 3187 | 2604 | 0.988342354 | 6.47E-24 |
| BJK46_002345 | 1836 | 1937 | 2232 | 2999 | 3317 | 2816 | 0.629852235 | 1.98E-10 |
| BJK46_002350 | 9094 | 9429 | 10474 | 11237 | 11975 | 11107 | 0.418241056 | 0.02096381 |
| BJK46_002355 | 3875 | 4482 | 4654 | 4396 | 4924 | 4677 | 0.16396706 | 0.5421233 |
| BJK46_002360 | 848 | 1115 | 999 | 1633 | 1485 | 1561 | 0.574113883 | 9.19E-06 |
| BJK46_002365 | 778 | 892 | 767 | 2542 | 2751 | 2690 | 1.677475259 | 2.19E-52 |
| BJK46_002370 | 435 | 507 | 583 | 559 | 765 | 640 | -0.06667994 | 0.09121668 |
| BJK46_002375 | 5324 | 5927 | 6447 | 8492 | 9534 | 8983 | 0.71705072 | 1.67E-13 |
| BJK46_002380 | 3959 | 4411 | 4718 | 5685 | 6678 | 6470 | 0.590020861 | 2.79E-10 |
| BJK46_002385 | 13501 | 15943 | 18963 | 17116 | 16964 | 15692 | 0.296821333 | 0.90278247 |
| BJK46_002390 | 27830 | 31563 | 37490 | 35806 | 37517 | 34346 | 0.479570476 | 0.36230375 |
| BJK46_002395 | 6493 | 6795 | 8247 | 7822 | 8713 | 8278 | 0.324758454 | 0.06594219 |
| BJK46_002400 | 2061 | 2264 | 2653 | 1549 | 1634 | 1497 | -0.55964213 | 6.49E-12 |
| BJK46_002405 | 2025 | 2144 | 2551 | 1278 | 1453 | 1356 | -0.7141845 | 1.22E-16 |
| BJK46_002410 | 83 | 106 | 75 | 125 | 198 | 161 | -0.34737905 | 0.00274456 |
| BJK46_002415 | 125 | 152 | 126 | 185 | 358 | 252 | -0.04235653 | 0.00050503 |
| BJK46_002420 | 13925 | 15075 | 15928 | 15062 | 13533 | 14588 | 0.174330259 | 0.26768245 |
| BJK46_002425 | 14709 | 16023 | 16570 | 13749 | 12954 | 14387 | 0.028247585 | 0.00998746 |
| BJK46_002430 | 2116 | 2156 | 2199 | 1696 | 2018 | 1775 | -0.22835759 | 0.00078772 |
| BJK46_002435 | 4103 | 4316 | 4134 | 1648 | 1962 | 2023 | -1.13307397 | 2.08E-39 |
| BJK46_002440 | 2601 | 2862 | 3219 | 946 | 1188 | 908 | -1.52243079 | 5.08E-56 |
| BJK46_002445 | 7367 | 7923 | 7909 | 5739 | 5095 | 5365 | -0.433499 | 8.20E-11 |
| BJK46_002450 | 4002 | 4571 | 4844 | 3109 | 3132 | 2923 | -0.50215749 | 6.00E-15 |
| BJK46_002455 | 4816 | 5398 | 5712 | 4111 | 4779 | 4428 | -0.19700262 | 5.97E-06 |
| BJK46_002460 | 4132 | 4340 | 5082 | 4227 | 4815 | 4290 | 0.034852073 | 0.26110679 |
| BJK46_002465 | 3710 | 3990 | 4113 | 3260 | 3803 | 3372 | -0.13675584 | 0.00109992 |
| BJK46_002470 | 26872 | 29848 | 26267 | 118871 | 105990 | 128009 | 2.461349257 | 6.05E-53 |
| BJK46_002475 | 4912 | 5074 | 5693 | 6615 | 6869 | 6209 | 0.404428612 | 0.00032062 |
| BJK46_002480 | 2975 | 3298 | 3689 | 4117 | 4381 | 3911 | 0.364896788 | 0.00083319 |
| BJK46_002485 | 1556 | 1705 | 1907 | 2196 | 2521 | 2130 | 0.419378825 | 0.00012601 |
| BJK46_002490 | 2906 | 3059 | 3492 | 4525 | 5001 | 4327 | 0.598283961 | 1.93E-10 |
| BJK46_002495 | 3414 | 3558 | 4214 | 6418 | 7059 | 6067 | 0.869128208 | 4.06E-22 |
| BJK46_002500 | 1228 | 1258 | 1384 | 2871 | 3317 | 3004 | 1.259865437 | 9.22E-43 |
| BJK46_002505 | 280 | 296 | 356 | 378 | 682 | 424 | 0.015327725 | 0.00794857 |
| BJK46_002510 | 297 | 313 | 394 | 424 | 613 | 438 | -0.04916554 | 0.01546989 |
| BJK46_002515 | 513 | 565 | 694 | 695 | 912 | 670 | -0.00250515 | 0.08808273 |
| BJK46_002520 | 4147 | 4210 | 4550 | 3532 | 3663 | 3844 | -0.17648252 | 0.00029742 |
| BJK46_002525 | 13370 | 14111 | 12252 | 5141 | 4062 | 4405 | -1.43541477 | 1.02E-38 |
| BJK46_002530 | 1807 | 2010 | 2107 | 1209 | 1598 | 1336 | -0.53379402 | 1.44E-08 |
| BJK46_002535 | 13299 | 14603 | 15361 | 15604 | 15913 | 16722 | 0.394727905 | 0.31207372 |
| BJK46_002540 | 2399 | 2561 | 2858 | 3898 | 4377 | 3657 | 0.644726266 | 1.02E-11 |
| BJK46_002545 | 785 | 896 | 1081 | 1300 | 1770 | 1307 | 0.54024084 | 2.46E-05 |
| BJK46_002550 | 499 | 524 | 637 | 962 | 1352 | 1054 | 0.726104813 | 3.73E-09 |
| BJK46_002555 | 5303 | 5657 | 4629 | 4553 | 4678 | 6307 | 0.047761164 | 0.58500831 |
| BJK46_002560 | 3019 | 3365 | 2851 | 1884 | 1987 | 2361 | -0.54627907 | 2.56E-08 |
| BJK46_002565 | 487 | 559 | 630 | 10599 | 7510 | 10561 | 4.105981238 | 1.23E-160 |
| BJK46_002570 | 3066 | 3364 | 3744 | 4237 | 4776 | 4589 | 0.469583876 | 1.46E-06 |
| BJK46_002575 | 6276 | 6626 | 7829 | 8099 | 9245 | 8198 | 0.417999276 | 0.00189336 |
| BJK46_002580 | 1147 | 1226 | 1349 | 1716 | 1929 | 1680 | 0.483002416 | 2.06E-05 |
| BJK46_002585 | 181 | 213 | 179 | 7832 | 7886 | 8365 | 5.300617214 | 0 |
| BJK46_002590 | 956 | 910 | 925 | 7832 | 7872 | 8356 | 3.134048983 | 2.07E-225 |
| BJK46_002595 | 3369 | 3582 | 3620 | 25394 | 23821 | 26046 | 2.982664373 | 9.61E-151 |
| BJK46_002600 | 6051 | 6518 | 7220 | 5948 | 6260 | 5748 | -0.05517073 | 0.00615356 |
| BJK46_002605 | 1341 | 1513 | 1503 | 1879 | 2049 | 1983 | 0.434999494 | 7.77E-05 |
| BJK46_002610 | 2686 | 3004 | 3143 | 2222 | 2056 | 1937 | -0.48261296 | 1.02E-09 |
| BJK46_002615 | 6549 | 6921 | 8208 | 4742 | 5137 | 4691 | -0.49250204 | 5.84E-17 |
| BJK46_002620 | 2006 | 1921 | 2505 | 1739 | 1925 | 1661 | -0.25392558 | 0.00089925 |
| BJK46_002625 | 19053 | 21136 | 20103 | 33662 | 33828 | 37710 | 1.094314623 | 1.26E-11 |
| BJK46_002630 | 304 | 388 | 344 | 1039 | 1016 | 1082 | 1.195106681 | 3.96E-19 |
| BJK46_002635 | 1511 | 1740 | 1789 | 5787 | 5520 | 5679 | 1.788930382 | 1.35E-87 |
| BJK46_002640 | 1460 | 1559 | 1609 | 5896 | 5659 | 5488 | 1.911784447 | 1.79E-100 |
| BJK46_002645 | 6179 | 7201 | 6916 | 42510 | 39836 | 45743 | 2.906935038 | 3.68E-108 |
| BJK46_002650 | 5159 | 5217 | 5625 | 8532 | 9336 | 9795 | 0.886637445 | 5.97E-19 |
| BJK46_002655 | 964 | 907 | 1037 | 1904 | 1986 | 1906 | 0.937754121 | 2.62E-16 |
| BJK46_002660 | 734 | 772 | 827 | 1505 | 1717 | 1172 | 0.742931231 | 2.32E-08 |
| BJK46_002665 | 2081 | 2279 | 2438 | 1835 | 2121 | 1896 | -0.19908398 | 0.00100967 |
| BJK46_002670 | 8780 | 9449 | 8195 | 10906 | 11849 | 13628 | 0.627234484 | 0.00026011 |
| BJK46_002675 | 4937 | 5595 | 4922 | 8455 | 9163 | 11547 | 1.004939102 | 1.87E-13 |
| BJK46_002680 | 253 | 272 | 304 | 403 | 585 | 462 | 0.151537341 | 0.00022169 |
| BJK46_002685 | 3970 | 4367 | 4130 | 3637 | 3720 | 3895 | -0.0991863 | 0.0113181 |
| BJK46_002690 | 1148 | 1195 | 1329 | 1395 | 1533 | 1195 | 0.085319653 | 0.40954398 |
| BJK46_002695 | 2871 | 3029 | 3093 | 3622 | 3776 | 3659 | 0.33580173 | 0.00283194 |
| BJK46_002700 | 1433 | 1573 | 1608 | 1711 | 2181 | 1866 | 0.309545972 | 0.01047502 |
| BJK46_002705 | 6440 | 7333 | 6400 | 13300 | 13990 | 16798 | 1.285114545 | 3.45E-22 |
| BJK46_002710 | 6644 | 6838 | 7668 | 5901 | 5806 | 5864 | -0.17982837 | 2.07E-05 |
| BJK46_002715 | 7489 | 8041 | 8657 | 7190 | 7918 | 7628 | 0.028885583 | 0.04071282 |
| BJK46_002720 | 1169 | 1204 | 1439 | 837 | 1270 | 800 | -0.5576205 | 0.00431403 |
| BJK46_002725 | 657 | 710 | 863 | 526 | 867 | 531 | -0.59565141 | 0.149163 |
| BJK46_002730 | 269 | 272 | 335 | 184 | 292 | 218 | -1.16203282 | 0.06812758 |
| BJK46_002735 | 885 | 865 | 976 | 488 | 688 | 508 | -1.04077835 | 6.15E-06 |
| BJK46_002740 | 2508 | 2845 | 2587 | 3015 | 2794 | 2811 | 0.147286537 | 0.55470593 |
| BJK46_002745 | 23929 | 25670 | 28313 | 13835 | 15179 | 15587 | -0.54010194 | 2.69E-18 |
| BJK46_002750 | 12060 | 12420 | 13929 | 10745 | 10956 | 9790 | -0.09334473 | 6.85E-05 |
| BJK46_002755 | 8326 | 9608 | 9734 | 8835 | 8720 | 8359 | 0.04977441 | 0.05896051 |
| BJK46_002760 | 1262 | 1218 | 1310 | 1230 | 1595 | 1327 | 0.053094205 | 0.62475345 |
| BJK46_002765 | 451 | 495 | 506 | 482 | 763 | 552 | -0.18094364 | 0.22630015 |
| BJK46_002770 | 2260 | 2389 | 2548 | 2802 | 3066 | 2863 | 0.305420502 | 0.00614945 |
| BJK46_002775 | 1410 | 1642 | 1708 | 1823 | 2031 | 1750 | 0.233395153 | 0.06878362 |
| BJK46_002780 | 1600 | 1808 | 1938 | 1834 | 2232 | 1846 | 0.1510829 | 0.38351265 |
| BJK46_002785 | 4036 | 4263 | 4130 | 1765 | 1701 | 1753 | -1.22478977 | 6.58E-48 |
| BJK46_002790 | 4871 | 5583 | 5355 | 6064 | 6243 | 6697 | 0.339232435 | 0.01114395 |
| BJK46_002795 | 5962 | 6390 | 6832 | 3686 | 3394 | 3338 | -0.82142896 | 4.19E-30 |
| BJK46_002800 | 2454 | 2559 | 2708 | 8152 | 7571 | 8531 | 1.707183357 | 3.01E-73 |
| BJK46_002805 | 349 | 361 | 373 | 916 | 1114 | 1041 | 1.095415135 | 3.59E-18 |
| BJK46_011120 | 8132 | 8470 | 8851 | 7976 | 8340 | 8692 | 0.104346684 | 0.27204493 |
| BJK46_011125 | 2263 | 2284 | 2552 | 2734 | 2842 | 2609 | 0.231273767 | 0.08810014 |
| BJK46_011130 | 840 | 904 | 988 | 934 | 940 | 875 | -0.20605199 | 0.72128003 |
| BJK46_011135 | 2430 | 2465 | 2589 | 2505 | 2781 | 2444 | 0.069474197 | 0.82067223 |
| BJK46_011140 | 2723 | 2757 | 2942 | 2760 | 2736 | 2993 | 0.039576705 | 0.57262271 |
| BJK46_011145 | 8932 | 8977 | 10269 | 8071 | 8224 | 7765 | -0.08956507 | 0.00032419 |
| BJK46_011150 | 14210 | 15310 | 16867 | 12972 | 13178 | 12780 | -0.02353193 | 0.00063542 |
| BJK46_011155 | 3943 | 4569 | 4966 | 4036 | 4214 | 4352 | -0.03747003 | 0.04914925 |
| BJK46_011160 | 66961 | 100680 | 58320 | 320822 | 193844 | 328411 | 2.365538529 | 2.96E-15 |
| BJK46_011165 | 13984 | 14921 | 15097 | 6101 | 5876 | 6281 | -1.11638419 | 1.65E-51 |
| BJK46_011170 | 5819 | 5991 | 6319 | 1822 | 1905 | 1726 | -1.69615391 | 4.37E-113 |
| BJK46_011175 | 9206 | 9466 | 9360 | 3234 | 3052 | 3191 | -1.49688167 | 5.81E-78 |
| BJK46_011180 | 85201 | 84716 | 79672 | 30379 | 24268 | 29848 | -1.21264292 | 3.97E-31 |
| BJK46_011185 | 1389 | 1446 | 1626 | 931 | 1123 | 948 | -0.67245344 | 2.24E-07 |
| BJK46_011190 | 1706 | 1798 | 2137 | 1078 | 1619 | 1159 | -0.59083983 | 1.12E-06 |
| BJK46_011195 | 741 | 847 | 958 | 595 | 874 | 628 | -0.59161123 | 0.04037757 |
| BJK46_011200 | 6821 | 7563 | 8712 | 2455 | 2726 | 2393 | -1.54886872 | 1.65E-106 |
| BJK46_011205 | 3351 | 3679 | 3752 | 4744 | 4607 | 4935 | 0.458033579 | 2.36E-05 |
| BJK46_011210 | 15797 | 19960 | 18065 | 6447 | 5800 | 7534 | -1.26660035 | 5.39E-36 |
| BJK46_011215 | 8390 | 10088 | 10447 | 3237 | 2951 | 3460 | -1.51081936 | 8.38E-68 |
| BJK46_011220 | 12559 | 14262 | 14479 | 4285 | 4206 | 4388 | -1.56450175 | 6.59E-94 |
| BJK46_011225 | 7148 | 7893 | 8157 | 5502 | 5936 | 5437 | -0.36993215 | 3.84E-13 |
| BJK46_011230 | 3123 | 3408 | 4140 | 3821 | 4188 | 3462 | 0.153662415 | 0.59892306 |
| BJK46_011235 | 505 | 528 | 573 | 1162 | 1472 | 1223 | 0.99616415 | 2.41E-16 |
| BJK46_011240 | 2121 | 2362 | 2560 | 2340 | 2670 | 2441 | 0.10614865 | 0.81020875 |
| BJK46_011245 | 4569 | 5188 | 5737 | 8828 | 8403 | 8955 | 0.853419565 | 6.13E-17 |
| BJK46_011250 | 5134 | 5554 | 6689 | 10239 | 10106 | 10297 | 0.940798342 | 1.72E-19 |
| BJK46_011255 | 1432 | 1547 | 1868 | 2986 | 2881 | 2931 | 0.887601211 | 2.35E-17 |
| BJK46_011260 | 2556 | 2789 | 3314 | 5400 | 5600 | 5420 | 0.979856117 | 2.48E-27 |
| BJK46_011265 | 1443 | 1639 | 1770 | 3224 | 3389 | 3333 | 1.061295971 | 2.50E-32 |
| BJK46_011270 | 925 | 1013 | 1187 | 2269 | 2450 | 2369 | 1.166119698 | 1.27E-29 |
| BJK46_011275 | 1133 | 1378 | 1323 | 1833 | 1774 | 1748 | 0.456646975 | 0.00015557 |
| BJK46_011280 | 116 | 115 | 125 | 133 | 131 | 130 | -1.03035201 | 0.73507479 |
| BJK46_011285 | 140 | 140 | 140 | 185 | 220 | 211 | -0.47189365 | 0.03410807 |
| BJK46_011290 | 1173 | 1369 | 1375 | 1887 | 2279 | 2185 | 0.68671446 | 1.89E-10 |
| BJK46_011295 | 160 | 159 | 179 | 197 | 398 | 251 | -0.20275573 | 0.00754711 |
| BJK46_011300 | 12720 | 13521 | 14425 | 15249 | 14716 | 15075 | 0.376011742 | 0.35410399 |
| BJK46_011305 | 10137 | 11715 | 9685 | 14691 | 14741 | 17571 | 0.785256896 | 1.23E-05 |
| BJK46_011310 | 5157 | 5725 | 5885 | 5991 | 5437 | 5580 | 0.092257009 | 0.65776478 |
| BJK46_011315 | 2134 | 2468 | 2556 | 2478 | 2197 | 2169 | -0.04150139 | 0.21851422 |
| BJK46_011320 | 4716 | 5215 | 5679 | 13557 | 13789 | 12952 | 1.500380075 | 1.49E-59 |
| BJK46_011325 | 21447 | 22945 | 26385 | 9494 | 10345 | 10043 | -1.00295863 | 4.39E-42 |
| BJK46_011330 | 13722 | 16392 | 11384 | 12956 | 12311 | 16214 | 0.225938563 | 0.65614621 |
| BJK46_011335 | 4213 | 4851 | 5275 | 6693 | 6610 | 6533 | 0.544615386 | 9.42E-08 |
| BJK46_011340 | 7235 | 7683 | 8447 | 10764 | 11169 | 10575 | 0.625729 | 9.14E-08 |
| BJK46_011345 | 18875 | 17974 | 18330 | 10257 | 9648 | 12203 | -0.56379168 | 3.98E-12 |
| BJK46_011350 | 6625 | 7560 | 8136 | 17027 | 15941 | 16885 | 1.347827813 | 9.93E-33 |
| BJK46_011355 | 2864 | 3169 | 3564 | 11704 | 11170 | 10965 | 1.89989614 | 1.43E-94 |
| BJK46_011360 | 1727 | 1936 | 2237 | 9504 | 9248 | 8135 | 2.244294577 | 7.95E-124 |
| BJK46_011365 | 1515 | 1723 | 1996 | 5376 | 5208 | 4745 | 1.588269892 | 6.49E-60 |
| BJK46_011370 | 1864 | 2180 | 2508 | 986 | 1374 | 1069 | -0.95977335 | 4.00E-18 |
| BJK46_011375 | 848 | 926 | 1055 | 437 | 621 | 500 | -1.20985441 | 3.03E-08 |
| BJK46_011380 | 690 | 807 | 642 | 1124 | 1273 | 1537 | 0.677728981 | 1.60E-06 |
| BJK46_011385 | 72515 | 76270 | 87479 | 55625 | 54725 | 47448 | -0.19266713 | 8.39E-09 |
| BJK46_011390 | 53536 | 54874 | 65450 | 39231 | 41179 | 36574 | -0.21285126 | 3.60E-09 |
| BJK46_011395 | 33447 | 33404 | 39803 | 21541 | 22935 | 19198 | -0.44786926 | 6.23E-14 |
| BJK46_011400 | 40561 | 42531 | 48478 | 25421 | 25530 | 21374 | -0.55134609 | 7.24E-17 |
| BJK46_011405 | 3662 | 4282 | 4208 | 3625 | 3700 | 3356 | -0.13825035 | 0.00195912 |
| BJK46_011410 | 7111 | 7737 | 8720 | 11162 | 10629 | 10356 | 0.598535915 | 4.27E-06 |
| BJK46_011415 | 2143 | 2491 | 1727 | 1387 | 1678 | 2057 | -0.30654134 | 0.01550091 |
| BJK46_011420 | 7053 | 7775 | 8729 | 5215 | 5684 | 5745 | -0.40902618 | 7.69E-14 |
| BJK46_011425 | 33076 | 35400 | 40621 | 24214 | 24503 | 23599 | -0.28516401 | 1.73E-10 |
| BJK46_011430 | 4704 | 5638 | 5076 | 4577 | 4841 | 5483 | 0.013020398 | 0.22570179 |
| BJK46_011435 | 2962 | 3649 | 3909 | 2812 | 2957 | 2745 | -0.26233111 | 1.49E-05 |
| BJK46_011440 | 2996 | 3808 | 4021 | 3218 | 3332 | 3178 | -0.10409091 | 0.01483724 |
| BJK46_011445 | 2555 | 3211 | 2814 | 3786 | 3811 | 3734 | 0.443911075 | 0.00019298 |
| BJK46_011450 | 5339 | 5714 | 6091 | 6895 | 7549 | 6736 | 0.388748906 | 0.00071947 |
| BJK46_011455 | 3854 | 4544 | 3676 | 1526 | 1361 | 1833 | -1.33552597 | 3.71E-27 |
| BJK46_011460 | 16206 | 17281 | 17603 | 33548 | 33691 | 36192 | 1.300636327 | 6.05E-20 |
| BJK46_011465 | 8393 | 9105 | 9056 | 15918 | 16999 | 19054 | 1.17000735 | 1.96E-20 |
| BJK46_011470 | 4271 | 5186 | 5187 | 9171 | 8472 | 8500 | 0.927408107 | 1.68E-19 |
| BJK46_011475 | 1605 | 1870 | 2040 | 3619 | 3605 | 3375 | 0.973274914 | 1.81E-24 |
| BJK46_011480 | 5218 | 5451 | 6083 | 3789 | 4343 | 4004 | -0.40533394 | 1.09E-13 |
| BJK46_011485 | 8234 | 8638 | 9785 | 5508 | 6098 | 5609 | -0.52729484 | 2.62E-20 |
| BJK46_011490 | 6680 | 7146 | 7760 | 4588 | 4873 | 4753 | -0.52682154 | 2.64E-20 |
| BJK46_011495 | 5 | 7 | 4 | 16 | 59 | 34 | 0.167597912 | 4.43E-08 |
| BJK46_011500 | 384 | 453 | 475 | 572 | 633 | 681 | 0.053544738 | 0.0092204 |
| BJK46_011505 | 192 | 247 | 212 | 262 | 295 | 331 | -0.36852501 | 0.07745054 |
| BJK46_011510 | 303 | 382 | 296 | 427 | 401 | 470 | -0.21580948 | 0.10224544 |
| BJK46_011515 | 760 | 899 | 803 | 1002 | 1133 | 1189 | 0.233000734 | 0.012419 |
| BJK46_011520 | 5823 | 7110 | 6855 | 4446 | 4178 | 4540 | -0.51657287 | 9.51E-14 |
| BJK46_011525 | 4198 | 4498 | 4760 | 4278 | 4805 | 4390 | 0.057544232 | 0.39205591 |
| BJK46_011530 | 4903 | 5305 | 4863 | 4374 | 4208 | 4372 | -0.15966333 | 0.00131549 |
| BJK46_011535 | 352 | 419 | 377 | 330 | 356 | 360 | -0.76976697 | 0.30977091 |
| BJK46_011540 | 5346 | 5611 | 6856 | 4940 | 5044 | 4413 | -0.23483833 | 1.26E-05 |
| BJK46_011545 | 2274 | 2416 | 2875 | 2400 | 2541 | 2171 | -0.0616452 | 0.09601609 |
| BJK46_011550 | 2076 | 2210 | 2541 | 2585 | 2969 | 2680 | 0.296986983 | 0.01115839 |
| BJK46_011555 | 936 | 1136 | 1216 | 1334 | 1641 | 1376 | 0.318583226 | 0.00598905 |
| BJK46_011560 | 740 | 720 | 828 | 772 | 1315 | 842 | 0.068936926 | 0.1248564 |
| BJK46_011565 | 520 | 545 | 588 | 666 | 1134 | 728 | 0.217468253 | 0.00541643 |
| BJK46_011570 | 147 | 158 | 147 | 195 | 292 | 221 | -0.34188177 | 0.01416533 |
| BJK46_011575 | 243 | 225 | 254 | 397 | 577 | 382 | 0.193430935 | 0.0001082 |
| BJK46_011580 | 307 | 315 | 329 | 564 | 944 | 587 | 0.570606262 | 5.92E-07 |
| BJK46_011585 | 178 | 175 | 191 | 368 | 639 | 421 | 0.601197466 | 1.32E-08 |
| BJK46_011590 | 224 | 196 | 198 | 408 | 733 | 457 | 0.630677713 | 8.41E-08 |
| BJK46_011595 | 107 | 90 | 123 | 185 | 349 | 240 | 0.192411789 | 6.27E-06 |
| BJK46_011600 | 208 | 204 | 218 | 384 | 718 | 433 | 0.530069021 | 4.76E-07 |
| BJK46_011605 | 1484 | 1661 | 1626 | 696 | 805 | 821 | -1.17631783 | 1.44E-17 |
| BJK46_011620 | 18 | 17 | 21 | 29 | 38 | 30 | -1.25231154 | 0.03459189 |
| BJK46_011625 | 971 | 1128 | 1007 | 1115 | 1440 | 1199 | 0.141868241 | 0.13952538 |
| BJK46_011630 | 9356 | 10982 | 9839 | 8292 | 8071 | 8854 | -0.11002854 | 0.0006376 |
| BJK46_011635 | 2550 | 2502 | 3274 | 1991 | 2381 | 1860 | -0.39220393 | 1.59E-06 |
| BJK46_011640 | 1892 | 2030 | 2413 | 1537 | 1978 | 1399 | -0.36855127 | 0.00010416 |
| BJK46_011645 | 5931 | 6640 | 7246 | 6494 | 6687 | 6547 | 0.08605377 | 0.37521753 |
| BJK46_011650 | 4377 | 4669 | 5245 | 8051 | 7311 | 6520 | 0.687780302 | 2.17E-09 |
| BJK46_011655 | 13696 | 15119 | 16503 | 26057 | 25589 | 22904 | 0.98162283 | 1.31E-10 |
| BJK46_011660 | 21693 | 23912 | 23356 | 21293 | 21873 | 22784 | 0.212161861 | 0.2107832 |
| BJK46_011665 | 4025 | 4335 | 4598 | 2863 | 3052 | 3351 | -0.44149127 | 5.26E-11 |
| BJK46_011670 | 7857 | 8737 | 10422 | 3014 | 3251 | 2725 | -1.51711582 | 1.79E-82 |
| BJK46_011675 | 889 | 1139 | 711 | 736 | 818 | 1023 | -0.31175769 | 0.43504579 |
| BJK46_011680 | 15686 | 17654 | 18845 | 10569 | 11534 | 10960 | -0.43267428 | 1.13E-15 |
| BJK46_011685 | 5257 | 5418 | 6405 | 3117 | 3518 | 3240 | -0.73320435 | 1.30E-29 |
| BJK46_011690 | 13016 | 14050 | 15924 | 8447 | 8940 | 8286 | -0.55616911 | 1.48E-22 |
| BJK46_011695 | 1258 | 1422 | 1342 | 1648 | 1786 | 1844 | 0.365912761 | 0.00234241 |
| BJK46_011700 | 18558 | 19835 | 18768 | 15645 | 14451 | 17249 | -0.02429795 | 0.00335139 |
| BJK46_011705 | 3397 | 3647 | 3706 | 4585 | 5010 | 4874 | 0.479848786 | 1.11E-06 |
| BJK46_011710 | 756 | 815 | 925 | 1090 | 1319 | 1085 | 0.297141959 | 0.00251179 |
| BJK46_011715 | 440 | 477 | 502 | 961 | 1047 | 1009 | 0.744374641 | 6.05E-11 |
| BJK46_011720 | 473 | 525 | 519 | 782 | 980 | 864 | 0.424773853 | 8.40E-06 |
| BJK46_011725 | 1337 | 1469 | 1490 | 3556 | 3780 | 3513 | 1.357681064 | 2.17E-53 |
| BJK46_011730 | 2298 | 2847 | 2633 | 5329 | 5846 | 5411 | 1.142163749 | 1.69E-36 |
| BJK46_011735 | 2996 | 3318 | 2855 | 1598 | 1806 | 1957 | -0.75465154 | 1.42E-15 |
| BJK46_011740 | 3898 | 4668 | 5028 | 12199 | 12567 | 11557 | 1.532031776 | 2.20E-60 |
| BJK46_011745 | 20409 | 22236 | 26754 | 50660 | 52409 | 45083 | 1.417049741 | 5.46E-21 |
| BJK46_011750 | 111 | 131 | 150 | 126 | 195 | 110 | -1.03553255 | 0.80624221 |
| BJK46_011755 | 231 | 218 | 250 | 232 | 347 | 265 | -0.56001953 | 0.3671963 |
| BJK46_011760 | 196 | 200 | 219 | 235 | 288 | 304 | -0.42854012 | 0.09136889 |
| BJK46_011765 | 916 | 1035 | 871 | 1980 | 2296 | 2873 | 1.294281402 | 6.07E-19 |
| BJK46_011770 | 3532 | 3969 | 4189 | 4179 | 5797 | 4500 | 0.350821782 | 0.00776634 |
| BJK46_011775 | 10662 | 10616 | 11237 | 6670 | 6529 | 5975 | -0.63509847 | 2.63E-21 |
| BJK46_011780 | 4630 | 4990 | 5345 | 6531 | 6723 | 6143 | 0.446952121 | 2.33E-05 |
| BJK46_011785 | 2878 | 3133 | 3520 | 5270 | 5732 | 4951 | 0.795830554 | 1.60E-18 |
| BJK46_011790 | 632 | 766 | 492 | 2574 | 2391 | 3480 | 2.09081576 | 2.98E-29 |
| BJK46_011805 | 22715 | 25014 | 28498 | 141644 | 143796 | 130610 | 2.833017104 | 3.94E-101 |
| BJK46_011810 | 234 | 254 | 220 | 318 | 750 | 412 | 0.282557699 | 0.00033883 |
| BJK46_011815 | 203 | 221 | 216 | 370 | 802 | 478 | 0.608058504 | 4.35E-07 |
| BJK46_011820 | 60 | 52 | 49 | 103 | 254 | 135 | 0.154986989 | 3.19E-06 |
| BJK46_011825 | 132 | 104 | 107 | 209 | 521 | 286 | 0.502730415 | 1.52E-06 |
| BJK46_011830 | 19236 | 20983 | 23517 | 16582 | 15766 | 15441 | -0.15450785 | 3.07E-06 |
| BJK46_011835 | 10271 | 11340 | 12671 | 10091 | 9646 | 8890 | -0.08503981 | 0.0003266 |
| BJK46_011840 | 7345 | 7882 | 9086 | 6445 | 6536 | 5951 | -0.2551541 | 1.96E-07 |
| BJK46_011845 | 12448 | 14564 | 14856 | 16467 | 15531 | 16208 | 0.440717409 | 0.14427241 |
| BJK46_011890 | 66 | 84 | 69 | 122 | 276 | 175 | 0.087626586 | 1.04E-05 |
| BJK46_011895 | 5412 | 5756 | 6152 | 1549 | 1892 | 1626 | -1.74299095 | 1.39E-121 |
| BJK46_011900 | 3226 | 3475 | 3499 | 357 | 597 | 397 | -3.04841384 | 1.73E-109 |
| BJK46_011905 | 22158 | 21439 | 30168 | 1110 | 2666 | 1538 | -3.78961211 | 4.19E-66 |
| BJK46_011910 | 206 | 149 | 163 | 308 | 526 | 388 | 0.414681212 | 3.70E-06 |
| BJK46_011915 | 323 | 270 | 316 | 576 | 937 | 688 | 0.72461945 | 1.13E-08 |
| BJK46_011920 | 6717 | 7495 | 8404 | 17880 | 19505 | 16172 | 1.437303121 | 3.13E-39 |
| BJK46_011925 | 5595 | 6355 | 7311 | 14820 | 16089 | 14277 | 1.396494977 | 7.05E-44 |
| BJK46_011930 | 4011 | 4636 | 5204 | 11233 | 11432 | 10813 | 1.382639032 | 1.23E-50 |
| BJK46_011935 | 17 | 34 | 36 | 32 | 75 | 35 | -1.18475619 | 0.08226156 |
| BJK46_012000 | 12120 | 12307 | 9777 | 10067 | 10395 | 12934 | 0.149931069 | 0.43263402 |
| BJK46_012005 | 217 | 305 | 265 | 425 | 605 | 531 | 0.348585517 | 8.56E-06 |
| BJK46_012010 | 139 | 139 | 154 | 69 | 147 | 117 | -1.62269373 | 0.09866649 |
| BJK46_012015 | 3337 | 3784 | 2745 | 2639 | 2487 | 3334 | -0.18933403 | 0.04722053 |
| BJK46_012020 | 1449 | 1321 | 1103 | 819 | 960 | 1019 | -0.60752433 | 0.0010428 |
| BJK46_002810 | 12072 | 13101 | 12908 | 7929 | 6622 | 7708 | -0.62087647 | 1.35E-15 |
| BJK46_002815 | 1813 | 2076 | 2410 | 2230 | 2531 | 2265 | 0.184413634 | 0.26813734 |
| BJK46_002820 | 825 | 914 | 1127 | 924 | 1140 | 952 | -0.11024562 | 0.92721455 |
| BJK46_002825 | 2331 | 2861 | 3151 | 3067 | 3285 | 3308 | 0.252961538 | 0.0737089 |
| BJK46_002830 | 8632 | 9651 | 10463 | 10031 | 10090 | 10163 | 0.239623703 | 0.83339612 |
| BJK46_002835 | 500 | 540 | 632 | 334 | 379 | 331 | -1.2268403 | 4.66E-05 |
| BJK46_002840 | 28664 | 31927 | 36626 | 15832 | 16186 | 14694 | -0.7757267 | 4.26E-27 |
| BJK46_002845 | 57258 | 64435 | 75657 | 31638 | 32903 | 28752 | -0.72495075 | 8.64E-25 |
| BJK46_002850 | 3948 | 4167 | 5010 | 4566 | 5039 | 4124 | 0.122993346 | 0.95535776 |
| BJK46_002855 | 1742 | 1897 | 2056 | 2294 | 2479 | 2154 | 0.299908219 | 0.01164362 |
| BJK46_002860 | 3033 | 3201 | 3440 | 3074 | 3053 | 2839 | -0.07552936 | 0.03619642 |
| BJK46_002865 | 4780 | 4966 | 5610 | 5021 | 5318 | 4760 | 0.04028649 | 0.24523933 |
| BJK46_002870 | 2306 | 2581 | 2822 | 2589 | 2724 | 2460 | 0.040741092 | 0.56534642 |
| BJK46_002875 | 4323 | 4905 | 5264 | 4991 | 4965 | 4628 | 0.073037521 | 0.51951769 |
| BJK46_002880 | 10750 | 11756 | 12460 | 14433 | 13882 | 13880 | 0.483419706 | 0.0186721 |
| BJK46_002885 | 20796 | 22461 | 24789 | 27469 | 27991 | 27203 | 0.570381286 | 0.02513418 |
| BJK46_002890 | 11247 | 11706 | 14324 | 15356 | 16057 | 14115 | 0.515583353 | 0.0147579 |
| BJK46_002895 | 12044 | 12866 | 15721 | 16400 | 16989 | 15114 | 0.494641749 | 0.04805786 |
| BJK46_002900 | 13222 | 14259 | 16927 | 18171 | 18178 | 15597 | 0.471998347 | 0.10538424 |
| BJK46_002905 | 9635 | 10056 | 12138 | 12474 | 13288 | 11204 | 0.408704969 | 0.08172379 |
| BJK46_002910 | 11948 | 12714 | 14449 | 16407 | 18026 | 16155 | 0.60600624 | 0.00086954 |
| BJK46_002915 | 2812 | 2904 | 3220 | 2312 | 2341 | 2054 | -0.38873387 | 5.41E-08 |
| BJK46_002920 | 5499 | 5979 | 6501 | 5019 | 5442 | 4912 | -0.1546194 | 5.48E-05 |
| BJK46_002925 | 1566 | 1658 | 1681 | 1421 | 1556 | 1324 | -0.21894667 | 0.0166727 |
| BJK46_002930 | 700 | 780 | 766 | 396 | 537 | 428 | -1.15635382 | 6.70E-06 |
| BJK46_002935 | 1581 | 1717 | 1507 | 1346 | 1388 | 1423 | -0.23992541 | 0.02125438 |
| BJK46_002940 | 3252 | 3516 | 4242 | 5204 | 5445 | 5294 | 0.597486751 | 2.32E-09 |
| BJK46_002945 | 6634 | 7473 | 8725 | 11026 | 12004 | 10904 | 0.727598983 | 3.08E-10 |
| BJK46_002950 | 2167 | 2253 | 2545 | 1130 | 1356 | 1337 | -0.87073727 | 3.73E-22 |
| BJK46_002955 | 2523 | 2939 | 3327 | 1587 | 2018 | 1611 | -0.73228235 | 4.51E-18 |
| BJK46_002965 | 9223 | 9847 | 10335 | 11358 | 11151 | 12521 | 0.428940566 | 0.03012128 |
| BJK46_002970 | 4974 | 5560 | 6358 | 6522 | 6273 | 6536 | 0.278661445 | 0.08658255 |
| BJK46_002975 | 1090 | 1258 | 1538 | 1266 | 1512 | 1365 | 0.035230522 | 0.78000733 |
| BJK46_002980 | 13949 | 14815 | 17201 | 36029 | 33755 | 36566 | 1.494167769 | 1.82E-26 |
| BJK46_002985 | 57280 | 62151 | 63245 | 159768 | 138947 | 163802 | 1.769401883 | 3.68E-26 |
| BJK46_002990 | 1477 | 1532 | 1845 | 968 | 1077 | 971 | -0.76405239 | 3.50E-10 |
| BJK46_002995 | 2702 | 2745 | 3531 | 1628 | 1786 | 1509 | -0.8395857 | 8.42E-22 |
| BJK46_003000 | 2682 | 2929 | 3564 | 1437 | 1666 | 1382 | -1.00894327 | 6.90E-31 |
| BJK46_003005 | 19885 | 21743 | 24502 | 16300 | 17102 | 15646 | -0.16706757 | 4.13E-07 |
| BJK46_003010 | 7114 | 7995 | 8522 | 8622 | 9054 | 8219 | 0.260964363 | 0.35263878 |
| BJK46_003015 | 2180 | 2405 | 2681 | 2745 | 3089 | 2747 | 0.268703111 | 0.02695111 |
| BJK46_003020 | 2884 | 3125 | 3601 | 4021 | 4308 | 3774 | 0.378806688 | 0.00057365 |
| BJK46_003025 | 2320 | 2542 | 2919 | 4215 | 4453 | 3735 | 0.711300864 | 9.89E-13 |
| BJK46_003030 | 725 | 866 | 885 | 1803 | 1924 | 1599 | 1.003751905 | 4.07E-17 |
| BJK46_003035 | 22206 | 22387 | 22265 | 53052 | 48280 | 51764 | 1.510891028 | 1.12E-22 |
| BJK46_003040 | 2942 | 3294 | 3867 | 2320 | 2409 | 2230 | -0.50135216 | 2.47E-12 |
| BJK46_003045 | 2999 | 3298 | 3908 | 2327 | 2456 | 2060 | -0.5438264 | 1.63E-12 |
| BJK46_003050 | 7126 | 7885 | 9123 | 5829 | 6123 | 5527 | -0.36536769 | 2.84E-11 |
| BJK46_003055 | 7182 | 7892 | 9638 | 5513 | 6206 | 5427 | -0.42498573 | 1.89E-12 |
| BJK46_003060 | 4491 | 5107 | 6386 | 3338 | 3850 | 3122 | -0.57010822 | 1.18E-13 |
| BJK46_003065 | 3199 | 3402 | 4439 | 2500 | 3014 | 2352 | -0.44936824 | 3.05E-08 |
| BJK46_003070 | 67390 | 70781 | 69647 | 32813 | 32030 | 35904 | -0.68959559 | 3.50E-22 |
| BJK46_003075 | 10081 | 10903 | 12728 | 5486 | 6064 | 5689 | -0.84056181 | 1.17E-38 |
| BJK46_003080 | 6673 | 7226 | 8235 | 4018 | 4422 | 3972 | -0.76165492 | 1.46E-34 |
| BJK46_003085 | 3564 | 3869 | 4458 | 2232 | 2577 | 2465 | -0.67235382 | 3.77E-22 |
| BJK46_003090 | 145161 | 182335 | 116776 | 186735 | 149494 | 203126 | 0.768270231 | 0.22468166 |
| BJK46_003095 | 8450 | 9212 | 11122 | 7331 | 7379 | 6306 | -0.32327042 | 2.40E-08 |
| BJK46_003100 | 9584 | 10348 | 11741 | 7602 | 8519 | 7721 | -0.26056085 | 1.19E-09 |
| BJK46_003105 | 32485 | 36101 | 37935 | 29550 | 30313 | 32254 | 0.106825963 | 0.00917543 |
| BJK46_003110 | 4092 | 4608 | 5285 | 5476 | 6004 | 5771 | 0.373525535 | 0.0008686 |
| BJK46_003115 | 894 | 1053 | 1125 | 1201 | 1441 | 1212 | 0.205779583 | 0.04072892 |
| BJK46_003120 | 1619 | 1852 | 2038 | 2945 | 3033 | 2983 | 0.729649549 | 1.79E-14 |
| BJK46_003125 | 14180 | 14547 | 16100 | 14578 | 15098 | 16483 | 0.277594435 | 0.8526637 |
| BJK46_003130 | 1066 | 1143 | 1278 | 1332 | 1594 | 1276 | 0.183136136 | 0.09489228 |
| BJK46_003135 | 664 | 737 | 806 | 933 | 1147 | 927 | 0.197090687 | 0.0095323 |
| BJK46_003140 | 2584 | 3271 | 2864 | 3012 | 2725 | 2948 | 0.029555694 | 0.53216248 |
| BJK46_003145 | 2642 | 3101 | 2717 | 5122 | 5084 | 5931 | 0.97861993 | 3.50E-18 |
| BJK46_003150 | 7171 | 7750 | 8788 | 7296 | 7379 | 7360 | 0.011562193 | 0.03472326 |
| BJK46_003155 | 11116 | 12698 | 13030 | 12637 | 13226 | 13385 | 0.302402664 | 0.71588083 |
| BJK46_003160 | 4641 | 5134 | 5875 | 3465 | 3762 | 3433 | -0.49522516 | 6.68E-16 |
| BJK46_003165 | 3516 | 3593 | 4557 | 2598 | 2870 | 2361 | -0.53543336 | 3.37E-12 |
| BJK46_003170 | 2603 | 2739 | 3366 | 1864 | 2227 | 1782 | -0.5436081 | 1.14E-11 |
| BJK46_003175 | 973 | 1068 | 1124 | 1883 | 1962 | 1852 | 0.801615568 | 2.92E-13 |
| BJK46_003180 | 5674 | 6709 | 6892 | 4237 | 4490 | 3971 | -0.53416055 | 2.24E-18 |
| BJK46_003185 | 3502 | 4229 | 4343 | 3057 | 3347 | 2943 | -0.32379989 | 6.68E-08 |
| BJK46_003190 | 872 | 1089 | 1166 | 1198 | 1380 | 1304 | 0.202330504 | 0.05190764 |
| BJK46_003195 | 1756 | 2077 | 2068 | 2102 | 2356 | 2173 | 0.187109158 | 0.22266894 |
| BJK46_003200 | 175 | 172 | 231 | 285 | 486 | 317 | 0.082033563 | 0.0003542 |
| BJK46_003205 | 4015 | 3980 | 4117 | 3523 | 3920 | 3973 | -0.03954157 | 0.06275102 |
| BJK46_003210 | 4352 | 4733 | 4961 | 6980 | 6941 | 7120 | 0.657032618 | 3.71E-12 |
| BJK46_003215 | 9920 | 11495 | 11823 | 17869 | 17111 | 16063 | 0.842843543 | 5.71E-09 |
| BJK46_003220 | 5358 | 5971 | 7098 | 3452 | 3763 | 3646 | -0.69539721 | 4.45E-26 |
| BJK46_003225 | 57 | 40 | 45 | 51 | 101 | 71 | -0.97528411 | 0.07028366 |
| BJK46_003230 | 39 | 30 | 26 | 53 | 114 | 58 | -0.52776118 | 0.00080641 |
| BJK46_003235 | 26953 | 29390 | 32592 | 37068 | 37033 | 34806 | 0.609685412 | 0.02252799 |
| BJK46_003240 | 3006 | 3424 | 3903 | 4254 | 4879 | 3744 | 0.363440142 | 0.00415793 |
| BJK46_003245 | 7722 | 8483 | 8802 | 9551 | 9084 | 9266 | 0.298401648 | 0.23426956 |
| BJK46_003250 | 6610 | 6897 | 7915 | 9743 | 9824 | 9507 | 0.572505095 | 1.63E-06 |
| BJK46_003255 | 8739 | 8542 | 10801 | 6136 | 6953 | 5596 | -0.47327417 | 5.95E-13 |
| BJK46_003260 | 5983 | 6157 | 7549 | 4471 | 4832 | 3924 | -0.50446609 | 2.11E-13 |
| BJK46_003265 | 6009 | 6223 | 7565 | 4299 | 4786 | 3947 | -0.53368718 | 5.90E-16 |
| BJK46_003270 | 6533 | 6923 | 8223 | 4745 | 5394 | 4621 | -0.47512776 | 1.93E-15 |
| BJK46_003275 | 3989 | 4281 | 5119 | 2810 | 3230 | 2743 | -0.56156333 | 2.42E-16 |
| BJK46_003280 | 3530 | 3724 | 4405 | 2987 | 3282 | 2929 | -0.29774782 | 3.92E-07 |
| BJK46_003285 | 5737 | 6056 | 6910 | 5288 | 6036 | 5471 | -0.07822986 | 0.00260761 |
| BJK46_003290 | 1242 | 1295 | 1602 | 1195 | 1459 | 1160 | -0.18711882 | 0.14923552 |
| BJK46_003295 | 21718 | 22428 | 25755 | 17612 | 17602 | 16114 | -0.17820222 | 6.11E-07 |
| BJK46_003300 | 6632 | 7030 | 7960 | 5188 | 5283 | 4530 | -0.44983955 | 4.92E-13 |
| BJK46_003305 | 3364 | 3792 | 3972 | 2598 | 2719 | 2534 | -0.46706175 | 3.61E-13 |
| BJK46_003310 | 8921 | 9152 | 10789 | 6903 | 7354 | 6307 | -0.36343593 | 3.41E-11 |
| BJK46_003315 | 3644 | 3920 | 4305 | 3050 | 3083 | 2838 | -0.3620072 | 4.74E-09 |
| BJK46_003320 | 3269 | 3708 | 4203 | 2838 | 3070 | 2688 | -0.33769814 | 7.51E-08 |
| BJK46_003325 | 8173 | 8341 | 10082 | 7590 | 8198 | 6499 | -0.13225897 | 0.00055924 |
| BJK46_003330 | 3181 | 3162 | 3868 | 3123 | 3581 | 2891 | -0.05243881 | 0.08245156 |
| BJK46_003335 | 26218 | 30992 | 29601 | 22752 | 23162 | 25783 | 0.016275118 | 0.00194826 |
| BJK46_003340 | 6312 | 6962 | 7900 | 7891 | 8264 | 7306 | 0.259765619 | 0.26141476 |
| BJK46_003345 | 5779 | 6807 | 4745 | 12206 | 13399 | 16345 | 1.42090323 | 9.28E-17 |
| BJK46_003350 | 2836 | 3310 | 2378 | 5234 | 5279 | 6529 | 1.053621318 | 6.18E-12 |
| BJK46_003355 | 3221 | 3677 | 3891 | 2872 | 2837 | 2915 | -0.28362309 | 2.66E-06 |
| BJK46_003360 | 11934 | 13601 | 9091 | 14379 | 16248 | 22273 | 0.830920115 | 0.00242472 |
| BJK46_003365 | 41498 | 44436 | 53971 | 23193 | 24255 | 20232 | -0.73059014 | 4.40E-23 |
| BJK46_003370 | 33273 | 36106 | 45793 | 18070 | 18785 | 14445 | -0.87487111 | 5.56E-23 |
| BJK46_003375 | 25384 | 28660 | 34667 | 14641 | 15673 | 12905 | -0.76270695 | 3.20E-23 |
| BJK46_003380 | 7085 | 8308 | 7667 | 7330 | 6434 | 7233 | -0.02904267 | 0.04931369 |
| BJK46_003385 | 145 | 172 | 176 | 141 | 194 | 182 | -0.96501771 | 1 |
| BJK46_003390 | 44 | 48 | 45 | 67 | 120 | 57 | -0.81778735 | 0.01564229 |
| BJK46_003395 | 32 | 34 | 29 | 59 | 119 | 52 | -0.51850444 | 0.00048461 |
| BJK46_003400 | 76 | 50 | 67 | 111 | 277 | 147 | 0.09611458 | 3.08E-05 |
| BJK46_003405 | 50 | 46 | 45 | 73 | 155 | 105 | -0.31139082 | 0.00013802 |
| BJK46_003410 | 74 | 83 | 67 | 142 | 270 | 154 | 0.060175284 | 1.11E-05 |
| BJK46_003415 | 146 | 177 | 161 | 184 | 350 | 202 | -0.40101455 | 0.04146796 |
| BJK46_003420 | 4517 | 4905 | 5836 | 5146 | 5330 | 4818 | 0.073273991 | 0.49308648 |
| BJK46_003425 | 3460 | 3658 | 4393 | 3924 | 4090 | 3561 | 0.059602292 | 0.54537019 |
| BJK46_003430 | 6876 | 7280 | 8816 | 8284 | 8166 | 7026 | 0.150161421 | 0.7804766 |
| BJK46_003435 | 2268 | 2548 | 2928 | 2825 | 2883 | 2497 | 0.114579399 | 0.7725881 |
| BJK46_003440 | 7282 | 7951 | 9257 | 8643 | 9083 | 7970 | 0.202751869 | 0.8942827 |
| BJK46_003445 | 3887 | 4286 | 4960 | 4654 | 5297 | 4594 | 0.208570187 | 0.25435775 |
| BJK46_003450 | 6176 | 7832 | 3916 | 2569 | 1824 | 2502 | -1.2952615 | 4.06E-11 |
| BJK46_003455 | 357 | 409 | 399 | 332 | 438 | 356 | -0.67563936 | 0.54449123 |
| BJK46_003460 | 335 | 327 | 404 | 299 | 492 | 365 | -0.54466959 | 0.83496078 |
| BJK46_003465 | 237 | 180 | 221 | 246 | 415 | 282 | -0.27647415 | 0.04667602 |
| BJK46_003470 | 248 | 238 | 265 | 285 | 462 | 340 | -0.22971109 | 0.03622212 |
| BJK46_003475 | 559 | 555 | 560 | 489 | 548 | 499 | -0.58738414 | 0.27594293 |
| BJK46_003480 | 14029 | 15023 | 14846 | 23430 | 22008 | 23975 | 0.917545739 | 1.17E-08 |
| BJK46_003485 | 48261 | 51592 | 44166 | 101752 | 105990 | 129567 | 1.625464283 | 9.68E-18 |
| BJK46_003490 | 11820 | 13322 | 13962 | 11445 | 11255 | 11191 | -0.0021858 | 0.00204138 |
| BJK46_003495 | 2139 | 2513 | 2252 | 1916 | 2013 | 2264 | -0.1382098 | 0.03207048 |
| BJK46_003500 | 3142 | 3633 | 3705 | 1714 | 1988 | 2046 | -0.84153552 | 9.52E-27 |
| BJK46_003505 | 1949 | 2477 | 2694 | 641 | 812 | 657 | -1.82881625 | 1.23E-52 |
| BJK46_003510 | 8378 | 9221 | 10240 | 4321 | 4498 | 4542 | -0.96967756 | 2.75E-48 |
| BJK46_003515 | 8425 | 9240 | 11010 | 4264 | 4753 | 4507 | -0.9895786 | 3.46E-46 |
| BJK46_003520 | 7159 | 7840 | 9063 | 11128 | 11054 | 10558 | 0.600749113 | 3.09E-06 |
| BJK46_003525 | 1296 | 1521 | 1576 | 1558 | 1702 | 1547 | 0.106598596 | 0.50749347 |
| BJK46_003530 | 822 | 801 | 1081 | 886 | 1064 | 843 | -0.1657725 | 0.9283449 |
| BJK46_003535 | 7252 | 7822 | 9725 | 10437 | 10897 | 9463 | 0.467935623 | 0.00443634 |
| BJK46_003540 | 7715 | 8509 | 9951 | 6626 | 7125 | 6293 | -0.26668565 | 2.78E-08 |
| BJK46_003545 | 5694 | 6085 | 7203 | 3591 | 4065 | 3599 | -0.68902722 | 1.90E-27 |
| BJK46_003550 | 4885 | 5461 | 6290 | 3507 | 3921 | 3613 | -0.52935047 | 6.97E-18 |
| BJK46_003555 | 17019 | 18988 | 21520 | 9841 | 10251 | 9147 | -0.75095319 | 2.29E-27 |
| BJK46_003560 | 6216 | 6221 | 7039 | 5605 | 5663 | 5305 | -0.15436156 | 0.0001282 |
| BJK46_003565 | 9565 | 9696 | 11323 | 9105 | 9940 | 8942 | 0.032951205 | 0.01797115 |
| BJK46_003570 | 6306 | 6551 | 7663 | 5902 | 6429 | 5637 | -0.10432684 | 0.00081495 |
| BJK46_003575 | 3763 | 3987 | 4859 | 3447 | 3857 | 3257 | -0.20415246 | 0.00014282 |
| BJK46_003580 | 4919 | 5231 | 6485 | 4644 | 4838 | 4148 | -0.21820028 | 5.12E-05 |
| BJK46_003585 | 9910 | 10436 | 11944 | 8916 | 10009 | 9498 | -0.01599001 | 0.00178829 |
| BJK46_003590 | 22048 | 22988 | 24445 | 27709 | 27742 | 31973 | 0.619145561 | 0.01296806 |
| BJK46_003595 | 29515 | 30915 | 34502 | 39058 | 35818 | 38643 | 0.579470825 | 0.06511589 |
| BJK46_003600 | 12389 | 13038 | 14920 | 13893 | 13083 | 14272 | 0.255001715 | 0.79676678 |
| BJK46_003605 | 664 | 577 | 629 | 405 | 585 | 570 | -0.71387967 | 0.08461041 |
| BJK46_003610 | 25030 | 34016 | 24931 | 36666 | 24132 | 28769 | 0.386695366 | 0.82016552 |
| BJK46_003615 | 10054 | 11275 | 13288 | 5797 | 6069 | 5386 | -0.87329959 | 2.10E-35 |
| BJK46_003620 | 3707 | 3936 | 4546 | 2882 | 3048 | 2645 | -0.46566226 | 3.32E-12 |
| BJK46_003625 | 5180 | 5494 | 6312 | 6552 | 7064 | 6618 | 0.336761044 | 0.00864599 |
| BJK46_003630 | 5916 | 6478 | 7568 | 8300 | 9075 | 7591 | 0.434006546 | 0.00141682 |
| BJK46_003635 | 26471 | 28846 | 32925 | 52325 | 55486 | 48884 | 1.165272509 | 2.13E-13 |
| BJK46_003640 | 7604 | 8641 | 9671 | 16646 | 18429 | 16376 | 1.195266516 | 4.97E-27 |
| BJK46_003645 | 8762 | 10939 | 11523 | 25517 | 24393 | 23356 | 1.48065425 | 1.29E-28 |
| BJK46_003650 | 1177 | 1298 | 1581 | 789 | 970 | 864 | -0.7684799 | 3.87E-07 |
| BJK46_003655 | 14021 | 16458 | 17915 | 13286 | 13357 | 13249 | -0.04115532 | 0.00053634 |
| BJK46_003660 | 18710 | 25517 | 13465 | 13163 | 10059 | 15832 | -0.30122837 | 0.00388369 |
| BJK46_003665 | 2476 | 2952 | 2487 | 2441 | 2587 | 3086 | 0.059785897 | 0.80152442 |
| BJK46_003670 | 624 | 751 | 785 | 1012 | 1376 | 1050 | 0.44093336 | 6.27E-05 |
| BJK46_003675 | 1289 | 1580 | 1507 | 2325 | 2357 | 2228 | 0.668576751 | 5.11E-10 |
| BJK46_003680 | 49 | 49 | 40 | 62 | 194 | 123 | -0.15750159 | 0.00010503 |
| BJK46_003685 | 12048 | 12989 | 14632 | 9220 | 9060 | 9180 | -0.34394063 | 4.78E-12 |
| BJK46_003690 | 7923 | 9042 | 10422 | 6427 | 6263 | 5821 | -0.44814212 | 4.89E-13 |
| BJK46_003695 | 7535 | 8424 | 9703 | 5679 | 6051 | 5575 | -0.4634064 | 1.40E-15 |
| BJK46_003700 | 5438 | 5975 | 7028 | 3877 | 3909 | 3480 | -0.64526371 | 4.77E-21 |
| BJK46_003705 | 2870 | 3219 | 3728 | 2130 | 2132 | 1899 | -0.64051287 | 5.91E-16 |
| BJK46_003710 | 5504 | 6013 | 6989 | 3717 | 4067 | 3479 | -0.65269069 | 7.77E-24 |
| BJK46_003715 | 6401 | 6909 | 8175 | 4241 | 4603 | 4333 | -0.62832222 | 2.44E-24 |
| BJK46_003720 | 2993 | 3361 | 3659 | 2340 | 2685 | 2572 | -0.3659047 | 7.33E-09 |
| BJK46_003725 | 2019 | 2374 | 2467 | 1936 | 2177 | 1896 | -0.17035272 | 0.00367953 |
| BJK46_003730 | 10519 | 11100 | 12555 | 8138 | 7620 | 7939 | -0.37182966 | 1.78E-11 |
| BJK46_003735 | 5746 | 6113 | 7023 | 4020 | 3977 | 3658 | -0.6309577 | 6.55E-22 |
| BJK46_003740 | 8407 | 8936 | 10654 | 5485 | 5693 | 4945 | -0.69180848 | 1.10E-23 |
| BJK46_003745 | 2791 | 2854 | 3648 | 1811 | 1966 | 1665 | -0.74224272 | 1.54E-18 |
| BJK46_003750 | 4651 | 4999 | 6068 | 3088 | 3393 | 2920 | -0.68542649 | 4.45E-22 |
| BJK46_003755 | 2084 | 2458 | 2715 | 1399 | 1550 | 1312 | -0.75692528 | 2.92E-18 |
| BJK46_003760 | 10336 | 11244 | 13360 | 11152 | 11946 | 11994 | 0.206834285 | 0.55890722 |
| BJK46_003765 | 7831 | 8532 | 10133 | 8185 | 9316 | 9074 | 0.14935565 | 0.50467973 |
| BJK46_003770 | 3791 | 4270 | 5120 | 3687 | 4153 | 3418 | -0.17144579 | 0.00094939 |
| BJK46_003775 | 1728 | 1993 | 2302 | 1796 | 2018 | 1639 | -0.12915275 | 0.04083469 |
| BJK46_003780 | 1154 | 1314 | 1424 | 1001 | 1178 | 1012 | -0.39827993 | 0.00522467 |
| BJK46_003785 | 7535 | 8418 | 8317 | 4775 | 4971 | 5196 | -0.61542622 | 7.92E-23 |
| BJK46_003790 | 9234 | 10244 | 11412 | 6532 | 6998 | 6998 | -0.45567645 | 1.33E-16 |
| BJK46_003795 | 4256 | 4982 | 5387 | 3337 | 3399 | 3303 | -0.48655802 | 1.88E-14 |
| BJK46_003800 | 10774 | 11374 | 13314 | 7758 | 8755 | 7327 | -0.41425486 | 3.62E-14 |
| BJK46_003805 | 8094 | 9022 | 10702 | 6039 | 6220 | 5206 | -0.56084307 | 4.70E-16 |
| BJK46_003810 | 3853 | 4216 | 5004 | 3279 | 3661 | 3211 | -0.31253118 | 9.56E-08 |
| BJK46_003815 | 3834 | 4280 | 5051 | 3445 | 3769 | 3163 | -0.29052717 | 1.65E-06 |
| BJK46_003820 | 2101 | 2381 | 2625 | 2388 | 2912 | 2304 | 0.118885747 | 0.70091804 |
| BJK46_003825 | 2545 | 2669 | 3170 | 3535 | 4030 | 3614 | 0.454364256 | 8.10E-06 |
| BJK46_003830 | 38166 | 49750 | 29841 | 29540 | 24762 | 43895 | 0.047514098 | 0.13410676 |
| BJK46_003835 | 7406 | 8436 | 8872 | 8445 | 9067 | 9054 | 0.240084632 | 0.56689622 |
| BJK46_003840 | 2954 | 3318 | 3489 | 3488 | 4058 | 4055 | 0.290247136 | 0.0185849 |
| BJK46_003845 | 2337 | 2525 | 2601 | 3075 | 3329 | 3178 | 0.389700181 | 0.00013985 |
| BJK46_003850 | 5253 | 5884 | 5625 | 5370 | 4375 | 5017 | -0.12197536 | 0.01875455 |
| BJK46_003855 | 2277 | 2696 | 3075 | 1824 | 2017 | 1771 | -0.49161599 | 2.30E-10 |
| BJK46_003865 | 9227 | 9816 | 11603 | 3517 | 4069 | 3574 | -1.37380784 | 6.31E-81 |
| BJK46_003870 | 4979 | 5315 | 6606 | 1995 | 2482 | 2179 | -1.29831732 | 2.06E-58 |
| BJK46_003875 | 8137 | 9372 | 10133 | 5634 | 5760 | 5336 | -0.61758892 | 2.74E-23 |
| BJK46_003880 | 4000 | 4736 | 5462 | 2584 | 3026 | 2706 | -0.72079106 | 1.74E-23 |
| BJK46_003885 | 8827 | 10321 | 11581 | 6353 | 7326 | 6464 | -0.47666021 | 1.12E-16 |
| BJK46_003890 | 6242 | 6513 | 7090 | 3220 | 3303 | 3411 | -0.93851808 | 1.27E-45 |
| BJK46_003895 | 6895 | 7395 | 8364 | 5541 | 6342 | 6056 | -0.24342673 | 4.97E-08 |
| BJK46_003900 | 37980 | 40146 | 38228 | 39782 | 30967 | 35974 | 0.195232658 | 0.16677708 |
| BJK46_003905 | 48623 | 50502 | 41962 | 46276 | 44360 | 52093 | 0.371851243 | 0.70297227 |
| BJK46_003910 | 38763 | 41222 | 36166 | 35821 | 37718 | 44267 | 0.348876346 | 0.71069827 |
| BJK46_003915 | 4072 | 4543 | 5560 | 2908 | 3196 | 2508 | -0.67040325 | 2.05E-16 |
| BJK46_003920 | 4156 | 4458 | 5443 | 2716 | 3040 | 2460 | -0.72878556 | 2.77E-21 |
| BJK46_003925 | 2656 | 2745 | 3596 | 1645 | 1785 | 1353 | -0.88847689 | 2.44E-18 |
| BJK46_003930 | 2216 | 2218 | 2974 | 1346 | 1502 | 1252 | -0.8385972 | 1.14E-17 |
| BJK46_003935 | 4965 | 4961 | 6482 | 3261 | 3548 | 2699 | -0.73367119 | 9.64E-17 |
| BJK46_003940 | 23467 | 24701 | 29300 | 12921 | 10532 | 10258 | -0.95322411 | 6.58E-24 |
| BJK46_003945 | 38295 | 41419 | 48168 | 17621 | 15630 | 15151 | -1.10763378 | 6.10E-36 |
| BJK46_003950 | 49061 | 55134 | 59060 | 23029 | 20819 | 21849 | -0.99109461 | 3.74E-34 |
| BJK46_003955 | 4808 | 5314 | 5930 | 5498 | 5399 | 5069 | 0.062998798 | 0.40111876 |
| BJK46_003960 | 5967 | 6797 | 7422 | 8546 | 8884 | 8095 | 0.454020084 | 0.00029821 |
| BJK46_003965 | 1551 | 1737 | 1716 | 23330 | 21360 | 24420 | 3.867553719 | 4.80E-255 |
| BJK46_003970 | 103 | 107 | 105 | 155 | 230 | 161 | -0.36065158 | 0.00374678 |
| BJK46_003975 | 195 | 209 | 217 | 310 | 493 | 328 | 0.060995686 | 0.00038 |
| BJK46_003980 | 252 | 273 | 302 | 497 | 696 | 537 | 0.452337299 | 2.66E-07 |
| BJK46_003985 | 8948 | 10543 | 12366 | 8668 | 8284 | 7801 | -0.20312086 | 8.14E-06 |
| BJK46_003990 | 15260 | 17104 | 20935 | 10507 | 10779 | 9634 | -0.55640413 | 7.69E-17 |
| BJK46_003995 | 1517 | 1465 | 1645 | 1211 | 1342 | 1319 | -0.30587527 | 0.00414888 |
| BJK46_004000 | 612 | 664 | 734 | 1053 | 1137 | 926 | 0.372078495 | 0.0001447 |
| BJK46_004005 | 15446 | 17616 | 20071 | 14046 | 14550 | 13344 | -0.09418857 | 4.76E-05 |
| BJK46_004010 | 8159 | 9864 | 11436 | 7274 | 8042 | 6739 | -0.27702356 | 1.64E-07 |
| BJK46_004015 | 5604 | 6204 | 7431 | 4646 | 5334 | 4490 | -0.33570484 | 5.50E-09 |
| BJK46_004020 | 8074 | 9032 | 10806 | 6217 | 6846 | 5844 | -0.44202658 | 2.10E-13 |
| BJK46_004025 | 4905 | 5629 | 6255 | 4168 | 4046 | 3585 | -0.44666523 | 2.50E-11 |
| BJK46_004030 | 20013 | 21865 | 23770 | 17893 | 18245 | 18150 | -0.00627436 | 0.00059935 |
| BJK46_004035 | 1354 | 1544 | 1633 | 2142 | 2313 | 2157 | 0.552743021 | 6.46E-08 |
| BJK46_004040 | 183 | 198 | 220 | 223 | 297 | 230 | -0.56878931 | 0.24725972 |
| BJK46_012025 | 37 | 42 | 42 | 124 | 273 | 150 | 0.691484549 | 2.74E-11 |
| BJK46_012030 | 63 | 71 | 59 | 162 | 442 | 233 | 0.822775251 | 7.88E-10 |
| BJK46_012035 | 40 | 41 | 32 | 90 | 237 | 131 | 0.437034913 | 1.98E-08 |
| BJK46_012045 | 21864 | 20205 | 24748 | 602 | 1043 | 695 | -4.81943291 | 2.28E-219 |
| BJK46_012080 | 0 | 0 | 0 | 1 | 0 | 1 | -2.79558426 | 0.50425199 |
| BJK46_012090 | 103 | 97 | 117 | 179 | 252 | 210 | -0.08099178 | 8.13E-05 |
| BJK46_004045 | 61324 | 65320 | 75012 | 42522 | 45311 | 42242 | -0.25905981 | 2.93E-11 |
| BJK46_004050 | 22675 | 24871 | 29136 | 15676 | 17248 | 15716 | -0.38406213 | 5.59E-13 |
| BJK46_004055 | 2152 | 2273 | 2882 | 1564 | 1950 | 1401 | -0.56124876 | 1.19E-08 |
| BJK46_004060 | 1375 | 1462 | 1740 | 1079 | 1299 | 1035 | -0.49520852 | 7.13E-05 |
| BJK46_004065 | 42893 | 41010 | 37905 | 67358 | 75312 | 83526 | 1.261047124 | 1.08E-10 |
| BJK46_004070 | 3112 | 3053 | 3710 | 3583 | 4149 | 3352 | 0.20657885 | 0.23495222 |
| BJK46_004075 | 13963 | 15618 | 14267 | 5588 | 5957 | 6043 | -1.16869421 | 1.86E-54 |
| BJK46_004080 | 10417 | 11027 | 9859 | 2805 | 2936 | 3294 | -1.72413107 | 2.83E-78 |
| BJK46_004085 | 10655 | 11604 | 13726 | 7353 | 8029 | 6863 | -0.5374751 | 8.88E-19 |
| BJK46_004090 | 9349 | 10269 | 11381 | 9859 | 10251 | 8534 | 0.047899538 | 0.04625465 |
| BJK46_004095 | 4065 | 4240 | 4590 | 4150 | 4137 | 3518 | -0.07924305 | 0.02776388 |
| BJK46_004100 | 5944 | 6605 | 7833 | 7151 | 7693 | 6545 | 0.172206445 | 0.88876858 |
| BJK46_004105 | 3035 | 3420 | 3853 | 3853 | 4109 | 3523 | 0.202551199 | 0.23377417 |
| BJK46_004110 | 7446 | 7981 | 9529 | 8961 | 9511 | 8055 | 0.22546526 | 0.73223407 |
| BJK46_004115 | 7675 | 7989 | 9445 | 7907 | 8722 | 7365 | 0.061385319 | 0.12271795 |
| BJK46_004120 | 9642 | 10122 | 11740 | 12930 | 12753 | 12749 | 0.483962399 | 0.00881374 |
| BJK46_004125 | 8127 | 8729 | 10207 | 9464 | 9449 | 8964 | 0.194736781 | 0.8623117 |
| BJK46_004130 | 14468 | 16145 | 18391 | 17705 | 18892 | 17207 | 0.389776661 | 0.42502562 |
| BJK46_004135 | 1031 | 1141 | 1241 | 1674 | 2083 | 2048 | 0.728624732 | 2.98E-10 |
| BJK46_004140 | 5399 | 5796 | 6288 | 10276 | 10282 | 9742 | 0.909110828 | 7.34E-21 |
| BJK46_004145 | 6793 | 7363 | 7873 | 15304 | 16354 | 14394 | 1.239619664 | 5.75E-34 |
| BJK46_004150 | 8227 | 9102 | 9546 | 10025 | 9557 | 9626 | 0.272464402 | 0.45991296 |
| BJK46_004155 | 22999 | 23412 | 24378 | 38785 | 38344 | 39703 | 1.028644688 | 3.17E-10 |
| BJK46_004160 | 4515 | 4485 | 4458 | 6103 | 6316 | 6507 | 0.557616498 | 2.28E-07 |
| BJK46_004165 | 2835 | 3633 | 2196 | 1304 | 1273 | 1830 | -0.95097418 | 1.61E-08 |
| BJK46_004170 | 60656 | 68821 | 73664 | 82628 | 79166 | 87178 | 0.702304891 | 0.03082184 |
| BJK46_004175 | 16175 | 16725 | 18431 | 10920 | 10177 | 10433 | -0.48399234 | 1.51E-15 |
| BJK46_004180 | 1081 | 1199 | 1313 | 1733 | 1882 | 1649 | 0.512090392 | 5.69E-06 |
| BJK46_004185 | 7975 | 8214 | 9660 | 7838 | 7281 | 7596 | -0.05949247 | 0.00563728 |
| BJK46_004190 | 3938 | 4569 | 5151 | 3339 | 3480 | 3412 | -0.36037805 | 2.33E-09 |
| BJK46_004195 | 5102 | 5788 | 6595 | 3669 | 4047 | 3577 | -0.56680675 | 6.56E-20 |
| BJK46_004200 | 3729 | 4014 | 5015 | 1635 | 1728 | 1419 | -1.38329505 | 1.86E-51 |
| BJK46_004205 | 6654 | 7257 | 8624 | 2650 | 2948 | 2373 | -1.44117351 | 2.52E-75 |
| BJK46_004210 | 6227 | 6927 | 7984 | 2487 | 2755 | 2318 | -1.42749748 | 1.95E-83 |
| BJK46_004215 | 3694 | 3794 | 4531 | 1778 | 1992 | 1699 | -1.10619065 | 9.67E-45 |
| BJK46_004220 | 11767 | 11859 | 14897 | 6357 | 6987 | 6110 | -0.83414828 | 7.07E-33 |
| BJK46_004225 | 16019 | 17758 | 20064 | 13469 | 13333 | 12074 | -0.22971516 | 1.32E-07 |
| BJK46_004230 | 7 | 4 | 10 | 23 | 51 | 14 | -0.46052296 | 2.49E-05 |
| BJK46_004235 | 27464 | 29844 | 36187 | 19517 | 20449 | 18264 | -0.3906839 | 2.15E-12 |
| BJK46_004240 | 37953 | 40163 | 42792 | 27002 | 24198 | 24315 | -0.36859062 | 2.72E-11 |
| BJK46_004245 | 8350 | 8989 | 10461 | 9492 | 10081 | 9090 | 0.20030848 | 0.85356352 |
| BJK46_004250 | 3691 | 4078 | 4651 | 3654 | 4084 | 3656 | -0.0715408 | 0.01554318 |
| BJK46_004255 | 7825 | 9284 | 6595 | 8322 | 7197 | 8963 | 0.175201092 | 0.89416977 |
| BJK46_004260 | 398 | 458 | 461 | 470 | 565 | 540 | -0.25547094 | 0.28022811 |
| BJK46_004265 | 24664 | 25378 | 30005 | 12954 | 14273 | 12187 | -0.7614255 | 1.08E-26 |
| BJK46_004270 | 4220 | 4192 | 5421 | 1963 | 2183 | 1745 | -1.19534646 | 7.96E-41 |
| BJK46_004275 | 4654 | 4547 | 5822 | 2040 | 2387 | 1878 | -1.21505418 | 3.01E-44 |
| BJK46_004280 | 12125 | 12400 | 15442 | 5559 | 6154 | 5011 | -1.11946002 | 1.19E-46 |
| BJK46_004285 | 4774 | 4970 | 5974 | 2244 | 2439 | 2185 | -1.14913501 | 7.75E-55 |
| BJK46_004290 | 10347 | 10958 | 13123 | 12764 | 13154 | 11197 | 0.309293625 | 0.58968467 |
| BJK46_004295 | 16784 | 18110 | 21942 | 31751 | 30319 | 27406 | 0.942198118 | 6.02E-08 |
| BJK46_004300 | 8585 | 9751 | 10613 | 20195 | 19566 | 19314 | 1.255986425 | 1.54E-23 |
| BJK46_004305 | 5299 | 5809 | 6716 | 12381 | 12099 | 10613 | 1.108687535 | 2.02E-24 |
| BJK46_004310 | 3206 | 3488 | 4036 | 7244 | 7480 | 6636 | 1.060215353 | 9.06E-33 |
| BJK46_004315 | 3038 | 3413 | 3666 | 7735 | 7981 | 7348 | 1.255085601 | 5.20E-52 |
| BJK46_004320 | 8821 | 9117 | 10889 | 7658 | 7839 | 6935 | -0.22672312 | 6.75E-07 |
| BJK46_004325 | 9796 | 10315 | 12907 | 8028 | 8519 | 7144 | -0.32329179 | 4.76E-09 |
| BJK46_004330 | 41025 | 46837 | 51338 | 28225 | 29075 | 28105 | -0.37415979 | 1.48E-13 |
| BJK46_004335 | 59779 | 63048 | 73913 | 43638 | 45168 | 43064 | -0.20309568 | 1.79E-09 |
| BJK46_004340 | 10684 | 12576 | 13623 | 10648 | 11127 | 9938 | -0.02356702 | 0.00123807 |
| BJK46_004345 | 11369 | 12513 | 14780 | 9901 | 10827 | 9576 | -0.15615146 | 1.94E-06 |
| BJK46_004350 | 2542 | 2586 | 3234 | 1783 | 2068 | 1699 | -0.5671174 | 2.95E-12 |
| BJK46_004355 | 3673 | 3927 | 4572 | 2076 | 2478 | 2095 | -0.83962736 | 4.74E-30 |
| BJK46_004360 | 6731 | 7212 | 8962 | 4742 | 4918 | 4408 | -0.61762821 | 2.43E-19 |
| BJK46_004365 | 8406 | 9117 | 11101 | 6279 | 6837 | 5569 | -0.49680624 | 4.99E-14 |
| BJK46_004370 | 1061 | 1044 | 1221 | 524 | 798 | 537 | -1.12829046 | 7.07E-08 |
| BJK46_004375 | 54915 | 60263 | 69809 | 44792 | 44515 | 38774 | -0.16329635 | 1.44E-07 |
| BJK46_004380 | 63183 | 68771 | 82446 | 49741 | 50523 | 43284 | -0.19538391 | 1.30E-08 |
| BJK46_004385 | 14719 | 17156 | 12013 | 20638 | 22027 | 30347 | 0.986445923 | 7.71E-05 |
| BJK46_004390 | 4153 | 4600 | 5098 | 4122 | 4415 | 3999 | -0.08576254 | 0.00631553 |
| BJK46_004395 | 4375 | 4905 | 5419 | 4721 | 4837 | 4668 | 0.017860081 | 0.15172605 |
| BJK46_004400 | 3641 | 4020 | 4189 | 4505 | 4492 | 4841 | 0.27805824 | 0.04129528 |
| BJK46_004405 | 4234 | 4487 | 4946 | 6154 | 6490 | 6396 | 0.547909942 | 6.30E-09 |
| BJK46_004410 | 3263 | 3339 | 3536 | 7792 | 7097 | 7929 | 1.232108698 | 4.38E-36 |
| BJK46_004415 | 1777 | 2001 | 1890 | 3985 | 4103 | 4354 | 1.162970184 | 4.50E-34 |
| BJK46_004420 | 4785 | 5607 | 5566 | 7019 | 7126 | 7634 | 0.532275307 | 1.21E-06 |
| BJK46_004425 | 2878 | 3275 | 3540 | 4797 | 4926 | 5020 | 0.658618034 | 1.76E-12 |
| BJK46_004430 | 3481 | 4161 | 3804 | 7013 | 6970 | 7702 | 0.989154513 | 1.42E-22 |
| BJK46_004435 | 168 | 204 | 162 | 184 | 237 | 202 | -0.73517065 | 0.51847412 |
| BJK46_004440 | 68062 | 66801 | 66783 | 39783 | 44214 | 50517 | -0.22001421 | 6.38E-08 |
| BJK46_004445 | 3575 | 3953 | 4655 | 2149 | 2421 | 2134 | -0.82393563 | 4.42E-29 |
| BJK46_004450 | 2511 | 2869 | 3318 | 1395 | 1569 | 1421 | -0.96527849 | 2.39E-31 |
| BJK46_004455 | 30527 | 33759 | 30249 | 12136 | 13598 | 15287 | -0.94087743 | 3.61E-27 |
| BJK46_004460 | 1995 | 2209 | 2342 | 1471 | 1552 | 1423 | -0.55355132 | 7.19E-12 |
| BJK46_004465 | 756 | 849 | 905 | 594 | 621 | 595 | -0.79860351 | 0.00070731 |
| BJK46_004470 | 511 | 534 | 505 | 403 | 419 | 465 | -0.79068002 | 0.07433617 |
| BJK46_004475 | 451 | 577 | 695 | 285 | 371 | 303 | -1.3998307 | 4.61E-06 |
| BJK46_004480 | 172 | 222 | 281 | 63 | 101 | 61 | -2.75767616 | 1.03E-09 |
| BJK46_004485 | 339 | 339 | 415 | 125 | 150 | 137 | -2.28025785 | 7.04E-12 |
| BJK46_004490 | 344 | 331 | 417 | 132 | 162 | 149 | -2.1571704 | 1.81E-10 |
| BJK46_004495 | 269 | 308 | 326 | 86 | 168 | 110 | -2.32582329 | 3.26E-08 |
| BJK46_004500 | 238 | 307 | 240 | 255 | 340 | 364 | -0.47167653 | 0.32444708 |
| BJK46_004505 | 204 | 268 | 208 | 308 | 369 | 423 | -0.06270049 | 0.00490974 |
| BJK46_004510 | 41 | 28 | 33 | 55 | 169 | 92 | -0.10804453 | 2.82E-05 |
| BJK46_004515 | 15188 | 16802 | 18906 | 16068 | 16577 | 16541 | 0.202705761 | 0.26309103 |
| BJK46_004520 | 3942 | 4247 | 4944 | 3607 | 4083 | 3833 | -0.13371495 | 0.00108149 |
| BJK46_004525 | 6481 | 6999 | 8097 | 4939 | 4967 | 5115 | -0.43974839 | 1.08E-13 |
| BJK46_004530 | 10585 | 11367 | 13474 | 6536 | 7405 | 6578 | -0.64067886 | 1.45E-25 |
| BJK46_004535 | 1710 | 1841 | 2105 | 1130 | 1377 | 1239 | -0.62051123 | 2.09E-10 |
| BJK46_004540 | 109320 | 117105 | 86877 | 58315 | 53576 | 65782 | -0.4020168 | 6.39E-09 |
| BJK46_004545 | 19141 | 20193 | 24402 | 17117 | 17771 | 15928 | -0.06081599 | 0.00014323 |
| BJK46_004550 | 19317 | 20328 | 24884 | 16503 | 17088 | 14263 | -0.16977518 | 4.80E-06 |
| BJK46_004555 | 20610 | 21731 | 26015 | 16857 | 17431 | 15750 | -0.18183009 | 4.90E-07 |
| BJK46_004560 | 12421 | 13148 | 15622 | 10962 | 10960 | 9955 | -0.16472067 | 2.41E-06 |
| BJK46_004565 | 5616 | 5743 | 6789 | 5803 | 6384 | 5536 | 0.044697919 | 0.21269042 |
| BJK46_004570 | 3357 | 3507 | 4089 | 3394 | 3888 | 3386 | 0.006370681 | 0.19835155 |
| BJK46_004575 | 2105 | 2146 | 2397 | 1960 | 2366 | 2064 | -0.04060019 | 0.15964672 |
| BJK46_004580 | 19457 | 22234 | 23518 | 20583 | 21086 | 20717 | 0.211128308 | 0.21079483 |
| BJK46_004585 | 4002 | 4524 | 4778 | 5066 | 5368 | 5299 | 0.305143925 | 0.01122782 |
| BJK46_004590 | 30536 | 40855 | 24650 | 16143 | 11297 | 14923 | -0.89689318 | 1.28E-10 |
| BJK46_004595 | 3058 | 3512 | 3790 | 4443 | 4533 | 4354 | 0.416658232 | 6.87E-05 |
| BJK46_004600 | 3501 | 3865 | 4249 | 4868 | 5133 | 4739 | 0.401352621 | 0.00013885 |
| BJK46_004605 | 3095 | 3319 | 3746 | 4519 | 4846 | 4323 | 0.48071122 | 1.24E-06 |
| BJK46_004610 | 9367 | 10387 | 11896 | 11500 | 12194 | 10995 | 0.320528168 | 0.37141502 |
| BJK46_004615 | 8599 | 9270 | 10519 | 7993 | 8025 | 7451 | -0.13645583 | 4.05E-05 |
| BJK46_004620 | 2931 | 3037 | 3897 | 2584 | 2800 | 2129 | -0.36128646 | 1.43E-05 |
| BJK46_004625 | 3000 | 2853 | 3931 | 2209 | 2460 | 1977 | -0.52243627 | 3.52E-09 |
| BJK46_004630 | 3281 | 3397 | 4012 | 2535 | 2695 | 2172 | -0.49961438 | 9.66E-11 |
| BJK46_004635 | 2044 | 2193 | 2470 | 1810 | 2249 | 1829 | -0.17436512 | 0.00599162 |
| BJK46_004640 | 1060 | 1123 | 1333 | 1143 | 1406 | 1169 | -0.02314007 | 0.89134369 |
| BJK46_004645 | 5608 | 5896 | 6860 | 8123 | 8953 | 7819 | 0.542569012 | 1.23E-06 |
| BJK46_004650 | 2126 | 2236 | 2594 | 3871 | 4427 | 3583 | 0.803679823 | 7.30E-17 |
| BJK46_004655 | 7033 | 7851 | 7890 | 4508 | 5140 | 5389 | -0.52094975 | 1.09E-16 |
| BJK46_004660 | 12409 | 14099 | 14358 | 8825 | 9824 | 10370 | -0.3040337 | 3.73E-10 |
| BJK46_004665 | 3203 | 3420 | 3762 | 4533 | 5349 | 5077 | 0.577305283 | 8.27E-10 |
| BJK46_004670 | 1004 | 1259 | 918 | 1282 | 1373 | 1657 | 0.346503393 | 0.02157748 |
| BJK46_004675 | 2292 | 2380 | 2655 | 1028 | 1228 | 1088 | -1.14350321 | 1.80E-37 |
| BJK46_004680 | 3195 | 3247 | 3456 | 1288 | 1415 | 1325 | -1.28004211 | 1.14E-58 |
| BJK46_004685 | 3256 | 3292 | 3453 | 1257 | 1588 | 1324 | -1.25066429 | 2.22E-49 |
| BJK46_004690 | 2888 | 2855 | 2991 | 1078 | 1357 | 1229 | -1.25035587 | 8.31E-44 |
| BJK46_004695 | 7464 | 7181 | 8066 | 2627 | 3316 | 2741 | -1.33470793 | 4.35E-69 |
| BJK46_004700 | 3061 | 2963 | 3289 | 1173 | 1461 | 1240 | -1.25679885 | 1.71E-46 |
| BJK46_004705 | 3266 | 3350 | 3592 | 1370 | 1634 | 1494 | -1.16437316 | 1.13E-50 |
| BJK46_004710 | 1985 | 2062 | 2258 | 816 | 1015 | 837 | -1.30495353 | 3.98E-32 |
| BJK46_004715 | 2405 | 2446 | 2684 | 1189 | 1544 | 1335 | -0.89013617 | 9.35E-24 |
| BJK46_004720 | 4919 | 6181 | 5796 | 3091 | 3033 | 3227 | -0.7957302 | 3.35E-25 |
| BJK46_004725 | 10421 | 11279 | 12890 | 7384 | 7472 | 7390 | -0.48335494 | 8.77E-18 |
| BJK46_004730 | 4069 | 4422 | 5166 | 2678 | 2837 | 2720 | -0.68195331 | 8.70E-24 |
| BJK46_004735 | 25102 | 27958 | 33742 | 13077 | 13932 | 11743 | -0.89491497 | 1.23E-29 |
| BJK46_004740 | 36699 | 41505 | 36359 | 32805 | 33594 | 41076 | 0.2294882 | 0.21732677 |
| BJK46_004745 | 5777 | 6314 | 6351 | 5847 | 6799 | 6910 | 0.164930045 | 0.79780208 |
| BJK46_004750 | 73 | 67 | 48 | 71 | 160 | 109 | -0.60630031 | 0.01617424 |
| BJK46_004755 | 297 | 314 | 340 | 419 | 497 | 471 | -0.07416843 | 0.01039905 |
| BJK46_004760 | 689 | 684 | 813 | 900 | 1263 | 989 | 0.278423618 | 0.00288303 |
| BJK46_004765 | 472 | 520 | 507 | 470 | 720 | 504 | -0.31709842 | 0.60034153 |
| BJK46_004770 | 621 | 739 | 816 | 650 | 801 | 695 | -0.34277678 | 0.61701676 |
| BJK46_004775 | 14580 | 16173 | 16604 | 25288 | 21751 | 24595 | 0.858146651 | 1.34E-06 |
| BJK46_004780 | 7367 | 8100 | 7904 | 13580 | 11619 | 13702 | 0.896394174 | 4.26E-11 |
| BJK46_004785 | 6012 | 6481 | 7530 | 6888 | 6298 | 5589 | -0.00654281 | 0.11317091 |
| BJK46_004790 | 14146 | 15016 | 17414 | 16158 | 16740 | 15975 | 0.315880344 | 0.9105722 |
| BJK46_004795 | 13280 | 14519 | 15812 | 14240 | 15447 | 14867 | 0.264522392 | 0.7446103 |
| BJK46_004800 | 6997 | 7253 | 8801 | 5905 | 6451 | 5642 | -0.25935377 | 1.34E-07 |
| BJK46_004805 | 62197 | 64942 | 79259 | 57738 | 55889 | 51049 | 0.063916921 | 0.00063296 |
| BJK46_004810 | 37923 | 43302 | 50057 | 40113 | 39642 | 37521 | 0.184738748 | 0.04426351 |
| BJK46_004815 | 5366 | 5573 | 6384 | 6171 | 6475 | 5501 | 0.142618223 | 0.93940864 |
| BJK46_004820 | 6750 | 7029 | 7840 | 8527 | 8555 | 7814 | 0.320063125 | 0.07195475 |
| BJK46_004825 | 3199 | 3529 | 3589 | 4839 | 4568 | 4378 | 0.467261415 | 1.90E-05 |
| BJK46_004830 | 18439 | 18990 | 18702 | 27423 | 26210 | 28314 | 0.821726262 | 7.44E-06 |
| BJK46_004835 | 11790 | 12848 | 13293 | 15725 | 16729 | 17654 | 0.631208149 | 0.00039936 |
| BJK46_004840 | 17745 | 20546 | 23426 | 28434 | 30881 | 30883 | 0.840880765 | 1.68E-06 |
| BJK46_004845 | 11997 | 15462 | 16258 | 26002 | 25313 | 27117 | 1.117185973 | 6.38E-13 |
| BJK46_004850 | 4910 | 5393 | 5903 | 9355 | 9622 | 9011 | 0.891920663 | 1.31E-21 |
| BJK46_004855 | 5616 | 6211 | 5410 | 9350 | 9653 | 12373 | 0.969594293 | 1.17E-11 |
| BJK46_004860 | 3561 | 3834 | 4205 | 1971 | 2061 | 2017 | -0.90805426 | 3.57E-36 |
| BJK46_004865 | 101 | 118 | 95 | 85 | 122 | 106 | -1.287955 | 0.77415004 |
| BJK46_004870 | 51 | 66 | 47 | 58 | 96 | 65 | -1.14962293 | 0.24911812 |
| BJK46_004875 | 2380 | 2674 | 2360 | 7033 | 6231 | 7183 | 1.513387584 | 6.00E-42 |
| BJK46_004880 | 1355 | 1243 | 1260 | 1561 | 1614 | 1435 | 0.20576239 | 0.13285675 |
| BJK46_004890 | 3017 | 3505 | 2715 | 4190 | 4262 | 4805 | 0.570105241 | 5.66E-05 |
| BJK46_004895 | 93 | 119 | 65 | 156 | 270 | 271 | 0.214938947 | 3.23E-05 |
| BJK46_004900 | 2443 | 2949 | 3090 | 4339 | 4115 | 4521 | 0.659737129 | 3.69E-10 |
| BJK46_004905 | 1544 | 1829 | 2007 | 2570 | 2657 | 2767 | 0.597821973 | 1.61E-08 |
| BJK46_004910 | 240 | 269 | 281 | 273 | 314 | 290 | -0.6192246 | 0.66431853 |
| BJK46_004915 | 241 | 308 | 328 | 427 | 723 | 474 | 0.255442857 | 0.00017876 |
| BJK46_004920 | 1265 | 1714 | 1284 | 1839 | 1667 | 2040 | 0.373243448 | 0.02455716 |
| BJK46_004925 | 21 | 28 | 25 | 60 | 129 | 71 | 0.00730931 | 2.62E-07 |
| BJK46_004930 | 42 | 66 | 49 | 93 | 200 | 118 | -0.06907728 | 2.38E-05 |
| BJK46_004935 | 721 | 837 | 626 | 619 | 768 | 862 | -0.2771482 | 0.87740564 |
| BJK46_004940 | 242 | 246 | 257 | 194 | 213 | 169 | -1.26658409 | 0.04378996 |
| BJK46_004945 | 3002 | 3427 | 3503 | 5509 | 5728 | 5989 | 0.850743941 | 1.54E-21 |
| BJK46_004950 | 2652 | 2952 | 3214 | 5249 | 5686 | 5413 | 0.943104546 | 3.17E-29 |
| BJK46_004955 | 1259 | 1422 | 1560 | 2739 | 3016 | 2621 | 0.997339906 | 2.31E-25 |
| BJK46_004960 | 24632 | 26864 | 30944 | 13731 | 13064 | 12201 | -0.81605533 | 1.19E-26 |
| BJK46_004965 | 42064 | 45455 | 52342 | 23293 | 23054 | 21630 | -0.72303418 | 3.70E-25 |
| BJK46_004970 | 13221 | 13962 | 15429 | 6569 | 6911 | 7127 | -0.88501415 | 9.64E-42 |
| BJK46_004975 | 2857 | 3033 | 3303 | 4039 | 4623 | 3945 | 0.496552481 | 3.10E-07 |
| BJK46_004980 | 53981 | 60599 | 42483 | 66911 | 72653 | 94030 | 0.959487156 | 0.00251231 |
| BJK46_004985 | 5904 | 6684 | 7144 | 6255 | 6774 | 6045 | 0.039334832 | 0.13150569 |
| BJK46_004990 | 2686 | 3024 | 3216 | 3580 | 4013 | 3479 | 0.348953238 | 0.00136601 |
| BJK46_004995 | 2556 | 2820 | 2975 | 3453 | 3693 | 3303 | 0.357796736 | 0.00075983 |
| BJK46_005000 | 1387 | 1666 | 1653 | 2320 | 2293 | 2079 | 0.518621096 | 3.87E-06 |
| BJK46_005005 | 801 | 1026 | 1017 | 565 | 817 | 625 | -0.7939719 | 0.00058613 |
| BJK46_005010 | 236 | 270 | 287 | 139 | 288 | 184 | -1.30696591 | 0.0730511 |
| BJK46_005015 | 10152 | 10939 | 13125 | 5308 | 5866 | 5022 | -0.95650264 | 1.02E-41 |
| BJK46_005020 | 3939 | 4180 | 5123 | 2141 | 2432 | 1989 | -0.97548784 | 1.44E-33 |
| BJK46_005025 | 2921 | 2858 | 3401 | 3930 | 4422 | 4308 | 0.508300964 | 5.47E-07 |
| BJK46_005030 | 16168 | 19524 | 19855 | 9017 | 9210 | 9822 | -0.7664409 | 2.76E-27 |
| BJK46_005035 | 31516 | 38111 | 42299 | 18496 | 17703 | 17935 | -0.74821163 | 9.73E-24 |
| BJK46_005040 | 807 | 839 | 957 | 1209 | 1698 | 1353 | 0.562203784 | 4.01E-06 |
| BJK46_005045 | 3471 | 4274 | 3952 | 4926 | 4855 | 5318 | 0.427192197 | 0.00049432 |
| BJK46_005050 | 2163 | 2542 | 2721 | 2569 | 2621 | 2536 | 0.087946624 | 0.99818149 |
| BJK46_005055 | 1550 | 1602 | 1826 | 2018 | 2338 | 2080 | 0.379903237 | 0.00055485 |
| BJK46_005060 | 899 | 962 | 1046 | 903 | 1052 | 1002 | -0.16461059 | 0.78111974 |
| BJK46_005065 | 3344 | 3365 | 3635 | 4212 | 4399 | 4038 | 0.336335536 | 0.00338799 |
| BJK46_005070 | 2076 | 2233 | 2360 | 2433 | 2532 | 2463 | 0.179035842 | 0.25177708 |
| BJK46_005075 | 2627 | 3274 | 3231 | 3951 | 4157 | 3970 | 0.450535198 | 2.39E-05 |
| BJK46_005080 | 7482 | 8494 | 8235 | 3931 | 3908 | 4590 | -0.8912299 | 5.04E-29 |
| BJK46_005085 | 13798 | 15575 | 16037 | 7686 | 7315 | 7649 | -0.82418949 | 3.05E-33 |
| BJK46_005090 | 8432 | 9551 | 10281 | 11099 | 10976 | 12010 | 0.443712125 | 0.01350311 |
| BJK46_005095 | 5973 | 6476 | 7261 | 6715 | 7310 | 6447 | 0.147630024 | 0.9354724 |
| BJK46_005100 | 3849 | 4098 | 4322 | 5245 | 6212 | 5642 | 0.536797826 | 7.39E-09 |
| BJK46_005105 | 22954 | 26775 | 24356 | 54855 | 57956 | 72076 | 1.644261393 | 3.46E-22 |
| BJK46_005110 | 9121 | 9499 | 10900 | 4479 | 4581 | 4096 | -1.07595451 | 1.75E-51 |
| BJK46_005115 | 8589 | 9041 | 10539 | 4971 | 5195 | 4452 | -0.8519357 | 1.10E-33 |
| BJK46_005120 | 6376 | 7257 | 8320 | 5815 | 5959 | 5211 | -0.27932438 | 1.77E-07 |
| BJK46_005125 | 2724 | 2559 | 2378 | 1469 | 1446 | 1285 | -0.8582475 | 4.02E-16 |
| BJK46_005130 | 32812 | 34620 | 32970 | 20286 | 17075 | 19365 | -0.54043093 | 3.62E-13 |
| BJK46_005135 | 518 | 634 | 665 | 189 | 259 | 197 | -2.14508883 | 4.02E-15 |
| BJK46_005140 | 5164 | 5375 | 6119 | 1835 | 2345 | 1889 | -1.4239028 | 1.97E-73 |
| BJK46_005145 | 776 | 763 | 895 | 289 | 388 | 284 | -1.83701538 | 2.04E-14 |
| BJK46_005150 | 9341 | 9843 | 10083 | 11927 | 11215 | 13097 | 0.485640635 | 0.00944112 |
| BJK46_005155 | 5708 | 5812 | 6297 | 4212 | 4465 | 4391 | -0.38338583 | 1.58E-12 |
| BJK46_005160 | 161 | 150 | 148 | 255 | 456 | 352 | 0.306163003 | 3.03E-06 |
| BJK46_005165 | 32 | 41 | 36 | 44 | 64 | 50 | -1.21253669 | 0.12078873 |
| BJK46_005170 | 7680 | 9440 | 7960 | 12103 | 12725 | 15429 | 0.854600409 | 4.39E-08 |
| BJK46_005175 | 4227 | 4518 | 4949 | 5676 | 6062 | 6180 | 0.454574489 | 7.56E-06 |
| BJK46_005180 | 10403 | 11341 | 11964 | 13038 | 12319 | 12001 | 0.34578774 | 0.31752372 |
| BJK46_005185 | 6624 | 6957 | 7414 | 13294 | 13878 | 13914 | 1.129040697 | 6.14E-30 |
| BJK46_005190 | 2314 | 2590 | 2766 | 19060 | 21007 | 20979 | 3.097516084 | 4.59E-246 |
| BJK46_005195 | 324 | 302 | 342 | 224 | 398 | 234 | -0.96163574 | 0.27689143 |
| BJK46_005200 | 1298 | 1372 | 1530 | 35728 | 36516 | 39123 | 4.833336021 | 0 |
| BJK46_005205 | 1178 | 1146 | 1260 | 369 | 650 | 433 | -1.64984053 | 8.03E-14 |
| BJK46_005210 | 1628 | 1541 | 1780 | 515 | 927 | 580 | -1.50008674 | 5.88E-15 |
| BJK46_005215 | 1042 | 930 | 1144 | 337 | 723 | 384 | -1.53454924 | 2.48E-07 |
| BJK46_005220 | 1517 | 1534 | 1767 | 562 | 1164 | 664 | -1.21834294 | 2.53E-08 |
| BJK46_005225 | 1327 | 1347 | 1646 | 503 | 1131 | 586 | -1.22648707 | 2.41E-06 |
| BJK46_005230 | 1028 | 971 | 1268 | 450 | 1020 | 530 | -1.0568421 | 0.00064369 |
| BJK46_005235 | 1907 | 1849 | 2238 | 641 | 1236 | 676 | -1.37608243 | 9.94E-13 |
| BJK46_005240 | 908 | 983 | 1191 | 360 | 660 | 366 | -1.55839654 | 6.12E-09 |
| BJK46_005245 | 1001 | 1152 | 1318 | 361 | 648 | 395 | -1.6691622 | 1.72E-12 |
| BJK46_005250 | 51191 | 57157 | 51549 | 17685 | 13863 | 17616 | -1.40614869 | 3.55E-36 |
| BJK46_005255 | 10854 | 11195 | 11651 | 4425 | 3918 | 3896 | -1.37077708 | 4.06E-59 |
| BJK46_005260 | 5985 | 5959 | 6416 | 2418 | 2575 | 2365 | -1.27210753 | 1.04E-69 |
| BJK46_005265 | 3631 | 3871 | 3980 | 5306 | 5430 | 5291 | 0.538143323 | 2.35E-08 |
| BJK46_005270 | 142453 | 156208 | 167350 | 125994 | 109704 | 114463 | 0.049866712 | 5.44E-05 |
| BJK46_005275 | 135337 | 145900 | 162109 | 120128 | 108030 | 112982 | 0.082791996 | 0.00012285 |
| BJK46_005280 | 205177 | 222923 | 238784 | 184046 | 165627 | 183999 | 0.171858392 | 0.00085985 |
| BJK46_005285 | 906 | 875 | 1033 | 694 | 833 | 816 | -0.51224835 | 0.0282654 |
| BJK46_005290 | 869 | 852 | 988 | 701 | 818 | 758 | -0.51206922 | 0.03436175 |
| BJK46_005295 | 1138 | 1111 | 1252 | 842 | 972 | 829 | -0.58067615 | 0.00061535 |
| BJK46_005300 | 2275 | 2206 | 2356 | 1653 | 2040 | 1643 | -0.35079118 | 9.38E-06 |
| BJK46_005305 | 593 | 679 | 636 | 423 | 572 | 460 | -0.84616196 | 0.01016898 |
| BJK46_005310 | 835 | 886 | 914 | 552 | 734 | 605 | -0.79870647 | 0.00063058 |
| BJK46_005315 | 3094 | 3109 | 3270 | 1993 | 2702 | 2099 | -0.46573157 | 8.10E-09 |
| BJK46_005320 | 1639 | 1636 | 1647 | 838 | 1041 | 958 | -0.89149536 | 2.69E-12 |
| BJK46_005325 | 381 | 419 | 336 | 410 | 537 | 668 | -0.06020781 | 0.05066987 |
| BJK46_005330 | 395 | 402 | 359 | 391 | 522 | 614 | -0.17397349 | 0.12324312 |
| BJK46_005335 | 1347 | 1727 | 1371 | 2255 | 2525 | 2936 | 0.807664696 | 4.00E-09 |
| BJK46_005340 | 5322 | 5301 | 5531 | 6848 | 6729 | 7287 | 0.447702198 | 0.00026219 |
| BJK46_005345 | 13887 | 16722 | 12365 | 6038 | 6573 | 9170 | -0.82822166 | 1.78E-11 |
| BJK46_005350 | 4843 | 5217 | 4958 | 4338 | 4197 | 4505 | -0.14573467 | 0.00173773 |
| BJK46_005355 | 1967 | 1939 | 2000 | 1802 | 1936 | 1656 | -0.12514481 | 0.04225272 |
| BJK46_005360 | 1558 | 1667 | 1589 | 1604 | 1612 | 1402 | -0.08361043 | 0.26242888 |
| BJK46_005365 | 1190 | 1411 | 1297 | 1484 | 1511 | 1347 | 0.098785215 | 0.44383142 |
| BJK46_005370 | 1937 | 2022 | 2048 | 2095 | 2073 | 1858 | 0.016387482 | 0.53913236 |
| BJK46_005375 | 5883 | 6386 | 6098 | 7579 | 6594 | 6911 | 0.284677895 | 0.13946583 |
| BJK46_005380 | 969 | 1177 | 1127 | 1109 | 1368 | 1243 | 0.067295272 | 0.35826749 |
| BJK46_005385 | 1437 | 1628 | 1708 | 1486 | 1927 | 1681 | 0.077267517 | 0.78376812 |
| BJK46_005390 | 11958 | 12641 | 15208 | 8562 | 9791 | 8408 | -0.38895713 | 9.37E-14 |
| BJK46_005395 | 3494 | 3672 | 4632 | 2424 | 2790 | 2343 | -0.60205522 | 2.42E-15 |
| BJK46_005400 | 3403 | 3731 | 4328 | 2988 | 3132 | 2841 | -0.31092973 | 3.35E-07 |
| BJK46_005405 | 7155 | 7713 | 9271 | 6397 | 6868 | 5754 | -0.23831642 | 2.68E-06 |
| BJK46_005410 | 2883 | 3045 | 3459 | 2805 | 2979 | 2622 | -0.12614048 | 0.00707409 |
| BJK46_005415 | 2215 | 2445 | 2314 | 3822 | 4961 | 5622 | 1.066549944 | 8.10E-18 |
| BJK46_005420 | 44 | 43 | 34 | 156 | 415 | 226 | 1.297827954 | 2.96E-14 |
| BJK46_005425 | 45 | 42 | 50 | 143 | 299 | 182 | 0.790063963 | 2.10E-12 |
| BJK46_005430 | 52 | 60 | 56 | 209 | 426 | 246 | 1.137962372 | 2.64E-15 |
| BJK46_005435 | 69 | 75 | 73 | 207 | 449 | 236 | 0.837489744 | 3.40E-11 |
| BJK46_005440 | 90 | 104 | 98 | 340 | 621 | 398 | 1.24923295 | 2.35E-17 |
| BJK46_005445 | 199 | 207 | 236 | 573 | 869 | 644 | 1.062717084 | 9.21E-16 |
| BJK46_005450 | 1810 | 1935 | 1981 | 893 | 1076 | 977 | -1.02050759 | 1.67E-20 |
| BJK46_005455 | 1047 | 1114 | 1212 | 448 | 637 | 560 | -1.33531253 | 2.63E-12 |
| BJK46_005460 | 5041 | 5261 | 5918 | 3924 | 4106 | 3912 | -0.38115036 | 4.76E-12 |
| BJK46_005465 | 1114 | 1176 | 1259 | 1635 | 1737 | 1724 | 0.475399867 | 2.31E-05 |
| BJK46_005470 | 2067 | 2048 | 2237 | 2455 | 2912 | 2807 | 0.383742595 | 0.00052953 |
| BJK46_005475 | 356 | 451 | 393 | 414 | 523 | 496 | -0.30098783 | 0.31984264 |
| BJK46_005480 | 446 | 527 | 514 | 447 | 615 | 554 | -0.36831837 | 0.77637343 |
| BJK46_005485 | 8977 | 10049 | 9114 | 22066 | 20638 | 22287 | 1.435320221 | 9.02E-26 |
| BJK46_005490 | 18287 | 18773 | 22779 | 15178 | 16329 | 14508 | -0.12216063 | 8.95E-06 |
| BJK46_005495 | 12197 | 11956 | 14763 | 9516 | 9842 | 8565 | -0.29315097 | 3.89E-09 |
| BJK46_005500 | 121 | 158 | 115 | 221 | 303 | 286 | 0.068858294 | 5.51E-05 |
| BJK46_005505 | 25 | 38 | 39 | 84 | 84 | 102 | -0.19808317 | 3.97E-06 |
| BJK46_005510 | 482 | 576 | 494 | 798 | 882 | 884 | 0.364981243 | 9.44E-05 |
| BJK46_005515 | 406 | 509 | 432 | 665 | 709 | 771 | 0.238766426 | 0.00080851 |
| BJK46_005520 | 1284 | 1440 | 1522 | 737 | 915 | 859 | -0.90411058 | 3.74E-10 |
| BJK46_005525 | 1806 | 2155 | 2259 | 1225 | 1639 | 1242 | -0.61359506 | 7.89E-10 |
| BJK46_005530 | 587 | 683 | 744 | 486 | 651 | 465 | -0.75233547 | 0.02843854 |
| BJK46_005535 | 1250 | 1246 | 1285 | 1587 | 2104 | 1876 | 0.520894276 | 1.58E-05 |
| BJK46_005540 | 169 | 183 | 190 | 267 | 453 | 374 | 0.165593626 | 3.69E-05 |
| BJK46_005545 | 1535 | 1751 | 1887 | 2261 | 2464 | 2311 | 0.461503771 | 8.83E-06 |
| BJK46_005550 | 2240 | 2232 | 2629 | 2365 | 2867 | 2297 | 0.104691914 | 0.83146044 |
| BJK46_005555 | 194 | 219 | 162 | 405 | 558 | 504 | 0.621908397 | 1.08E-08 |
| BJK46_005560 | 9136 | 9695 | 10080 | 3841 | 3975 | 3856 | -1.22819795 | 5.07E-75 |
| BJK46_005565 | 2924 | 3218 | 3454 | 4247 | 4367 | 3977 | 0.437451452 | 1.78E-05 |
| BJK46_005570 | 4180 | 4320 | 4563 | 5812 | 6317 | 5979 | 0.53453976 | 8.77E-09 |
| BJK46_005575 | 5041 | 5013 | 5592 | 6583 | 7365 | 6335 | 0.448821989 | 4.47E-05 |
| BJK46_005580 | 7612 | 7799 | 8688 | 7902 | 8187 | 7409 | 0.084087649 | 0.20625904 |
| BJK46_005585 | 686 | 754 | 890 | 694 | 874 | 723 | -0.31952309 | 0.58345974 |
| BJK46_005590 | 962 | 997 | 1327 | 1097 | 1413 | 1138 | 0.034915699 | 0.52806179 |
| BJK46_005595 | 2308 | 2408 | 2965 | 2713 | 3288 | 2706 | 0.210573017 | 0.1853069 |
| BJK46_005600 | 1468 | 1561 | 1987 | 2041 | 2584 | 1895 | 0.387024695 | 0.00459871 |
| BJK46_005605 | 5551 | 6245 | 6222 | 12157 | 12912 | 14035 | 1.256796004 | 1.78E-34 |
| BJK46_005610 | 107 | 88 | 101 | 101 | 232 | 142 | -0.59229506 | 0.04323476 |
| BJK46_005615 | 29 | 34 | 31 | 47 | 106 | 62 | -0.60056553 | 0.00065986 |
| BJK46_005620 | 5897 | 6370 | 6436 | 4139 | 4230 | 4269 | -0.501123 | 2.93E-17 |
| BJK46_005625 | 11213 | 14753 | 12235 | 13762 | 11113 | 14429 | 0.252018892 | 0.91572413 |
| BJK46_005630 | 10286 | 12743 | 13081 | 8486 | 8697 | 9936 | -0.23727332 | 7.82E-07 |
| BJK46_005635 | 5452 | 6336 | 7481 | 3855 | 4287 | 4586 | -0.52549277 | 9.34E-14 |
| BJK46_005640 | 12207 | 13812 | 14920 | 4104 | 4216 | 4137 | -1.60161625 | 2.21E-108 |
| BJK46_005645 | 52988 | 79501 | 32337 | 50324 | 31062 | 68002 | 0.239874502 | 0.49556285 |
| BJK46_005650 | 394 | 428 | 442 | 382 | 483 | 433 | -0.53306747 | 0.88239111 |
| BJK46_005655 | 660 | 759 | 782 | 731 | 879 | 766 | -0.18996134 | 0.76552625 |
| BJK46_005660 | 652 | 724 | 759 | 735 | 892 | 871 | -0.07116932 | 0.29184864 |
| BJK46_005665 | 3003 | 3523 | 3022 | 7754 | 8293 | 8662 | 1.437337682 | 7.51E-47 |
| BJK46_005670 | 889 | 966 | 1061 | 1071 | 1459 | 1190 | 0.201236879 | 0.03843151 |
| BJK46_005675 | 3479 | 4077 | 4799 | 4495 | 4306 | 3659 | 0.069678444 | 0.6763615 |
| BJK46_005680 | 3875 | 4368 | 4939 | 5073 | 5229 | 4733 | 0.253662192 | 0.08766317 |
| BJK46_005685 | 779 | 924 | 944 | 3213 | 3190 | 2904 | 1.806640158 | 1.33E-72 |
| BJK46_005690 | 844 | 875 | 980 | 3088 | 3344 | 2620 | 1.729267029 | 3.12E-59 |
| BJK46_005695 | 1532 | 1498 | 1681 | 3956 | 4697 | 3835 | 1.426943818 | 4.84E-53 |
| BJK46_005700 | 381 | 383 | 502 | 959 | 1417 | 1076 | 1.094445948 | 2.92E-15 |
| BJK46_005705 | 3930 | 4374 | 5220 | 5948 | 6265 | 5882 | 0.493844973 | 2.77E-06 |
| BJK46_005710 | 6596 | 7188 | 8601 | 9403 | 10239 | 9327 | 0.510574354 | 0.00010896 |
| BJK46_005715 | 9081 | 10504 | 11055 | 12676 | 12295 | 12239 | 0.471424611 | 0.00951987 |
| BJK46_005720 | 176 | 166 | 159 | 247 | 400 | 295 | 0.010886285 | 0.00040716 |
| BJK46_005725 | 515 | 566 | 584 | 786 | 1212 | 862 | 0.432279189 | 5.70E-05 |
| BJK46_005730 | 57 | 69 | 74 | 120 | 209 | 124 | -0.16816393 | 6.63E-05 |
| BJK46_005735 | 5686 | 6270 | 6341 | 13162 | 13068 | 12881 | 1.238621239 | 8.41E-37 |
| BJK46_005740 | 782 | 852 | 985 | 870 | 1156 | 953 | -0.02920941 | 0.40675557 |
| BJK46_005745 | 1722 | 2209 | 2287 | 1752 | 2120 | 2019 | -0.0577765 | 0.15924846 |
| BJK46_005750 | 3811 | 4392 | 4698 | 2625 | 2907 | 2551 | -0.63264373 | 4.07E-21 |
| BJK46_005755 | 1675 | 1915 | 1927 | 2281 | 2412 | 2357 | 0.372322746 | 0.00072704 |
| BJK46_005760 | 1029 | 1088 | 1320 | 2000 | 2085 | 2146 | 0.84008472 | 4.34E-14 |
| BJK46_005765 | 4191 | 4464 | 4990 | 2121 | 2316 | 1970 | -1.0566501 | 8.01E-46 |
| BJK46_005770 | 491 | 599 | 612 | 188 | 251 | 200 | -2.0793305 | 7.01E-14 |
| BJK46_005775 | 217 | 267 | 223 | 344 | 529 | 404 | 0.124755509 | 0.00034749 |
| BJK46_005780 | 4469 | 5243 | 5232 | 2249 | 2257 | 2158 | -1.12400339 | 7.38E-52 |
| BJK46_005785 | 26689 | 30060 | 33152 | 13718 | 14603 | 12991 | -0.85158375 | 3.98E-32 |
| BJK46_005790 | 37726 | 41325 | 46243 | 35957 | 37112 | 32116 | 0.080170417 | 0.00326564 |
| BJK46_005795 | 2594 | 2808 | 3317 | 4126 | 4768 | 4184 | 0.630779235 | 3.68E-11 |
| BJK46_005800 | 1050 | 1133 | 1372 | 1358 | 1855 | 1572 | 0.366699625 | 0.00364878 |
| BJK46_005805 | 188653 | 206668 | 248591 | 143581 | 145692 | 126989 | -0.14095166 | 1.27E-09 |
| BJK46_005810 | 197314 | 211007 | 275719 | 94733 | 91641 | 76166 | -0.90745808 | 2.31E-28 |
| BJK46_005815 | 67449 | 70767 | 92108 | 28053 | 29174 | 24583 | -1.13335788 | 8.53E-38 |
| BJK46_005820 | 52110 | 58472 | 72642 | 19955 | 20888 | 17717 | -1.31643696 | 2.09E-47 |
| BJK46_005825 | 31758 | 37546 | 45960 | 13749 | 15044 | 13587 | -1.14967945 | 8.99E-41 |
| BJK46_005830 | 69427 | 74599 | 88721 | 42463 | 41073 | 35499 | -0.59200542 | 1.13E-18 |
| BJK46_005835 | 57356 | 61111 | 72907 | 33150 | 33454 | 28748 | -0.65259876 | 3.93E-21 |
| BJK46_005840 | 4472 | 4815 | 5991 | 1918 | 2223 | 1818 | -1.31792054 | 2.13E-55 |
| BJK46_005845 | 29248 | 32473 | 37520 | 11767 | 11800 | 10924 | -1.25580002 | 1.40E-51 |
| BJK46_005850 | 49634 | 56149 | 63571 | 19351 | 19549 | 19056 | -1.22541318 | 9.57E-51 |
| BJK46_005855 | 52930 | 59400 | 66288 | 26251 | 22940 | 21346 | -1.01200561 | 2.10E-30 |
| BJK46_005860 | 44163 | 49988 | 57173 | 24848 | 25669 | 25750 | -0.6576549 | 4.52E-23 |
| BJK46_005865 | 10680 | 11970 | 13554 | 8426 | 9368 | 7696 | -0.33872747 | 3.56E-11 |
| BJK46_005870 | 4884 | 5440 | 5927 | 4161 | 4586 | 3560 | -0.34597835 | 4.77E-08 |
| BJK46_005875 | 3185 | 3505 | 3882 | 2682 | 3029 | 2541 | -0.32345839 | 1.78E-07 |
| BJK46_005880 | 2538 | 2697 | 3112 | 2072 | 2522 | 1969 | -0.32640856 | 5.93E-06 |
| BJK46_005885 | 2111 | 2296 | 2752 | 1882 | 2105 | 1623 | -0.33395218 | 4.46E-05 |
| BJK46_005890 | 2691 | 2709 | 3288 | 2324 | 2721 | 1934 | -0.29832699 | 0.00031576 |
| BJK46_005895 | 2063 | 2079 | 2363 | 1773 | 2021 | 1704 | -0.23064275 | 0.00069301 |
| BJK46_005900 | 3976 | 4131 | 4998 | 3830 | 4055 | 3391 | -0.16387785 | 0.0013005 |
| BJK46_005905 | 1607 | 1611 | 2080 | 1480 | 1712 | 1423 | -0.20475685 | 0.01715755 |
| BJK46_005910 | 22456 | 23509 | 27597 | 22386 | 23227 | 21512 | 0.1525737 | 0.0565277 |
| BJK46_005915 | 3787 | 4063 | 4648 | 4488 | 4803 | 4355 | 0.184614374 | 0.37686713 |
| BJK46_005920 | 4082 | 4344 | 5077 | 5249 | 5346 | 4688 | 0.241647538 | 0.13342131 |
| BJK46_005925 | 4425 | 4796 | 5273 | 5791 | 5997 | 5301 | 0.30436805 | 0.01788062 |
| BJK46_005930 | 4553 | 4939 | 5511 | 6277 | 6740 | 5546 | 0.376450645 | 0.00205864 |
| BJK46_005935 | 672 | 655 | 722 | 951 | 1192 | 984 | 0.34980763 | 0.00031121 |
| BJK46_005940 | 417 | 449 | 473 | 724 | 893 | 752 | 0.404051958 | 7.10E-06 |
| BJK46_005945 | 525 | 558 | 556 | 941 | 1100 | 958 | 0.555040719 | 1.75E-07 |
| BJK46_005950 | 397 | 385 | 465 | 735 | 987 | 725 | 0.536664457 | 5.48E-07 |
| BJK46_005955 | 637 | 711 | 744 | 1328 | 1670 | 1211 | 0.812150148 | 1.06E-10 |
| BJK46_005960 | 332 | 402 | 362 | 882 | 1101 | 976 | 1.020671796 | 1.47E-15 |
| BJK46_005965 | 13052 | 14267 | 16673 | 48108 | 52506 | 46348 | 2.038255683 | 1.46E-57 |
| BJK46_005970 | 2958 | 3528 | 3674 | 14649 | 15386 | 14383 | 2.233342534 | 3.23E-139 |
| BJK46_005975 | 2069 | 2134 | 2371 | 9316 | 9975 | 8629 | 2.142119927 | 6.88E-147 |
| BJK46_005980 | 1459 | 1536 | 1694 | 7858 | 8694 | 7508 | 2.400145622 | 4.53E-195 |
| BJK46_005985 | 32795 | 35178 | 32469 | 23173 | 19977 | 23591 | -0.29805288 | 1.20E-07 |
| BJK46_005990 | 134 | 128 | 128 | 191 | 409 | 279 | 0.142661054 | 4.95E-05 |
| BJK46_005995 | 21338 | 24084 | 26548 | 11874 | 12159 | 11507 | -0.76501828 | 1.95E-28 |
| BJK46_006000 | 20473 | 23150 | 26706 | 12451 | 12904 | 12040 | -0.65435591 | 1.74E-22 |
| BJK46_006005 | 685 | 782 | 830 | 769 | 1334 | 892 | 0.103686335 | 0.08340012 |
| BJK46_006010 | 626 | 620 | 591 | 623 | 1171 | 774 | 0.103244104 | 0.05169314 |
| BJK46_006025 | 4728 | 5138 | 5230 | 2799 | 3226 | 3433 | -0.62961858 | 2.25E-18 |
| BJK46_006030 | 5106 | 5547 | 6351 | 8389 | 9010 | 7904 | 0.672735839 | 4.94E-11 |
| BJK46_006035 | 233 | 326 | 323 | 310 | 411 | 379 | -0.36735864 | 0.21451904 |
| BJK46_006040 | 1691 | 1958 | 1861 | 783 | 940 | 997 | -1.10170554 | 5.29E-18 |
| BJK46_006045 | 2207 | 2282 | 2681 | 4387 | 5010 | 4126 | 0.952010343 | 2.47E-24 |
| BJK46_006050 | 2071 | 2157 | 2363 | 3843 | 4036 | 3818 | 0.859483992 | 2.65E-22 |
| BJK46_006055 | 4707 | 4945 | 4915 | 3585 | 4047 | 3630 | -0.32025072 | 1.24E-08 |
| BJK46_006060 | 3073 | 3033 | 3575 | 5378 | 6302 | 5246 | 0.857758454 | 3.70E-20 |
| BJK46_006065 | 11153 | 11918 | 13464 | 11378 | 11874 | 11665 | 0.135948568 | 0.13510961 |
| BJK46_006070 | 7236 | 7804 | 7428 | 3804 | 4002 | 3929 | -0.86899676 | 4.90E-37 |
| BJK46_006075 | 611 | 673 | 812 | 893 | 1146 | 1077 | 0.323128658 | 0.00083993 |
| BJK46_006080 | 1292 | 1559 | 1622 | 1730 | 2039 | 1678 | 0.273644482 | 0.03147646 |
| BJK46_006085 | 4918 | 5776 | 6470 | 5372 | 5764 | 5109 | -0.00260321 | 0.07787878 |
| BJK46_006090 | 4220 | 4646 | 5331 | 3630 | 4010 | 3329 | -0.31852495 | 1.04E-07 |
| BJK46_006095 | 3283 | 3560 | 3967 | 2464 | 2784 | 2312 | -0.48420423 | 1.88E-12 |
| BJK46_006100 | 3361 | 3585 | 4094 | 2416 | 2850 | 2500 | -0.47346954 | 4.78E-13 |
| BJK46_006105 | 3074 | 3383 | 3586 | 2341 | 2495 | 2280 | -0.46665164 | 1.07E-12 |
| BJK46_006110 | 3124 | 3594 | 3656 | 5429 | 5686 | 5525 | 0.739072312 | 8.72E-17 |
| BJK46_006115 | 17186 | 19488 | 21643 | 18074 | 18865 | 17966 | 0.178105544 | 0.13332642 |
| BJK46_006120 | 2194 | 2640 | 2821 | 991 | 1174 | 1041 | -1.26214818 | 6.08E-43 |
| BJK46_006125 | 7346 | 8022 | 7875 | 16411 | 16049 | 16740 | 1.269573458 | 1.89E-29 |
| BJK46_006130 | 4681 | 5096 | 5811 | 4183 | 4662 | 4241 | -0.18937889 | 1.40E-05 |
| BJK46_006135 | 2523 | 2692 | 3171 | 2245 | 2541 | 2145 | -0.24727082 | 0.00010008 |
| BJK46_006140 | 114 | 114 | 114 | 186 | 410 | 247 | 0.214969182 | 1.04E-05 |
| BJK46_006145 | 1920 | 2208 | 2520 | 3471 | 3511 | 3186 | 0.647728095 | 2.25E-10 |
| BJK46_006150 | 9079 | 10622 | 10555 | 7945 | 7604 | 7421 | -0.256237 | 7.84E-08 |
| BJK46_006155 | 8190 | 8954 | 9542 | 6269 | 6177 | 6370 | -0.39262551 | 8.02E-13 |
| BJK46_006160 | 13546 | 14646 | 15158 | 9968 | 9566 | 9446 | -0.38393131 | 4.60E-13 |
| BJK46_006165 | 8477 | 9086 | 10889 | 13657 | 15962 | 13439 | 0.794204071 | 1.56E-09 |
| BJK46_006170 | 2007 | 1882 | 2163 | 4310 | 5294 | 4580 | 1.258259069 | 4.55E-41 |
| BJK46_006175 | 953 | 940 | 888 | 1499 | 1672 | 1692 | 0.707724828 | 1.56E-08 |
| BJK46_006180 | 911 | 965 | 962 | 1220 | 1359 | 1363 | 0.339217388 | 0.00176168 |
| BJK46_006185 | 1537 | 1641 | 1697 | 1451 | 1700 | 1630 | -0.04631335 | 0.35516461 |
| BJK46_006190 | 2225 | 2300 | 2548 | 2057 | 2357 | 2176 | -0.08129894 | 0.04767764 |
| BJK46_006195 | 6903 | 7010 | 8651 | 7089 | 7615 | 6735 | 0.037437254 | 0.10499057 |
| BJK46_006200 | 1329 | 1590 | 1677 | 799 | 977 | 805 | -0.95399729 | 3.80E-12 |
| BJK46_006205 | 1819 | 2097 | 2350 | 812 | 1145 | 853 | -1.22012281 | 5.47E-23 |
| BJK46_006210 | 14675 | 16181 | 13274 | 15985 | 17257 | 20911 | 0.534143747 | 0.0819291 |
| BJK46_006215 | 386 | 381 | 492 | 488 | 664 | 493 | -0.13492558 | 0.09555071 |
| BJK46_006220 | 1741 | 1863 | 2080 | 2129 | 2966 | 2247 | 0.375584318 | 0.00310315 |
| BJK46_006225 | 2689 | 2967 | 3539 | 2643 | 3104 | 2426 | -0.13870364 | 0.01226221 |
| BJK46_006230 | 3860 | 4316 | 5024 | 3401 | 3891 | 3297 | -0.26539079 | 2.91E-06 |
| BJK46_006235 | 2490 | 2804 | 2702 | 2111 | 2373 | 2534 | -0.1665032 | 0.00578007 |
| BJK46_006240 | 1210 | 1339 | 1285 | 5618 | 5592 | 5806 | 2.17732233 | 3.79E-126 |
| BJK46_006245 | 1559 | 1671 | 1778 | 1764 | 2016 | 1693 | 0.124022014 | 0.49994371 |
| BJK46_006250 | 995 | 1022 | 1205 | 816 | 1100 | 931 | -0.36080775 | 0.08777035 |
| BJK46_006255 | 1296 | 1275 | 1551 | 1023 | 1418 | 1168 | -0.28024959 | 0.046489 |
| BJK46_006260 | 37 | 23 | 25 | 52 | 102 | 57 | -0.47298357 | 0.00035683 |
| BJK46_006265 | 2448 | 2609 | 2694 | 4582 | 5079 | 5192 | 0.979044607 | 8.25E-29 |
| BJK46_006270 | 1132 | 1273 | 1097 | 860 | 1031 | 1077 | -0.39200439 | 0.03699341 |
| BJK46_006275 | 12810 | 13880 | 13974 | 12352 | 12159 | 13160 | 0.103002802 | 0.06564537 |
| BJK46_006280 | 681 | 779 | 762 | 3487 | 3969 | 3773 | 2.328055536 | 3.13E-139 |
| BJK46_006285 | 10504 | 11271 | 11430 | 18357 | 18269 | 20933 | 1.021383845 | 2.27E-12 |
| BJK46_006290 | 36112 | 40594 | 41819 | 21736 | 21746 | 22477 | -0.5403364 | 1.50E-18 |
| BJK46_006295 | 2380 | 2667 | 2863 | 5513 | 5891 | 5413 | 1.137648435 | 1.22E-42 |
| BJK46_006300 | 3138 | 3565 | 3821 | 13496 | 13941 | 13679 | 2.066676887 | 1.00E-124 |
| BJK46_006305 | 64 | 75 | 75 | 9975 | 7609 | 10064 | 6.724031497 | 0 |
| BJK46_006310 | 253 | 257 | 253 | 471 | 966 | 609 | 0.769447892 | 2.24E-08 |
| BJK46_006315 | 6006 | 6761 | 7155 | 2080 | 2510 | 2206 | -1.50515335 | 2.97E-99 |
| BJK46_006320 | 3164 | 3424 | 3693 | 2211 | 2533 | 2295 | -0.51722757 | 3.82E-15 |
| BJK46_006325 | 168 | 145 | 145 | 315 | 778 | 465 | 0.887903019 | 5.23E-09 |
| BJK46_006330 | 1349 | 1401 | 1404 | 1614 | 1829 | 1817 | 0.315276684 | 0.00910464 |
| BJK46_006335 | 1430 | 1602 | 1671 | 1722 | 2143 | 1871 | 0.280271741 | 0.02096687 |
| BJK46_006340 | 1482 | 1577 | 1575 | 3609 | 3555 | 3963 | 1.284952592 | 1.15E-37 |
| BJK46_006345 | 6589 | 6962 | 7570 | 3995 | 4519 | 3974 | -0.69080053 | 2.57E-30 |
| BJK46_006350 | 442 | 458 | 470 | 318 | 769 | 453 | -0.44930289 | 0.74744349 |
| BJK46_006355 | 79 | 108 | 123 | 142 | 421 | 194 | 0.076146872 | 0.00031902 |
| BJK46_006360 | 113 | 114 | 113 | 217 | 572 | 299 | 0.605477723 | 1.72E-07 |
| BJK46_006365 | 122 | 105 | 117 | 201 | 510 | 264 | 0.415441501 | 3.24E-06 |
| BJK46_006370 | 86 | 88 | 101 | 157 | 393 | 214 | 0.273951003 | 4.45E-06 |
| BJK46_006375 | 125 | 137 | 137 | 259 | 689 | 349 | 0.709560379 | 7.82E-08 |
| BJK46_006380 | 93 | 103 | 116 | 173 | 460 | 238 | 0.331285732 | 5.33E-06 |
| BJK46_006385 | 242 | 266 | 301 | 355 | 675 | 472 | 0.203446153 | 0.00032444 |
| BJK46_006390 | 265 | 292 | 269 | 712 | 808 | 805 | 0.960911279 | 6.70E-15 |
| BJK46_006395 | 969 | 1133 | 1081 | 1453 | 1664 | 1481 | 0.448220429 | 8.56E-05 |
| BJK46_006400 | 1143 | 1162 | 1327 | 743 | 1259 | 885 | -0.51528821 | 0.01358065 |
| BJK46_006405 | 1109 | 1095 | 1308 | 713 | 1072 | 737 | -0.6846045 | 0.00060785 |
| BJK46_006410 | 3051 | 3093 | 3323 | 4922 | 5319 | 4947 | 0.730936321 | 2.74E-16 |
| BJK46_006415 | 3577 | 3667 | 3935 | 5791 | 5887 | 5511 | 0.678588099 | 1.30E-13 |
| BJK46_006420 | 606 | 527 | 633 | 500 | 837 | 641 | -0.2605397 | 0.66308847 |
| BJK46_006425 | 3117 | 3159 | 3427 | 1892 | 2152 | 2093 | -0.6365868 | 1.31E-18 |
| BJK46_006430 | 2561 | 2664 | 2913 | 1687 | 1873 | 1895 | -0.55719397 | 1.22E-13 |
| BJK46_006435 | 14981 | 16607 | 17932 | 4335 | 4168 | 4146 | -1.83701595 | 1.16E-120 |
| BJK46_006440 | 5694 | 6386 | 7236 | 6942 | 7363 | 6316 | 0.187346651 | 0.65166286 |
| BJK46_006445 | 272 | 330 | 312 | 298 | 707 | 436 | -0.06075628 | 0.02628583 |
| BJK46_006450 | 12459 | 11782 | 12485 | 1744 | 2010 | 1808 | -2.66583938 | 2.22E-215 |
| BJK46_006455 | 8304 | 8659 | 8699 | 7142 | 6692 | 7213 | -0.17117404 | 7.15E-05 |
| BJK46_006460 | 2451 | 2584 | 3103 | 3382 | 3888 | 2981 | 0.363924437 | 0.00371815 |
| BJK46_006465 | 1713 | 1852 | 1994 | 1843 | 2119 | 1809 | 0.061889182 | 0.91656598 |
| BJK46_006470 | 244 | 262 | 304 | 415 | 812 | 496 | 0.420400491 | 1.56E-05 |
| BJK46_006475 | 69257 | 75891 | 84375 | 34548 | 32849 | 31705 | -0.84684453 | 1.25E-30 |
| BJK46_006480 | 3398 | 3485 | 3626 | 3753 | 3572 | 3116 | 0.027089531 | 0.43824819 |
| BJK46_006485 | 8893 | 11913 | 15521 | 5123 | 8612 | 6294 | -0.7171963 | 1.31E-09 |
| BJK46_006490 | 2512 | 2628 | 3308 | 2048 | 3637 | 2715 | -0.00935452 | 0.56589587 |
| BJK46_006495 | 2431 | 2848 | 2609 | 1427 | 1639 | 1695 | -0.71438951 | 2.65E-16 |
| BJK46_006500 | 250 | 231 | 230 | 322 | 678 | 412 | 0.226728192 | 0.00038359 |
| BJK46_006505 | 89 | 97 | 86 | 218 | 554 | 266 | 0.788651852 | 4.18E-09 |
| BJK46_006510 | 118 | 118 | 109 | 246 | 523 | 297 | 0.605438055 | 2.54E-08 |
| BJK46_006515 | 136 | 122 | 125 | 267 | 591 | 368 | 0.718696212 | 5.33E-09 |
| BJK46_006520 | 255 | 245 | 291 | 362 | 728 | 471 | 0.281002362 | 0.00015448 |
| BJK46_006525 | 4829 | 4936 | 4545 | 1271 | 1400 | 1364 | -1.80176579 | 1.01E-89 |
| BJK46_006530 | 1808 | 1619 | 1872 | 579 | 787 | 611 | -1.58117359 | 3.93E-26 |
| BJK46_006535 | 1718 | 1755 | 1949 | 681 | 910 | 621 | -1.43171003 | 1.02E-22 |
| BJK46_006540 | 1542 | 1543 | 1740 | 562 | 909 | 537 | -1.47410723 | 4.49E-15 |
| BJK46_006545 | 1973 | 2000 | 2211 | 710 | 882 | 696 | -1.52986385 | 1.25E-37 |
| BJK46_006550 | 3407 | 3697 | 3479 | 2485 | 2578 | 2709 | -0.41349306 | 6.22E-09 |
| BJK46_006555 | 361 | 383 | 445 | 540 | 888 | 571 | 0.230873854 | 0.00098975 |
| BJK46_006560 | 261 | 273 | 298 | 398 | 618 | 450 | 0.154552491 | 0.00030315 |
| BJK46_006565 | 234 | 252 | 285 | 440 | 830 | 578 | 0.609117071 | 1.27E-07 |
| BJK46_006570 | 7258 | 11192 | 8122 | 2425 | 3098 | 3222 | -1.5271906 | 6.72E-35 |
| BJK46_006575 | 869 | 1010 | 1153 | 1220 | 1486 | 1343 | 0.306065929 | 0.00543947 |
| BJK46_006580 | 13879 | 15314 | 17897 | 15174 | 15940 | 12998 | 0.143289872 | 0.14351606 |
| BJK46_006585 | 574 | 635 | 593 | 1091 | 1233 | 1292 | 0.752599801 | 3.25E-10 |
| BJK46_006590 | 299 | 400 | 282 | 645 | 589 | 864 | 0.574378762 | 6.26E-06 |
| BJK46_006595 | 676 | 894 | 693 | 1491 | 1279 | 1861 | 0.876383091 | 5.82E-08 |
| BJK46_006600 | 184 | 195 | 220 | 304 | 358 | 342 | -0.06139452 | 0.00087037 |
| BJK46_006605 | 212 | 263 | 248 | 466 | 593 | 530 | 0.483336976 | 3.81E-08 |
| BJK46_006610 | 3116 | 3588 | 3625 | 5948 | 5629 | 5775 | 0.806627549 | 3.10E-17 |
| BJK46_006615 | 15624 | 16429 | 16730 | 11982 | 11435 | 11639 | -0.25430216 | 2.05E-08 |
| BJK46_006620 | 3090 | 3257 | 2967 | 1370 | 1543 | 1444 | -1.07883827 | 7.38E-34 |
| BJK46_006625 | 574 | 651 | 626 | 1192 | 1379 | 1296 | 0.83383193 | 1.46E-12 |
| BJK46_006630 | 275 | 197 | 278 | 224 | 543 | 319 | -0.29489983 | 0.11751694 |
| BJK46_006635 | 111 | 97 | 130 | 163 | 434 | 244 | 0.187984283 | 5.85E-05 |
| BJK46_006640 | 341 | 432 | 480 | 584 | 1414 | 764 | 0.640166682 | 3.01E-05 |
| BJK46_006645 | 103 | 155 | 191 | 170 | 393 | 205 | -0.2494453 | 0.01841139 |
| BJK46_006650 | 250 | 286 | 386 | 377 | 867 | 494 | 0.259808853 | 0.00135061 |
| BJK46_006655 | 9340 | 9475 | 8883 | 6469 | 6351 | 6747 | -0.38562735 | 8.74E-10 |
| BJK46_006660 | 245 | 284 | 240 | 252 | 298 | 334 | -0.58119194 | 0.54339364 |
| BJK46_006665 | 853 | 1030 | 1174 | 1388 | 2922 | 1705 | 0.860506313 | 2.16E-06 |
| BJK46_006670 | 395 | 405 | 544 | 684 | 1338 | 818 | 0.65169909 | 4.68E-06 |
| BJK46_006675 | 190 | 225 | 284 | 346 | 683 | 382 | 0.26467885 | 0.00019917 |
| BJK46_006680 | 2695 | 2799 | 3318 | 2533 | 2954 | 2442 | -0.12348088 | 0.01228597 |
| BJK46_006685 | 4112 | 4281 | 5349 | 3534 | 4004 | 3413 | -0.27100109 | 4.21E-06 |
| BJK46_006690 | 7441 | 7920 | 9412 | 6332 | 7144 | 6399 | -0.2060091 | 1.29E-06 |
| BJK46_006695 | 4107 | 4378 | 5086 | 3673 | 3898 | 3368 | -0.25797551 | 3.82E-06 |
| BJK46_006700 | 349 | 351 | 363 | 590 | 1037 | 701 | 0.61209022 | 4.41E-07 |
| BJK46_006705 | 1511 | 1558 | 1664 | 3568 | 4175 | 3664 | 1.289868054 | 1.50E-48 |
| BJK46_006710 | 20 | 10 | 2 | 61 | 141 | 80 | 1.463038146 | 8.44E-11 |
| BJK46_006715 | 694 | 749 | 553 | 842 | 934 | 1189 | 0.292308383 | 0.01062504 |
| BJK46_006720 | 16443 | 17865 | 14889 | 13808 | 15074 | 16467 | 0.122649471 | 0.11369669 |
| BJK46_006725 | 14588 | 15558 | 14947 | 17364 | 19461 | 21388 | 0.612689901 | 0.00455848 |
| BJK46_006730 | 22271 | 23976 | 28238 | 14433 | 15138 | 13895 | -0.51120362 | 4.84E-17 |
| BJK46_006735 | 4608 | 5353 | 4985 | 2294 | 2176 | 2528 | -1.0558859 | 9.16E-34 |
| BJK46_006740 | 4896 | 4998 | 5622 | 3826 | 3879 | 3658 | -0.39226944 | 1.93E-11 |
| BJK46_006745 | 2366 | 2762 | 2929 | 1907 | 2136 | 1926 | -0.4076936 | 2.82E-09 |
| BJK46_006750 | 3030 | 3129 | 3437 | 1732 | 1759 | 1767 | -0.84355873 | 2.39E-28 |
| BJK46_006755 | 661 | 642 | 802 | 589 | 707 | 598 | -0.51232068 | 0.19989961 |
| BJK46_006760 | 835 | 799 | 1008 | 698 | 831 | 732 | -0.49103977 | 0.06078648 |
| BJK46_006765 | 11486 | 12313 | 13897 | 14661 | 14823 | 13571 | 0.412706928 | 0.1380303 |
| BJK46_006770 | 4379 | 4728 | 5262 | 2488 | 2549 | 2336 | -0.92013762 | 1.44E-38 |
| BJK46_006775 | 3202 | 3525 | 3946 | 1638 | 1945 | 1641 | -1.0061971 | 5.92E-39 |
| BJK46_006780 | 797 | 869 | 976 | 457 | 536 | 357 | -1.37253775 | 1.51E-08 |
| BJK46_006785 | 3569 | 4082 | 4435 | 3059 | 3222 | 2982 | -0.33782701 | 9.90E-09 |
| BJK46_006790 | 16356 | 17573 | 21043 | 14195 | 14753 | 12878 | -0.14685357 | 7.47E-06 |
| BJK46_006795 | 1285 | 1348 | 1463 | 835 | 992 | 725 | -0.84121836 | 1.03E-07 |
| BJK46_006800 | 1510 | 1522 | 1678 | 963 | 1125 | 885 | -0.7601865 | 3.64E-09 |
| BJK46_006805 | 1205 | 1275 | 1384 | 738 | 892 | 732 | -0.89030826 | 1.19E-08 |
| BJK46_006810 | 4062 | 4467 | 4723 | 113950 | 98869 | 113617 | 4.895426073 | 2.79E-249 |
| BJK46_006815 | 2452 | 2740 | 2855 | 2113 | 2214 | 2057 | -0.30916311 | 1.25E-06 |
| BJK46_006820 | 1440 | 1661 | 1508 | 1475 | 1931 | 1952 | 0.197271178 | 0.20531477 |
| BJK46_006825 | 8026 | 8450 | 8821 | 15031 | 13531 | 15375 | 0.979411716 | 3.01E-14 |
| BJK46_006830 | 98 | 101 | 76 | 179 | 306 | 234 | 0.263187121 | 1.11E-06 |
| BJK46_006835 | 37658 | 40745 | 38576 | 21447 | 19354 | 20773 | -0.62800655 | 5.25E-18 |
| BJK46_006840 | 171 | 139 | 140 | 221 | 501 | 322 | 0.25654887 | 6.23E-05 |
| BJK46_006845 | 3048 | 3201 | 3152 | 2518 | 2801 | 3074 | -0.13726826 | 0.01347405 |
| BJK46_006850 | 2170 | 2228 | 2242 | 1772 | 2001 | 2218 | -0.13743414 | 0.03373238 |
| BJK46_006855 | 2504 | 2437 | 3004 | 2575 | 2783 | 2594 | 0.032819805 | 0.4870975 |
| BJK46_006860 | 5354 | 5711 | 6413 | 6449 | 6694 | 6450 | 0.248608771 | 0.15250275 |
| BJK46_006865 | 3921 | 4484 | 4583 | 6731 | 7184 | 7357 | 0.783394537 | 3.57E-18 |
| BJK46_006870 | 999 | 1052 | 1131 | 2154 | 2379 | 2310 | 1.081482557 | 1.33E-26 |
| BJK46_006875 | 97 | 141 | 138 | 201 | 449 | 286 | 0.297752103 | 8.19E-06 |
| BJK46_006880 | 667 | 866 | 856 | 10536 | 12130 | 13079 | 3.939358232 | 2.70876531121167e-310 |
| BJK46_006885 | 3570 | 3816 | 3890 | 3277 | 3424 | 3638 | -0.0832193 | 0.02048384 |
| BJK46_006890 | 4801 | 4921 | 5446 | 4102 | 4396 | 3865 | -0.23821457 | 2.09E-06 |
| BJK46_006895 | 950 | 1039 | 1222 | 1621 | 1931 | 1649 | 0.63752384 | 4.99E-08 |
| BJK46_006900 | 617 | 683 | 710 | 1208 | 1684 | 1294 | 0.849809791 | 1.83E-11 |
| BJK46_006905 | 1870 | 1477 | 1552 | 2284 | 2504 | 2224 | 0.534371511 | 0.00031749 |
| BJK46_006910 | 2563 | 1650 | 1964 | 2014 | 2392 | 1912 | 0.065263024 | 0.75367537 |
| BJK46_006915 | 3487 | 2986 | 3213 | 1492 | 1753 | 1479 | -1.01708192 | 4.32E-26 |
| BJK46_006920 | 441 | 397 | 458 | 682 | 975 | 819 | 0.507384005 | 2.06E-06 |
| BJK46_006925 | 13360 | 14832 | 15126 | 29468 | 25928 | 30990 | 1.260470717 | 3.02E-16 |
| BJK46_006930 | 4514 | 4942 | 5152 | 5710 | 6007 | 5502 | 0.304132824 | 0.01463057 |
| BJK46_006935 | 2288 | 2831 | 2455 | 5418 | 5608 | 6068 | 1.223809803 | 2.95E-32 |
| BJK46_006940 | 1315 | 1427 | 1501 | 1750 | 2008 | 1788 | 0.370465244 | 0.00094092 |
| BJK46_006945 | 5194 | 5379 | 5781 | 4237 | 4422 | 4500 | -0.25196663 | 3.44E-07 |
| BJK46_006950 | 2198 | 2237 | 2582 | 1724 | 1916 | 1732 | -0.36832125 | 2.94E-07 |
| BJK46_006955 | 11813 | 12086 | 13036 | 14340 | 13563 | 14441 | 0.412615834 | 0.14513542 |
| BJK46_006960 | 8145 | 8444 | 9216 | 9771 | 9607 | 8816 | 0.270795851 | 0.41949545 |
| BJK46_006965 | 12496 | 14079 | 12194 | 13334 | 12054 | 14570 | 0.255794782 | 0.87315404 |
| BJK46_006970 | 638 | 720 | 709 | 737 | 987 | 795 | -0.02550515 | 0.17711798 |
| BJK46_006975 | 9071 | 9490 | 10123 | 10968 | 11048 | 11146 | 0.379693686 | 0.06664555 |
| BJK46_006980 | 1820 | 1962 | 2052 | 1734 | 1892 | 1582 | -0.1607226 | 0.01538594 |
| BJK46_006985 | 1182 | 1316 | 1400 | 1233 | 1398 | 1106 | -0.14800576 | 0.3182191 |
| BJK46_006990 | 722 | 794 | 853 | 770 | 830 | 667 | -0.3597475 | 0.42400292 |
| BJK46_006995 | 13201 | 14287 | 12469 | 23937 | 23543 | 23945 | 1.09372107 | 3.20E-12 |
| BJK46_007000 | 227 | 270 | 268 | 424 | 602 | 394 | 0.204190594 | 0.00010096 |
| BJK46_007005 | 16023 | 18263 | 18654 | 10814 | 9938 | 11003 | -0.51507895 | 4.02E-15 |
| BJK46_007010 | 124 | 143 | 134 | 270 | 702 | 393 | 0.805588823 | 9.07E-09 |
| BJK46_007015 | 1515 | 1663 | 1669 | 1677 | 1989 | 1667 | 0.128218457 | 0.46827485 |
| BJK46_007020 | 521 | 565 | 522 | 2171 | 2688 | 2735 | 2.115633511 | 6.58E-68 |
| BJK46_007025 | 11506 | 11719 | 12460 | 15046 | 13818 | 14557 | 0.497000692 | 0.02102749 |
| BJK46_007030 | 3764 | 4183 | 4351 | 6567 | 6940 | 6241 | 0.748799422 | 1.32E-17 |
| BJK46_007035 | 754 | 868 | 944 | 1960 | 2049 | 1834 | 1.112918379 | 3.48E-22 |
| BJK46_007040 | 1496 | 1659 | 1576 | 1747 | 1894 | 1893 | 0.221334212 | 0.10267404 |
| BJK46_007045 | 1640 | 1766 | 1774 | 3152 | 3299 | 2984 | 0.886118336 | 2.57E-21 |
| BJK46_007050 | 514 | 572 | 569 | 242 | 367 | 278 | -1.51346 | 9.48E-07 |
| BJK46_007055 | 6347 | 6760 | 6987 | 11443 | 12179 | 12873 | 1.003725589 | 4.40E-22 |
| BJK46_007060 | 2439 | 2838 | 2758 | 2784 | 3057 | 3044 | 0.175218836 | 0.31805288 |
| BJK46_007065 | 794 | 907 | 940 | 1818 | 2156 | 2107 | 1.135465572 | 2.83E-24 |
| BJK46_007070 | 433 | 442 | 453 | 758 | 772 | 773 | 0.372417626 | 1.97E-05 |
| BJK46_007075 | 138 | 160 | 139 | 252 | 493 | 310 | 0.332038988 | 3.44E-06 |
| BJK46_007080 | 162 | 190 | 156 | 283 | 569 | 375 | 0.408415013 | 3.02E-06 |
| BJK46_007085 | 1354 | 1539 | 1597 | 3074 | 3323 | 2708 | 1.035908864 | 7.28E-25 |
| BJK46_007090 | 3059 | 3310 | 3781 | 6087 | 6532 | 5801 | 0.919970947 | 9.02E-27 |
| BJK46_007095 | 14160 | 15671 | 17599 | 14400 | 14206 | 13524 | 0.066855705 | 0.01876224 |
| BJK46_007100 | 3164 | 3353 | 3395 | 6174 | 6464 | 6249 | 0.987045767 | 7.70E-32 |
| BJK46_007105 | 29407 | 31454 | 31529 | 25343 | 20853 | 24050 | -0.10637599 | 0.0001714 |
| BJK46_007110 | 13606 | 14350 | 14029 | 27738 | 27425 | 30180 | 1.28704061 | 3.48E-19 |
| BJK46_007115 | 648 | 684 | 644 | 750 | 963 | 915 | 0.102133592 | 0.03519495 |
| BJK46_007120 | 1137 | 1234 | 1343 | 2904 | 3321 | 2685 | 1.269983912 | 3.29E-38 |
| BJK46_007125 | 35423 | 36786 | 40933 | 31901 | 27223 | 26514 | -0.09036709 | 0.00010371 |
| BJK46_007130 | 1399 | 1462 | 1716 | 1317 | 1688 | 1349 | -0.10975389 | 0.23214284 |
| BJK46_007135 | 893 | 981 | 1174 | 860 | 1125 | 915 | -0.25720224 | 0.35373519 |
| BJK46_007140 | 2242 | 2578 | 2304 | 6657 | 6076 | 6861 | 1.508207666 | 9.79E-46 |
| BJK46_007145 | 311 | 351 | 364 | 181 | 262 | 212 | -1.43994513 | 0.00064706 |
| BJK46_007150 | 8841 | 9298 | 9393 | 7318 | 6514 | 7017 | -0.28163461 | 6.00E-07 |
| BJK46_007155 | 1551 | 1816 | 2032 | 1398 | 1505 | 1410 | -0.33264905 | 0.00016148 |
| BJK46_007160 | 3932 | 4411 | 5126 | 2964 | 3185 | 2872 | -0.52759065 | 1.20E-15 |
| BJK46_007165 | 451 | 468 | 466 | 594 | 1001 | 698 | 0.273524685 | 0.00103199 |
| BJK46_007170 | 340 | 321 | 325 | 377 | 825 | 491 | 0.127597244 | 0.00532395 |
| BJK46_007175 | 1093 | 1224 | 1274 | 991 | 1375 | 1059 | -0.19607754 | 0.3135924 |
| BJK46_007180 | 25951 | 27586 | 23574 | 27708 | 27593 | 34761 | 0.514584959 | 0.23899678 |
| BJK46_007185 | 1310 | 1350 | 1399 | 1910 | 2140 | 2111 | 0.591966605 | 3.86E-08 |
| BJK46_007190 | 8806 | 9523 | 10937 | 8125 | 8653 | 8258 | -0.07683932 | 0.00028153 |
| BJK46_007195 | 4099 | 4483 | 5018 | 5253 | 5338 | 4792 | 0.240757057 | 0.12139278 |
| BJK46_007200 | 7468 | 9341 | 6420 | 20098 | 18950 | 25792 | 1.695004814 | 2.27E-18 |
| BJK46_007205 | 7615 | 7996 | 9465 | 3528 | 3924 | 3350 | -1.14327892 | 1.72E-59 |
| BJK46_007210 | 1559 | 1749 | 1774 | 2352 | 2578 | 2377 | 0.53932238 | 7.69E-08 |
| BJK46_007215 | 2690 | 2943 | 2855 | 4451 | 4826 | 4476 | 0.738492577 | 4.25E-16 |
| BJK46_007220 | 22748 | 24878 | 29029 | 45973 | 48178 | 42383 | 1.155840662 | 2.11E-13 |
| BJK46_007225 | 11510 | 12932 | 14804 | 24197 | 26433 | 22562 | 1.157311101 | 4.51E-17 |
| BJK46_007230 | 128 | 138 | 159 | 211 | 562 | 318 | 0.355289025 | 1.42E-05 |
| BJK46_007235 | 162 | 145 | 177 | 203 | 563 | 337 | 0.212195889 | 0.00022 |
| BJK46_007240 | 348 | 308 | 381 | 347 | 891 | 513 | 0.091936227 | 0.01337923 |
| BJK46_007245 | 307 | 281 | 404 | 310 | 761 | 435 | -0.08689517 | 0.05371143 |
| BJK46_007250 | 173 | 216 | 260 | 229 | 511 | 332 | -0.11956691 | 0.01312203 |
| BJK46_007255 | 157 | 186 | 203 | 196 | 478 | 249 | -0.21103396 | 0.01716326 |
| BJK46_007260 | 256 | 292 | 306 | 237 | 618 | 355 | -0.29770975 | 0.12936813 |
| BJK46_007265 | 322 | 337 | 361 | 285 | 723 | 412 | -0.23969177 | 0.14287647 |
| BJK46_007270 | 215 | 180 | 236 | 198 | 513 | 271 | -0.28898599 | 0.05922967 |
| BJK46_007275 | 351 | 376 | 361 | 605 | 987 | 671 | 0.546317669 | 1.16E-06 |
| BJK46_007280 | 1406 | 1404 | 1580 | 2528 | 3063 | 2228 | 0.833641259 | 2.57E-13 |
| BJK46_007285 | 1126 | 1168 | 1286 | 2321 | 2969 | 2350 | 1.08362927 | 4.34E-25 |
| BJK46_007290 | 2199 | 2497 | 2413 | 2427 | 2489 | 2487 | 0.083385664 | 0.96892911 |
| BJK46_007295 | 923 | 865 | 890 | 681 | 1172 | 874 | -0.23716252 | 0.76257947 |
| BJK46_007300 | 863 | 867 | 896 | 847 | 1256 | 921 | -0.02579975 | 0.41318825 |
| BJK46_007305 | 5653 | 6324 | 6863 | 6417 | 7140 | 6467 | 0.176323332 | 0.70689653 |
| BJK46_007310 | 738 | 876 | 965 | 1297 | 1497 | 1237 | 0.497721913 | 1.54E-05 |
| BJK46_007315 | 228 | 230 | 241 | 438 | 876 | 553 | 0.726618809 | 1.87E-08 |
| BJK46_007320 | 1758 | 1878 | 1828 | 3921 | 4031 | 4089 | 1.166927379 | 1.07E-36 |
| BJK46_007325 | 987 | 1125 | 1018 | 1040 | 1391 | 1128 | 0.040774838 | 0.4200772 |
| BJK46_007330 | 682 | 744 | 716 | 844 | 1020 | 867 | 0.072112025 | 0.06290409 |
| BJK46_007335 | 993 | 997 | 1059 | 801 | 1158 | 870 | -0.31612076 | 0.23456442 |
| BJK46_007340 | 503 | 541 | 524 | 1505 | 1622 | 1463 | 1.321697125 | 3.42E-26 |
| BJK46_007345 | 616 | 668 | 811 | 543 | 1233 | 737 | -0.1235745 | 0.41968347 |
| BJK46_007350 | 2810 | 3145 | 2992 | 4061 | 4213 | 4139 | 0.514468799 | 4.01E-07 |
| BJK46_007355 | 1672 | 1667 | 1967 | 3143 | 3621 | 2887 | 0.883228184 | 1.66E-18 |
| BJK46_007360 | 2062 | 2145 | 2532 | 2488 | 2710 | 2055 | 0.123695521 | 0.65896447 |
| BJK46_007365 | 90780 | 92215 | 89351 | 132459 | 118018 | 121413 | 0.888105693 | 0.00115243 |
| BJK46_007370 | 38876 | 41653 | 36908 | 85844 | 72985 | 80489 | 1.398605478 | 4.86E-14 |
| BJK46_007375 | 3223 | 3281 | 3413 | 4098 | 4408 | 4372 | 0.42175669 | 5.57E-05 |
| BJK46_007380 | 5533 | 5929 | 6453 | 3507 | 3558 | 3569 | -0.69251455 | 2.15E-28 |
| BJK46_007385 | 14492 | 16010 | 18208 | 3869 | 4229 | 3742 | -1.91535275 | 6.61E-140 |
| BJK46_007390 | 18912 | 21567 | 23401 | 4114 | 4568 | 3976 | -2.17972636 | 1.97E-150 |
| BJK46_007395 | 59 | 52 | 23 | 183 | 514 | 226 | 1.472757 | 1.21E-10 |
| BJK46_007400 | 1730 | 1765 | 1822 | 1005 | 1380 | 1206 | -0.61795101 | 7.35E-08 |
| BJK46_007405 | 3693 | 3976 | 3940 | 1889 | 2496 | 2076 | -0.82476111 | 9.54E-25 |
| BJK46_007410 | 3303 | 3677 | 3473 | 1581 | 1755 | 1628 | -1.05144276 | 4.41E-40 |
| BJK46_007415 | 1976 | 2061 | 1992 | 7842 | 7122 | 7519 | 1.944827798 | 2.95E-87 |
| BJK46_007420 | 10090 | 10650 | 11689 | 19197 | 19451 | 17120 | 1.008348498 | 1.70E-13 |
| BJK46_007425 | 7439 | 7439 | 8254 | 1245 | 1475 | 1231 | -2.51795019 | 7.06E-223 |
| BJK46_007430 | 25815 | 31792 | 34196 | 107100 | 106821 | 98043 | 2.147366803 | 1.44E-51 |
| BJK46_007435 | 1671 | 2081 | 2115 | 1184 | 1459 | 1317 | -0.58132742 | 1.80E-09 |
| BJK46_007440 | 1836 | 2051 | 1958 | 4117 | 4426 | 4400 | 1.177254054 | 1.25E-39 |
| BJK46_007445 | 307 | 350 | 371 | 450 | 598 | 549 | 0.061971216 | 0.00227993 |
| BJK46_007450 | 261 | 270 | 303 | 461 | 1010 | 638 | 0.70314818 | 2.45E-07 |
| BJK46_007455 | 133 | 118 | 110 | 350 | 948 | 518 | 1.403266521 | 5.35E-13 |
| BJK46_007460 | 134 | 147 | 125 | 407 | 840 | 492 | 1.250238067 | 2.73E-14 |
| BJK46_007465 | 859 | 961 | 1140 | 6845 | 7266 | 5691 | 2.7681757 | 2.63E-156 |
| BJK46_007470 | 1988 | 2209 | 2648 | 18025 | 19413 | 14719 | 3.011820512 | 6.79E-156 |
| BJK46_007475 | 59 | 64 | 48 | 90 | 148 | 87 | -0.54330126 | 0.00388933 |
| BJK46_007480 | 3399 | 3181 | 2823 | 3400 | 3349 | 3222 | 0.123777313 | 0.9035953 |
| BJK46_007485 | 853 | 819 | 728 | 918 | 860 | 860 | -0.11678877 | 0.68629758 |
| BJK46_007490 | 1169 | 1081 | 1031 | 1045 | 1172 | 1127 | -0.10974012 | 0.75808986 |
| BJK46_007495 | 4052 | 3912 | 3483 | 3097 | 3536 | 3020 | -0.20588148 | 0.00181932 |
| BJK46_007500 | 20270 | 22638 | 28013 | 29776 | 27905 | 28034 | 0.575351695 | 0.04917863 |
| BJK46_007505 | 18782 | 20942 | 26089 | 26951 | 25174 | 25303 | 0.528373051 | 0.10397549 |
| BJK46_007510 | 12832 | 13354 | 17551 | 17836 | 18117 | 18281 | 0.567724412 | 0.0164911 |
| BJK46_007515 | 11328 | 12681 | 15373 | 18246 | 16496 | 17863 | 0.66265564 | 0.00085408 |
| BJK46_007520 | 112 | 163 | 142 | 230 | 399 | 310 | 0.227579627 | 8.32E-06 |
| BJK46_007525 | 12695 | 13822 | 16033 | 7288 | 8057 | 7037 | -0.75382215 | 2.61E-32 |
| BJK46_007530 | 4512 | 5264 | 6593 | 2924 | 3442 | 2783 | -0.777871 | 1.29E-20 |
| BJK46_007535 | 8170 | 9771 | 7426 | 14028 | 11494 | 14867 | 0.847065641 | 8.09E-06 |
| BJK46_007540 | 11841 | 12513 | 14007 | 24614 | 23346 | 23293 | 1.147614278 | 1.31E-16 |
| BJK46_007545 | 0 | 2 | 1 | 8 | 32 | 15 | 0.809751869 | 6.21E-09 |
| BJK46_007550 | 8406 | 8471 | 9263 | 30573 | 33095 | 31241 | 2.106844532 | 4.65E-73 |
| BJK46_007555 | 30445 | 34534 | 35838 | 26375 | 27003 | 27105 | -0.01718213 | 0.00013067 |
| BJK46_007560 | 13110 | 19134 | 10603 | 14315 | 8844 | 11542 | -0.08135313 | 0.08869067 |
| BJK46_007565 | 10355 | 11099 | 13294 | 10109 | 10595 | 8870 | -0.05257832 | 0.00128829 |
| BJK46_007570 | 17416 | 18414 | 22111 | 16663 | 17433 | 14806 | 0.012452229 | 0.00310353 |
| BJK46_007575 | 1757 | 1753 | 1780 | 1818 | 1813 | 1940 | 0.077172901 | 0.91728919 |
| BJK46_007580 | 7619 | 7911 | 8554 | 3573 | 3032 | 2959 | -1.2732299 | 3.43E-46 |
| BJK46_007585 | 10128 | 10202 | 11368 | 4079 | 3650 | 3713 | -1.38625164 | 9.11E-63 |
| BJK46_007590 | 11383 | 11687 | 12601 | 4232 | 3784 | 3797 | -1.50081007 | 1.17E-71 |
| BJK46_007595 | 10141 | 10784 | 11097 | 3447 | 3130 | 3247 | -1.62955025 | 3.80E-87 |
| BJK46_007600 | 17205 | 18540 | 17838 | 6333 | 5459 | 6429 | -1.38792339 | 3.46E-50 |
| BJK46_007605 | 10674 | 11049 | 12058 | 10290 | 10993 | 11022 | 0.119155506 | 0.11567401 |
| BJK46_007610 | 107860 | 116298 | 83656 | 139257 | 138063 | 195723 | 1.069897763 | 0.00152606 |
| BJK46_007615 | 19451 | 21438 | 18853 | 26893 | 26007 | 32338 | 0.789237526 | 0.00035518 |
| BJK46_007620 | 124 | 137 | 117 | 190 | 355 | 223 | -0.02795121 | 0.00033387 |
| BJK46_007625 | 287 | 310 | 282 | 350 | 497 | 367 | -0.21992446 | 0.05906597 |
| BJK46_007630 | 53 | 59 | 56 | 179 | 441 | 268 | 1.117494432 | 9.27E-14 |
| BJK46_007635 | 36844 | 40379 | 44558 | 34646 | 36312 | 33186 | 0.105935671 | 0.00515896 |
| BJK46_007640 | 746 | 932 | 901 | 662 | 721 | 806 | -0.51715735 | 0.06449717 |
| BJK46_007645 | 5053 | 5754 | 5853 | 6507 | 6691 | 6938 | 0.354744933 | 0.00489219 |
| BJK46_007650 | 3609 | 4089 | 4520 | 5327 | 5837 | 5831 | 0.540293036 | 1.52E-08 |
| BJK46_007655 | 1112 | 1204 | 1283 | 2527 | 3100 | 2770 | 1.223961397 | 9.82E-38 |
| BJK46_007660 | 2652 | 2974 | 3506 | 3334 | 3806 | 3533 | 0.268740429 | 0.04060578 |
| BJK46_007665 | 5965 | 6575 | 7823 | 7434 | 8017 | 7179 | 0.261017022 | 0.24203704 |
| BJK46_007670 | 7794 | 8266 | 9849 | 9122 | 9136 | 8242 | 0.174694739 | 0.77260407 |
| BJK46_007675 | 3682 | 3670 | 4787 | 3001 | 3246 | 2902 | -0.35748003 | 1.49E-07 |
| BJK46_007680 | 7789 | 8190 | 9655 | 5177 | 5315 | 5159 | -0.61592415 | 6.59E-23 |
| BJK46_007685 | 11420 | 11643 | 12813 | 9461 | 8222 | 8558 | -0.28410584 | 1.15E-07 |
| BJK46_007690 | 9519 | 11110 | 10883 | 9426 | 9602 | 9860 | 0.042145496 | 0.0252987 |
| BJK46_007695 | 342 | 382 | 370 | 286 | 513 | 348 | -0.60823615 | 0.97163522 |
| BJK46_007700 | 460 | 498 | 451 | 552 | 744 | 593 | -0.04344551 | 0.05726204 |
| BJK46_007705 | 1034 | 1204 | 1101 | 1188 | 1337 | 1367 | 0.116722806 | 0.22900886 |
| BJK46_007710 | 13016 | 14562 | 14346 | 8734 | 7062 | 8485 | -0.61713508 | 1.37E-14 |
| BJK46_007715 | 3285 | 3418 | 3443 | 1657 | 1834 | 1526 | -0.99728699 | 3.48E-34 |
| BJK46_007720 | 2208 | 2441 | 2478 | 1859 | 2018 | 1908 | -0.28175414 | 1.73E-05 |
| BJK46_007725 | 37035 | 41546 | 29510 | 29249 | 30503 | 43738 | 0.247317834 | 0.44789592 |
| BJK46_007730 | 690 | 719 | 798 | 558 | 725 | 599 | -0.58854726 | 0.07120014 |
| BJK46_007735 | 713 | 784 | 896 | 598 | 819 | 621 | -0.55716871 | 0.07304004 |
| BJK46_007740 | 1331 | 1470 | 1584 | 1466 | 1828 | 1475 | 0.088494555 | 0.60946387 |
| BJK46_007745 | 4416 | 4735 | 5142 | 7692 | 7781 | 7311 | 0.75201244 | 3.22E-16 |
| BJK46_007750 | 6426 | 6967 | 7363 | 10727 | 11197 | 10257 | 0.768251301 | 1.89E-13 |
| BJK46_007755 | 676 | 799 | 719 | 764 | 762 | 921 | -0.1388463 | 0.56649984 |
| BJK46_007760 | 8190 | 9221 | 10966 | 11390 | 12545 | 10752 | 0.468715534 | 0.00752693 |
| BJK46_007765 | 2576 | 2988 | 3390 | 3163 | 3336 | 3101 | 0.141609512 | 0.59799506 |
| BJK46_007770 | 12955 | 14296 | 15684 | 13952 | 14420 | 13576 | 0.196008149 | 0.30277754 |
| BJK46_007775 | 3301 | 3366 | 3158 | 3095 | 3022 | 3285 | -0.02894046 | 0.1745849 |
| BJK46_007780 | 8113 | 9052 | 8848 | 13116 | 13601 | 14463 | 0.843720028 | 3.29E-12 |
| BJK46_007785 | 7508 | 8253 | 9408 | 7014 | 7068 | 6661 | -0.16134521 | 2.03E-05 |
| BJK46_007790 | 45351 | 49469 | 53316 | 68664 | 62217 | 66356 | 0.790158118 | 0.00130381 |
| BJK46_007795 | 16190 | 18643 | 19746 | 25975 | 23590 | 24992 | 0.724325616 | 0.00022249 |
| BJK46_007800 | 35109 | 38533 | 44302 | 54028 | 50951 | 51254 | 0.760735938 | 0.001356 |
| BJK46_007805 | 38562 | 43608 | 49402 | 65483 | 65877 | 63003 | 0.937529427 | 2.25E-06 |
| BJK46_007810 | 5338 | 6434 | 7556 | 12948 | 13782 | 13330 | 1.213206742 | 1.50E-28 |
| BJK46_007815 | 2853 | 3305 | 3216 | 4420 | 4630 | 4791 | 0.609924892 | 3.12E-10 |
| BJK46_007820 | 173 | 184 | 192 | 439 | 1002 | 576 | 1.117470903 | 8.00E-12 |
| BJK46_007825 | 124 | 163 | 149 | 162 | 373 | 202 | -0.309559 | 0.01767922 |
| BJK46_007830 | 956 | 1030 | 1192 | 987 | 1124 | 980 | -0.19828171 | 0.45345278 |
| BJK46_007835 | 2425 | 2538 | 2691 | 2542 | 2833 | 2563 | 0.077544284 | 0.88614501 |
| BJK46_007840 | 6746 | 7202 | 6859 | 5355 | 5069 | 5622 | -0.29694777 | 9.74E-07 |
| BJK46_007845 | 5854 | 6833 | 7067 | 6977 | 6693 | 6758 | 0.143284403 | 0.91273979 |
| BJK46_007850 | 30986 | 33341 | 36187 | 37322 | 39286 | 40660 | 0.548741426 | 0.11320674 |
| BJK46_007855 | 13268 | 13945 | 13405 | 16159 | 17287 | 19956 | 0.62767173 | 0.00279569 |
| BJK46_007860 | 12422 | 13280 | 11721 | 16127 | 16920 | 21021 | 0.756131552 | 0.00021625 |
| BJK46_007865 | 8499 | 9614 | 10737 | 9220 | 9399 | 8562 | 0.068963483 | 0.0801827 |
| BJK46_007870 | 3028 | 3529 | 3987 | 2773 | 2787 | 2430 | -0.36161159 | 3.42E-07 |
| BJK46_007875 | 7045 | 8259 | 9264 | 5656 | 6304 | 5429 | -0.39773768 | 4.88E-12 |
| BJK46_007880 | 6030 | 6520 | 8046 | 4327 | 5142 | 4154 | -0.5220713 | 2.16E-14 |
| BJK46_007885 | 7605 | 8597 | 9811 | 5560 | 6320 | 5620 | -0.46637513 | 6.28E-16 |
| BJK46_007890 | 10550 | 12155 | 12836 | 10168 | 10913 | 10286 | 0.008181174 | 0.00249295 |
| BJK46_007895 | 22011 | 23247 | 26999 | 24187 | 25212 | 23457 | 0.299485652 | 0.63654625 |
| BJK46_007900 | 14 | 16 | 12 | 47 | 114 | 56 | 0.318485076 | 2.18E-09 |
| BJK46_007905 | 6305 | 6825 | 7650 | 4020 | 4140 | 3874 | -0.7185836 | 3.39E-30 |
| BJK46_007910 | 3845 | 4158 | 4966 | 2252 | 2557 | 2236 | -0.84029837 | 5.95E-30 |
| BJK46_007915 | 5672 | 5978 | 7089 | 3188 | 3292 | 2993 | -0.92358028 | 1.59E-40 |
| BJK46_007920 | 7201 | 7625 | 8710 | 4107 | 4180 | 3940 | -0.86962378 | 4.32E-40 |
| BJK46_007925 | 127 | 119 | 149 | 199 | 364 | 222 | -0.03930091 | 0.00034706 |
| BJK46_007930 | 1383 | 1518 | 1783 | 855 | 1013 | 849 | -0.89144375 | 1.88E-11 |
| BJK46_007935 | 7178 | 8189 | 8892 | 9026 | 9157 | 8648 | 0.282120259 | 0.27076656 |
